# Effect of Cepharanthine on the Stemness of Lung Squamous Cell Carcinoma Based on Network Pharmacology and Bioinformatics

**DOI:** 10.1155/2022/5956526

**Published:** 2022-11-28

**Authors:** Bo Liu, Jian-xiong Deng

**Affiliations:** ^1^Department of Oncology, Pingxiang Second People's Hospital, Pingxiang, Jiangxi 337000, China; ^2^Department of Oncology, Gaoxin Hospital of the First Affiliated Hospital of Nanchang University, Nanchang, Jiangxi 330096, China

## Abstract

**Background:**

Lung squamous cell carcinoma (LUSC) has poor survival prognosis and few clinical treatment options. We urgently need to explore new therapeutic drugs in clinical practice. Cepharanthine (CEP) has been shown to have anticancer effects in several tumors, but the mechanism of CEP in treating LUSC has not been reported.

**Methods:**

SwissTargetPrediction, PharmMapper, and GeneCards were used to identify targets of CEP and LUSC. Further topological analysis was used to obtain hub genes via Cytoscape. Molecular docking was carried out to verify the combination of CEP with hub targets. Based on bioinformatics, we first analyzed the expression and survival of hub targets in LUSC and further analyzed the correlation between hub targets and cancer stemness, immune cell infiltration, and tumor mutation burden (TMB).

**Results:**

A total of 41 targets were identified. Further topological analysis identified 6 hub genes: AURKA, CCNA2, CCNE1, CDK1, CHEK1, and PLK1. Molecular docking analysis showed that CEP had stable binding to all these 6 target proteins. In-depth bioinformatics analysis of these 6 targets showed that high expression of these targets were positively correlated with cancer stemness index and negatively correlated with tumor infiltrating immune cells. In immune subtype analysis, the expressions of these targets were significantly decreased in inflammatory tumors. In addition, we also found that the expressions of these targets were positively correlated with TMB.

**Conclusion:**

Based on multidisciplinary analysis, we preliminarily identified potential targets of CEP for LUSC treatment and suggested that CEP may play a role in regulating LUSC stemness.

## 1. Introduction

Lung cancer is the malignant tumor with the highest morbidity and mortality in the world. In 2020, up to 1,796,144 people died of lung cancer worldwide, accounting for 18% of cancer-related deaths [1]. Lung squamous cell carcinoma (LUSC) is a common subtype of lung cancer, accounting for about 25-30% of lung cancer. Almost 50% of LUSC patients have metastases at diagnosis [2]. Despite rapid advances of immunotherapy in recent years, the 5-year survival rate for LUSC stage III and IV disease is only 13% and 2%, respectively [3, 4]. Therefore, there is an urgent need for novel anticancer drugs that mediate the molecular mechanisms underlying LUSC progression.

With the development of Traditional Chinese medicine, its monomer components have become the focus of current scientific research. In the COVID-19 epidemic, cepharanthine (CEP) has been found to reverse endoplasmic reticulum stress and heat shock response induced by COVID-19 [5]. The anti-COVID-19 effect of CEP was also confirmed in the studies of Ohashi et al. [6] and Zhang et al. [7]. CEP is a kind of isoquinoline alkaloid extracted from Traditional Chinese medicine *Stephania epigaea* Lo [8]. Previous studies have confirmed that CEP has a variety of pharmacological properties, such as antioxidant, plasma membrane stabilization, anti-inflammatory, and immune regulation [9–12]. In the study of tumor mechanism, CEP has been found to inhibit tumor cell proliferation, migration, and antiangiogenesis. Gao et al. [13] found that CEP may induce apoptosis and autophagy of breast cancer cells through the AKT/mTOR signaling pathway. Uthaisar et al. [14] demonstrated that CEP inhibits the invasion and metastasis of cholangiocarcinoma by inhibiting ICAM-1 and MMP-2. In studies of lung adenocarcinoma, CEP can activate the P38 pathway in A549 cells to regulate autophagy, and thus inhibit the malignant biological behavior of A549 cells [15]. However, the study of CEP in LUSC has not been reported.

Network pharmacology, developed by Hopkins et al. [16], seeks to explore the multilevel interactions of disease, genes, and drugs based on systems biology, computational biology, and omics theory to evaluate the therapeutic effect of Traditional Chinese medicine on diseases. This study aims to explore the molecular mechanism of CEP in the treatment of LUSC through network pharmacology, bioinformatics, and molecular docking. Firstly, the protein targets and disease targets of LUSC were predicted and integrated through databases of network pharmacology. Then, functional enrichment analysis was performed for the above target genes, and the protein interaction network diagram was constructed to screen the key targets based on topological analysis. And molecular docking was used to verify the binding of CEP to the target protein. To further explore the mechanism of CEP in treating LUSC, we conducted an in-depth bioinformatics analysis. It mainly includes the correlation between target and cancer stemness, tumor microenvironment, and tumor mutation burden. Our study has been published as a preprint on the Research Square (https://www.researchsquare.com/article/rs-1739051/v1) [17].

## 2. Materials and Methods

### 2.1. Screening of Physicochemical Parameters and Potential Targets of CEP

TCMSP database [18] was used to obtain characteristic information and physicochemical parameters of CEP. PubChem database (https://pubchem.ncbi.nlm.nih.gov/) was used to obtain the CEP element three-dimensional SDF files and SMELL. SwissTargetPrediction [19] website (http://www.swisstargetprediction.ch/) and PharmMapper [20] database (http://lilab-ecust.cn/pharm Mapper/) were used to predict the target sites, and the results of the two databases were combined to obtain the potential target sites of CEP.

### 2.2. Potential Targets and Differential Expressed Gene Analysis of LUSC

“Lung squamous cell carcinoma” was used as the keyword for GeneCards database (https://www.genecards.org/) retrieval in LUSC of the related targets. RNAseq data (level 3) and corresponding clinical information of LUSC were obtained from the cancer genome atlas (TCGA) dataset (https://portal.gdc.cancer.gov/). Differential expression of mRNA was analyzed using “Limma” [21] package of R project. Adjusted *P* values were analyzed in TCGA or GTEx to correct false positive results. Adjusted *P* < 0.05 and log_2_ (Fold Change) >1 was defined as the threshold for the screening of mRNA differential expression and the mapping of volcano plot.

### 2.3. Construct Protein-Protein Interactions and Drug-Target-Disease Networks

The potential targets of LUSC, differential expressed genes, and predicted targets of CEP were intersected to obtain common targets of drugs and diseases, and a Venn diagram was drawn. A protein-protein interaction (PPI) network of common targets was constructed using STRING 11.5 (https://string-db.org/) database with a minimum interaction score of 0.4. Cytoscape [22] software was used for visualization. In addition, cytohubba plugin was used to screen key genes.

### 2.4. Functional Enrichment Analysis

Gene ontology (GO) is a widely used tool for annotating functional genes, particularly molecular functions (MF), biological pathways (BP), and cellular components (CC). KEGG enrichment analysis is useful for analyzing gene function and related high-level genomic function information. ClusterProfiler [23] package in R project was used to analyze the GO function and KEGG pathway involved in potential targets.

### 2.5. Molecular Docking Analysis

Molecular docking is a validation method that uses a computer to simulate the binding of receptors and ligands and predict their affinity. PDB format files of target proteins were obtained from RCSB PDB database (https://www.rcsb.org/), and AutoDock Tools [24] were used for dehydrating, hydrogenation, and charge treatment of target proteins. The file in mol2 format was downloaded from TCMSP [18] database for structural processing. AutoDock Vina [25] was used for molecular docking to calculate binding energy and the best binding conformation. The docking results were visualized using PyMOL (The PyMOL Molecular Graphics System, Version 2.0 Schrodinger, LLC).

### 2.6. Analysis of Expression and Survival

Using the RNA-seq data of LUSC in TCGA, R project was used to analyze the differential expression of target genes in normal tissues and tumor tissues, the statistical difference of two groups was compared through the Wilcox test. We obtained the gene expression matrix of LUSC cell line from the CCLE database (https://portals.broadinstitute.org/ccle/about) and visualized it using heat maps. We retrieved the target genes in The Human Protein Atlas database (https://www.proteinatlas.org/), and immunohistochemical images were used to directly compare the protein expression of target genes in LUSC and normal lung tissues. Log-rank test was used to compare differences in survival between these groups. Kaplan-Meier curves, *P* values, and hazard ratio (HR) with 95% confidence interval (CI) were generated by log-rank tests and univariate cox proportional hazards regression. *P* < 0.05 was considered statistically significant.

### 2.7. The Stemness Index Based on mRNA Expression

We used the one-class logistic regression (OCLR) machine learning algorithm to calculate mRNAsi which constructed by Malta et al. [26]. Based on the mRNA expression signature, the gene expression profile contains 11,774 genes. The minimum value was subtracted, and the result was divided by the maximum maps the stemness index to the range [0,1]. The closer mRNAsi was to 1, the stronger was the characteristics of stem cells.

### 2.8. Tumor Immune Cells Infiltration and Tumor Mutation Burden

For reliable immune score evaluation, RNA-seq data of LUSC from TCGA database were obtained, and the correlation between target genes and immune cell infiltration was analyzed by TIMER [27] algorithm. Wilcoxon rank-sum test was used to accurately assess the differences in the expression levels of different target genes and the density of immune cell invasion in LUSC. The correlations between target expressions and immune or molecular subtypes of LUSC were explored via the TISIDB [28] database (http://cis.hku.hk/TISIDB/index.php). Differences with a *P* value < 0.05 were considered to be statistically significant. Correlation analysis between target genes' expressions and TMB was performed using Spearman's method. The analysis results were visualized by R package ggplot2 and PheatMap.

## 3. Results

### 3.1. Common Targets of CEP and LUSC

**Figure 1:**
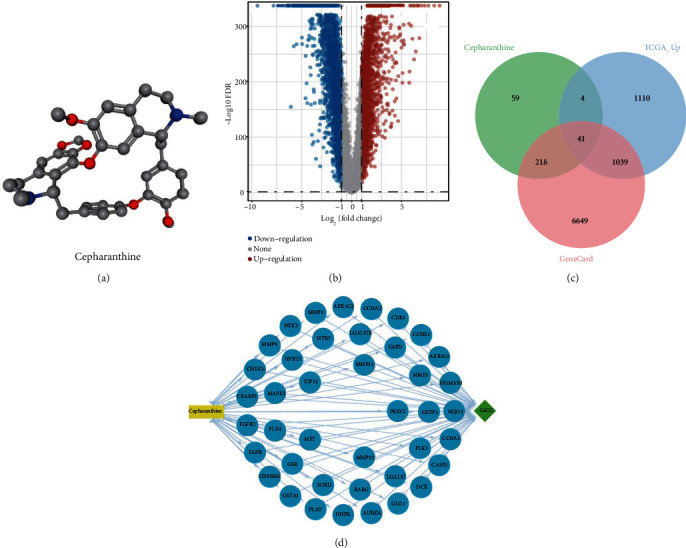
Common targets of CEP and LUSC. (a) 3D structure of CEP. (b) Volcano plot of differentially expressed genes. (c) Venn diagram of intersecting targets. (d) drug-target-disease network.

Through PubChem database, we obtained the 3D structure of CEP (Figure 1(a)) and predicted 231 and 121 CEP-related target proteins by PharmMapper and SwissTargetPrediction databases, respectively. After removing repeated targets, a total of 322 related target genes of CEP were collected (Table S1). 7947 LUSC-related target genes were retrieved from the GeneCards database (Table S2). In addition, RNA-seq data of 501 LUSC samples and 49 paracancer samples from TCGA database and sequencing data of 578 normal lung tissue samples from GTEx database were downloaded to obtain 2194 upregulated genes in tumor tissues by difference analysis (Figure 1(b)). Finally, through the intersection of the three gene sets, 41 potential targets for the regulation of LUSC progression were obtained (Figure 1(c)), and a drug-target-disease network was drawn (Figure 1(d)).

### 3.2. Interaction and Enrichment Analysis of Target Proteins

**Figure 2:**
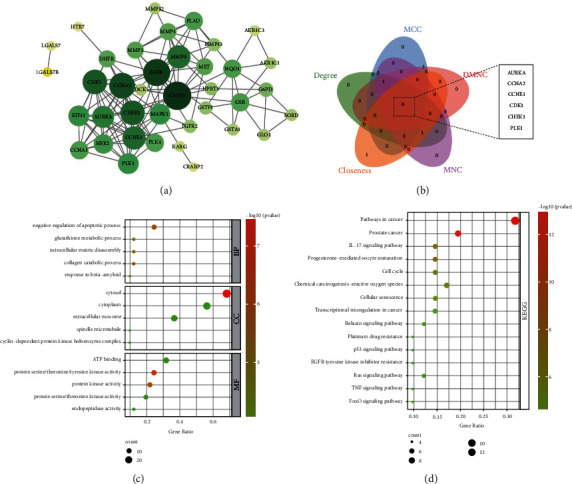
Interaction and enrichment analysis of target proteins. (a) The visualizing interaction network of target proteins. (b) Topological screening of hub genes. (c) GO enrichment analysis. (d) KEGG pathway enrichment analysis.

In order to study the correlation between CEP and LUSC, a 41 nodes and 130 edges protein-protein interaction (PPI) network was constructed using String database (Figure 2(a)). To further define key target proteins in PPI network, the MCC, Degree, DMNC, MNC, and Closeness algorithms in CytoHubba plugin are used to calculate node topology parameters (Table S3), and the top 10 targets are selected to intersect. Finally, we get six key target proteins: AURKA, CCNA2, CCNE1, CDK1, CHEK1, and PLK1 (Figure 2(b)). In addition, we also conducted functional enrichment analysis of the GO and KEGG pathways involved in these targets using the R package “ClusterProfile”. GO enrichment analysis consisted of three items, biological process (BP), cellular composition (CC), and molecular function (MF). Biological processes mainly involve negative regulation of apoptotic process, collagen catabolic process, extracellular matrix disassembly, etc. Cellular composition mainly involves cytosol, extracellular exosome, cyclin-dependent protein kinase holoenzyme complex etc. And molecular function mainly involves protein serine/threonine/tyrosine kinase activity, protein kinase activity, endopeptidase activity, etc. (Figure 2(c)). The KEGG pathways involved mainly include: pathways in cancer, IL-17 signaling pathway, progesterone-mediated oocyte maturation, cell cycle, cellular senescence, p53 signaling pathway, etc. (Figure 2(d)).

### 3.3. Molecular Docking

**Figure 3:**
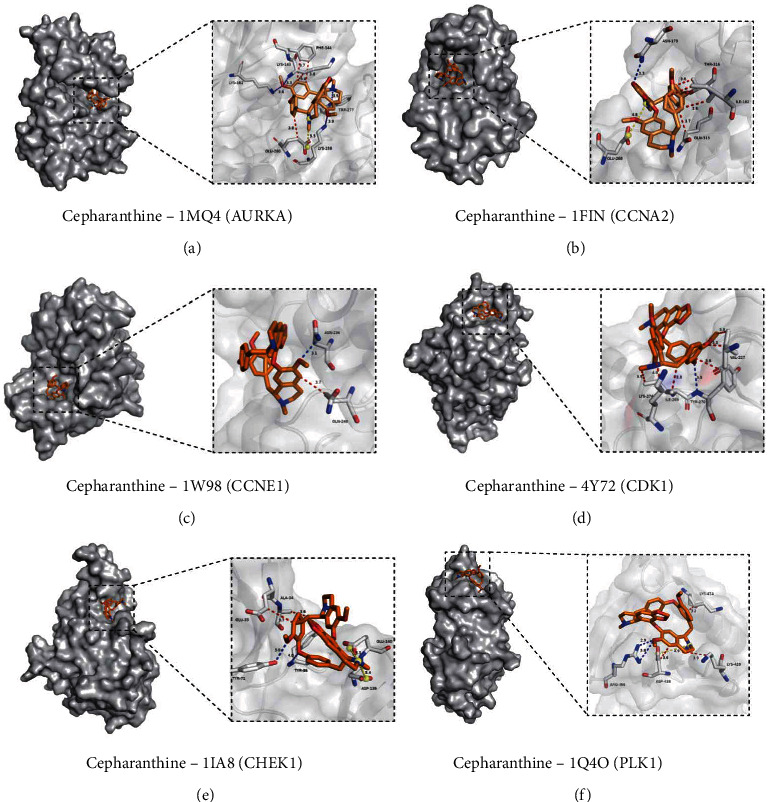
Molecular docking. (a) Docking of CEP and AURKA. (b) Docking of CEP and CCNA2. (c) Docking of CEP and CCNE1. (d) Docking of CEP and CDK1. (e) Docking of CEP and CHEK1. (f) Docking of CEP and PLK1. Dashed blue lines represent hydrogen bonds, dashed red lines represent hydrophobic interactions, and dashed red lines represent salt bridge.

After identifying the 6 key target proteins, we further verified the interaction between CEP and these target proteins by molecular docking. As shown in Table 1, the lowest binding affinity of CEP with AURKA, CCNA2, CCNE1, CDK1, CHEK1, and PLK1 were -9.1 kcal/mol, -8.4 kcal/mol, -9.0 kcal/mol, -9.2 kcal/mol, -7.6 kcal/mol, and -8.5 kcal/mol, respectively. These all show a strong binding affinity. As shown in Figure 3, the binding of CEP with 1mq4 (AURKA) is mainly through the hydrophobic interaction with LYS-143, PHE-144, and GLU-260; hydrogen bonding with amino acid residues LYS-143, LYS-162, LYS-258, and TRP-277; salt bridge with GLU-260. The binding of CEP with 1fin (CCNA2) is mainly through the hydrophobic interaction with ILE-182, GLN-313, and THR-316; hydrogen bonding with amino acid residues ASN-173; salt bridge with GLU-268. The binding of CEP with 1w98 (CCNE1) is mainly through the hydrophobic interaction with GLN-240 and hydrogen bonding with amino acid residues ASN-236. The binding of CEP with 4y72 (CDK1) is mainly through the hydrophobic interaction with VAL-227, ILE-269, TYR-71, and LYS-274 and hydrogen bonding with amino acid residues TYR-270. The binding of CEP with 1ia8 (CHEK1) is mainly through the hydrophobic interaction with GLU-33 and ALA-34; hydrogen bonding with amino acid residues TYR-71 and TYR-86; salt bridge with ASP-139 and GLU-140. The binding of CEP with 1q4o (PLK4) is mainly through the hydrophobic interaction with LYS-420, ASP-438, and LYS-474; hydrogen bonding with amino acid residues ARG-456; salt bridge with ASP-438 (Table S3).

### 3.4. Expression Levels of these Targets

**Figure 4:**
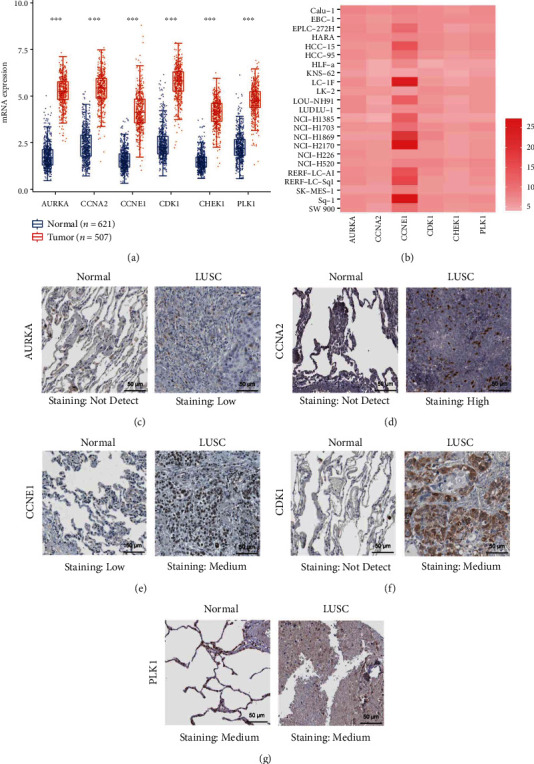
Expression of these targets. (a) Transcriptional expression of targets from TCGA. (b) Expressed differently of targets in various cell lines. (c–g) Protein expression of targets in HPA.

**Figure 5:**
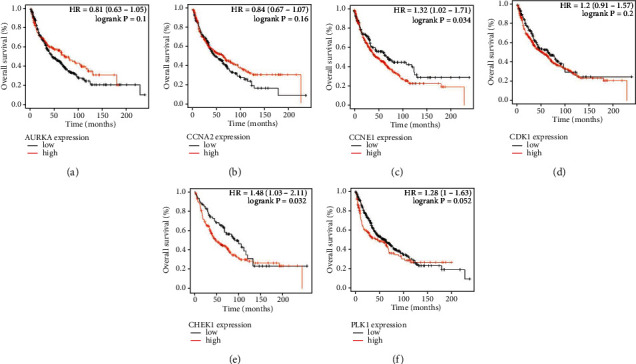
Overall survival. Comparison of overall survival between the different expression level of AURKA (a), CCNA2 (b), CCNE1 (c), CDK1 (d), CHEK1 (e), and PLK1 (f).

To further explore the mechanism of CEP in LUSC, we performed bioinformatics analysis on these 6 key targets. First, we analyzed TCGA transcriptional data and found that they were significantly upregulated in LUSC tissue compared to normal lung tissue (all *P* < 0.05) (Figure 4(a)). In the meanwhile, we analyzed the expression data of cell lines in the CCLE dataset and found that these 6 genes were also expressed differently in various cell lines of LUSC (Figure 4(b)). In addition, we analyzed the HPA database and found that compared with normal lung tissues, the protein expression levels of AURKA, CCNA2, CCNE1, CDK1, and PLK1 were significantly increased in LUSC tissues (Figure 4(c)–4(g)). The expression of CHEK1 protein has not been recorded in HPA database, but in Grabauskiene et al.'s study, CHEK1 protein was upregulated in LUSC [29]. In the analysis of the expressions of these targets and survival, the high expression of CCNA2 and CHEK1 was associated with shorter overall survival in LUSC patients (*P* < 0.05), while the other gene expression levels were not significantly associated with overall survival in LUSC patients (*P* > 0.05) (Figure 5).

### 3.5. Evaluation of Stemness Index

**Figure 6:**
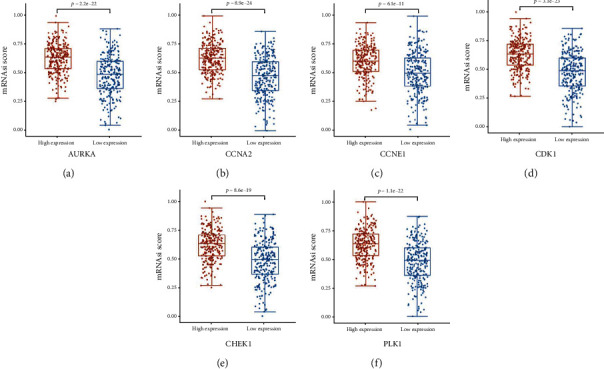
Cancer stemness index. Analysis of mRNAsi differences in samples with different expression levels of AURKA (a), CCNA2 (b), CCNE1 (c), CDK1 (d), CHEK1 (e), and PLK1 (f).

To evaluate the possible effect of CEP on cancer stemness, we divided LUSC samples from TCGA into a high-expression group and low-expression group according to the mRNA expression median value of target genes. OCLR machine learning algorithm was used to calculate stemness index (mRNAsi) to analyze the stemness degree among samples with differential expression of CEP targets. The results showed that the mRNAsi in the samples with high mRNA expression of AURKA, CCNA2, CCNE1, CDK1, CHEK1, and PLK1 was significantly higher than that in the samples with low mRNA expression (Figure 6).

### 3.6. Immune Cell Infiltration Analysis of Targets

**Figure 7:**
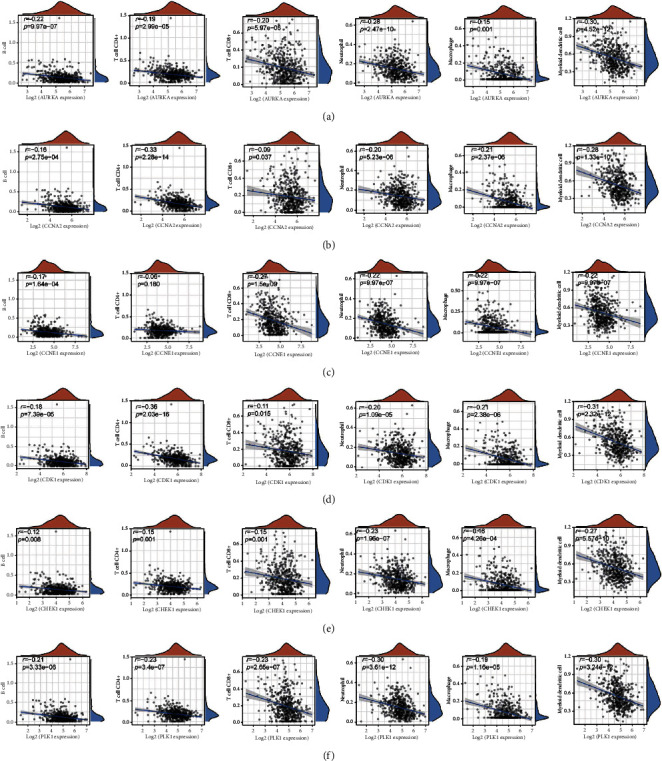
Immune cell infiltration. Correlation between expression levels of AURKA (a), CCNA2 (b), CCNE1 (c), CDK1 (d), CHEK1 (e), and PLK1 (f) and immune cell infiltration.

**Figure 8:**
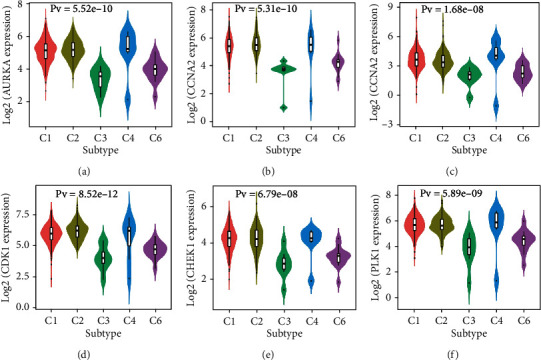
Immune subtypes of LUSC. The relationship between expressions of AURKA (a), CCNA2 (b), CCNE1 (c), CDK1 (d), CHEK1 (e), and PLK1 (f) and immune subtypes. C1, wound healing. C2, IFN-gamma dominant. C3, inflammatory. C4, lymphocyte depleted. C5, immunologically quiet. C6, TGF-b dominant.

Cancer stem cells have been proven to have immunosuppressive effects, and previous studies have shown that CEP has immunomodulatory effects. Therefore, we analyzed the relationship between expression of CEP targets and level of immune cell infiltration in LUSC. By calculating levels of immune cell infiltration for six types, the expression of AURKA were negatively correlated with infiltration levels of B cell, CD4+ T cell, CD8+ T cell, neutrophil, macrophage, and myeloid dendritic cell. Similarly, the expression of other target genes is also negatively correlated with the level of immune cell infiltration (Figure 7). Further, we analyzed the relationship between immune subtypes and expression of targets, and the immune subtypes were classified into six types, including C1 (wound healing), C2 (IFN-gamma dominant), C3 (inflammatory), C4 (lymphocyte depleted), C5 (immunologically quiet), and C6 (TGF-b dominant). The results showed that AURKA was lowly expressed in inflammatory samples, while AURKA was highly expressed in lymphocyte depleted samples. The correlation between immune subtypes and expression of the other five targets showed similar results (Figure 8).

### 3.7. Tumor Mutation Burden Analysis

**Figure 9:**
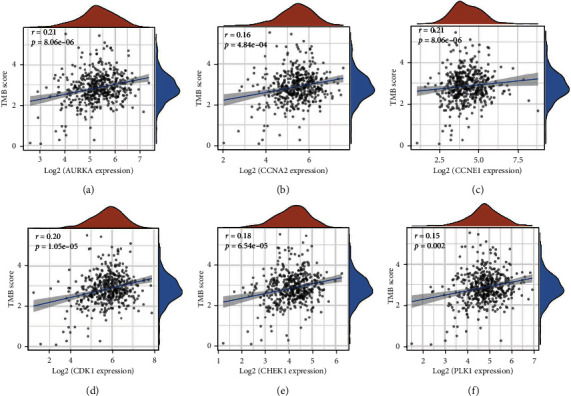
Tumor mutation burden. The relationship between expressions of AURKA (a), CCNA2 (b), CCNE1 (c), CDK1 (d), CHEK1 (e), and PLK1 (f) and tumor mutation burden.

Antitumor immunity requires T cells to recognize neoantigens caused by somatic mutations. Therefore, we analyzed the correlation between tumor mutation burden and the expression of targets, and the results showed that the expression of AURKA, CCNA2, CCNE1, CDK1, CHEK1, and PLK1 were significantly positively correlated with the tumor mutation burden (Figure 9).

## 4. Discussion

Network pharmacology can explore the complex mechanisms among biological systems, diseases, and drugs from the perspective of network and become an effective method for drug discovery [30]. In addition, molecular docking can accelerate drug design and screening by predicting the affinity and binding pattern between drugs and proteins and provide a basic theory for future experiments [31]. In this study, a reverse approach based on molecular docking was used to predict CEP targets and integrate them with LUSC targets. A total of 41 candidate targets were identified. The PPI network analysis revealed that CEP may have pharmacological effects on LUSC through 41 candidate targets.

According to the biological processes in GO analysis, 41 candidate targets were found to be related to negative regulation of apoptotic process. Previous studies have shown that CEP can induce apoptosis of myeloma cells by activating caspase-3 pathway [32]. It has also been confirmed that CEP can inhibit the expression of STAT3 gene and lead to the apoptosis of SaOS2 cells [33]. In addition, KEGG analysis showed that the targets of CEP were enriched in pathways in cancer, IL-17 signaling Pathway, Cell cycle, Cellular senescence, and p53 signaling pathway. Previous studies have demonstrated the role of CEP in the treatment of a variety of tumors [34–36], as well as its inhibitory effect on lung cancer cells [37]. However, the mechanism of CEP in treating LUSC has not been reported.

In order to clarify the possible mechanism of CEP in the treatment of LUSC, we conducted further research from the perspective of bioinformatics. Six hub genes were identified as AURKA, CCNA2, CCNE1, CDK1, CHEK1, and PLK1 by topological analysis of the 41 targets PPI network. Meanwhile, molecular docking analysis showed that CEP could stably bind these targets.

After determining that CEP can interact with these targets, we further analyzed the role of these targets in LUSC. First of all, we found that the stemness index (mRNAsi) was significantly higher in the samples with high expression of 6 targets than in the samples with low expression. Stemness refers to the cell's self-renewal and differentiation potential [26]. In tumor tissue, there is a group of cells with stem cell-like characteristics called cancer stem cells (CSCs) [38]. mRNAsi index is a new dryness index used to evaluate the dedifferentiation potential of tumor cells. The higher the stemness index, the higher the ability of cancer cells to dedifferentiate [26, 39]. These undifferentiated cancer cells are more likely to metastasize, leading to disease progression and poor prognosis. It also has a significant impact on treatment resistance and immunotherapy response [40]. Therefore, we hypothesized that CEP may inhibit LUSC progression by reducing tumor stemness by acting on these targets.

In addition, we found that the expressions of AURKA, CCNA2, CCNE1, CDK1, CHEK1, and PLK1 were all positively correlated with TMB, which was consistent with previous research results, and there was a positive correlation between tumor dryness and tumor mutation burden [39]. It is also consistent with studies demonstrating accumulation of mutations in normal adult stem cells [41].

In terms of immune microenvironment, we found that the expressions of AURKA, CCNA2, CCNE1, CDK1, CHEK1, and PLK1 were negatively correlated with tumor immune infiltrating cells. Meanwhile, we also found that the expression of targets in immune inflammatory tumors was low, and the samples with high expression of targets were mainly distributed in lymphocytes depleted type tumors. Previous studies have shown that in colorectal cancer, lung cancer, and ovarian cancer, tumor stemness is negatively correlated with immune cell infiltration, and the higher the stemness of the tumor, the lower the T cell infiltrating level [39].

There are some limitations to this study. We have only explored the role of CEP in LUSC at the level of network pharmacology and bioinformatics, and further studies in experiments of molecular biology are needed. Secondly, network pharmacology studies rely more on various existing databases, but the screening criteria for drugs in the databases may not be accurate enough.

## 5. Conclusions

Our study systematically explored the molecular and pharmacological mechanisms of CEP on LUSC. Six genes were identified as anticancer targets of CEP that reduce LUSC stem cell characteristics and increase immune cell infiltration. This study provides new ideas for treating LUSC and lays a foundation for drug development.

## Figures and Tables

**Table 1:** The result of the lowest binding affinity of molecular docking.

Target name	Uniprot ID	PDB ID	Binding affinity (kcal/mol)
AURKA	O14965	1mq4	-9.1
CCNA2	P20248	1fin	-8.4
CCNE1	P24864	1w98	-9.0
CDK1	P06493	4y72	-9.2
CHEK1	O14757	1ia8	-7.6
PLK1	P53350	1q4o	-8.5

## Data Availability

The data used to support the findings of this study are available from TCGA (https://portal.gdc.cancer.gov/), RCSB PDB database (https://www.rcsb.org/), GeneCards database (https://www.genecards.org/), and TISIDB (http://cis.hku.hk/TISIDB/index.php).

## Supplementary Materials

Supplementary 1 Table S1 The related targets of cepharanthine.

Supplementary 2 Table S2 Lung squamous cell carcinoma-related genes gained from GeneCards database.

Supplementary 3 Table S3 Topological parameters of network nodes.

Supplementary 4 Table S4 the binding sites of CEP at the target protein.
