# Supplementary material for: Effect of Cepharanthine on the Stemness of Lung Squamous Cell Carcinoma Based on Network Pharmacology and Bioinformatics
Source: Biomed Res Int. 2022 Nov 28;2022:5956526. doi: 10.1155/2022/5956526 (PMC9723418; doi:10.1155/2022/5956526)
Supplement: Supplementary 1 — Table S1 The related targets of cepharanthine. [file 5956526.f1.docx]

**Table S1** The related targets of cepharanthine.

| **Pharma Model** | **Name** | **Uniplot** |
| --- | --- | --- |
| 1pq9_v | Oxysterols receptor LXR-beta | NR1H2_HUMAN |
| 1eub_v | Collagenase 3 | MMP13_HUMAN |
| 1w8m_v | Peptidyl-prolyl cis-trans isomerase A | P62937 |
| 1v3q_v | Purine nucleoside phosphorylase | PNPH_HUMAN |
| 1ogs_v | Glucosylceramidase | GLCM_HUMAN |
| 3bgp_v | Proto-oncogene serine/threonine-protein kinase Pim-1 | P11309 |
| 1pmv_v | Mitogen-activated protein kinase 10 | MK10_HUMAN |
| 1xlv_v | Cholinesterase | CHLE_HUMAN |
| 2h96_v | Mitogen-activated protein kinase 8 | MK08_HUMAN |
| 1e5a_v | Transthyretin | TTHY_HUMAN |
| 1dic_v | Complement factor D | CFAD_HUMAN |
| 1xan_v | Glutathione reductase, mitochondrial | P00390 |
| 1o4i_v | Proto-oncogene tyrosine-protein kinase Src | SRC_HUMAN |
| 1mkd_v | cAMP-specific 3,5-cyclic phosphodiesterase 4D | PDE4D_HUMAN |
| 1n1m_v | Dipeptidyl peptidase 4 | DPP4_HUMAN |
| 1rkp_v | cGMP-specific 3,5-cyclic phosphodiesterase | PDE5A_HUMAN |
| 1oec_v | Fibroblast growth factor receptor 2 | P21802 |
| 5gal_v | Galectin-7 | LEG7_HUMAN |
| 1lhv_v | Sex hormone-binding globulin | SHBG_HUMAN |
| 1csb_v | Cathepsin B | CATB_HUMAN |
| 2ohq_v | Beta-secretase 1 | BACE1_HUMAN |
| 1kwq_v | Carbonic anhydrase 2 | CAH2_HUMAN |
| 2zkc_v | Estrogen-related receptor gamma | P62508 |
| 1ghx_v | Prothrombin | THRB_HUMAN |
| 1hnn_v | Phenylethanolamine N-methyltransferase | PNMT_HUMAN |
| 2v5z_v | Amine oxidase [flavin-containing] B | AOFB_HUMAN |
| 2fb8_v | B-Raf proto-oncogene serine/threonine-protein kinase | BRAF1_HUMAN |
| 1hak_v | Annexin A5 | ANXA5_HUMAN |
| 1cvw_v | Coagulation factor VII | FA7_HUMAN |
| 1kbo_v | NAD(P)H dehydrogenase [quinone] 1 | NQO1_HUMAN |
| 2bok_v | Coagulation factor X | FA10_HUMAN |
| 1rw8_v | TGF-beta receptor type-1 | TGFR1_HUMAN |
| 3f68_v | NONE | NONE |
| 1pl6_v | Sorbitol dehydrogenase | DHSO_HUMAN |
| 2bx8_v | Serum albumin | ALBU_HUMAN |
| 1az1_v | Aldose reductase | ALDR_HUMAN |
| 1di9_v | Mitogen-activated protein kinase 14 | Q16539 |
| 1xlz_v | cAMP-specific 3,5-cyclic phosphodiesterase 4B | PDE4B_HUMAN |
| 2itu_v | Epidermal growth factor receptor | EGFR_HUMAN |
| 1xbc_v | Tyrosine-protein kinase SYK | KSYK_HUMAN |
| 1ld8_v | Protein farnesyltransferase/geranylgeranyltransferase type-1 subunit alpha | FNTA_HUMAN |
| 2fs4_v | Renin | RENI_HUMAN |
| 1owk_v | Urokinase-type plasminogen activator | UROK_HUMAN |
| 2acl_v | Retinoic acid receptor RXR-alpha | RXRA_HUMAN |
| 1so2_v | cGMP-inhibited 3,5-cyclic phosphodiesterase B | PDE3B_HUMAN |
| 2of4_v | Proto-oncogene tyrosine-protein kinase LCK | LCK_HUMAN |
| 1t48_v | Tyrosine-protein phosphatase non-receptor type 1 | PTN1_HUMAN |
| 1pxk_v | Cell division protein kinase 2 | P24941 |
| 1rqd_v | Deoxyhypusine synthase | DHYS_HUMAN |
| 1a8j_v | Ig lambda chain V-II region MGC | P01709 |
| 1cgh_v | Cathepsin G | CATG_HUMAN |
| 1ctr_v | Calmodulin | CALM_HUMAN |
| 1gz4_v | NAD-dependent malic enzyme, mitochondrial | MAOM_HUMAN |
| 1m9r_v | Nitric oxide synthase, endothelial | NOS3_HUMAN |
| 1ya4_v | Liver carboxylesterase 1 | EST1_HUMAN |
| 1k59_v | Angiogenin | ANGI_HUMAN |
| 1stc_v | cAMP-dependent protein kinase catalytic subunit alpha | P00517 |
| 2rl5_v | Vascular endothelial growth factor receptor 2 | P35968 |
| 1qyx_v | Estradiol 17-beta-dehydrogenase 1 | P14061 |
| 1jj9_v | Neutrophil collagenase | MMP8_HUMAN |
| 1uk0_v | Poly [ADP-ribose] polymerase 1 | P09874 |
| 2ovh_v | Progesterone receptor | PRGR_HUMAN |
| 1rd4_v | Integrin alpha-L | ITAL_HUMAN |
| 1vj5_v | Epoxide hydrolase 2 | HYES_HUMAN |
| 1rv1_v | E3 ubiquitin-protein ligase Mdm2 | MDM2_HUMAN |
| 1d8m_v | Stromelysin-1 | MMP3_HUMAN |
| 2fky_v | Kinesin-like protein KIF11 | KIF11_HUMAN |
| 1mrq_v | Aldo-keto reductase family 1 member C1 | Q04828 |
| 1yvj_v | Tyrosine-protein kinase JAK3 | JAK3_HUMAN |
| 1i7i_v | Peroxisome proliferator-activated receptor gamma | PPARG_HUMAN |
| 2bkz_v | Cyclin-A2 | CCNA2_HUMAN |
| 1h1b_v | Leukocyte elastase | ELNE_HUMAN |
| 1t67_v | Histone deacetylase 8 | HDAC8_HUMAN |
| 1jqh_v | Insulin-like growth factor 1 receptor | IGF1R_HUMAN |
| 1shl_v | Caspase-7 | CASP7_HUMAN |
| 2w1g_v | Serine/threonine-protein kinase 6 | O14965 |
| 1t84_v | Wiskott-Aldrich syndrome protein | WASP_HUMAN |
| 2oi0_v | ADAM 17 | ADA17_HUMAN |
| 1s1p_v | Aldo-keto reductase family 1 member C3 | AK1C3_HUMAN |
| 1e8z_v | Phosphatidylinositol-4,5-bisphosphate 3-kinase catalytic subunit gamma isoform | PK3CG_HUMAN |
| 3ekr_v | Heat shock protein HSP 90-alpha | P07900 |
| 3f7h_v | Baculoviral IAP repeat-containing protein 7 | Q96CA5 |
| 1r7t_v | Histo-blood group ABO system transferase | P16442 |
| 1uu8_v | 3-phosphoinositide-dependent protein kinase 1 | PDPK1_HUMAN |
| 1r0p_v | Hepatocyte growth factor receptor | MET_HUMAN |
| 1w7n_v | Kynurenine--oxoglutarate transaminase 1 | KAT1_HUMAN |
| 1dhf_v | Dihydrofolate reductase | DYR_HUMAN |
| 2i6b_v | Adenosine kinase | P55263 |
| 2bu5_v | [Pyruvate dehydrogenase [lipoamide]] kinase isozyme 2, mitochondrial | PDK2_HUMAN |
| 1x6v_v | Bifunctional 3-phosphoadenosine 5-phosphosulfate synthetase 1 | O43252 |
| 1yk7_v | Cathepsin K | CATK_HUMAN |
| 1n69_v | Proactivator polypeptide | P07602 |
| 1og5_v | Cytochrome P450 2C9 | CP2C9_HUMAN |
| 1ov4_v | Bile salt sulfotransferase | Q06520 |
| 1wda_v | Protein-arginine deiminase type-4 | PADI4_HUMAN |
| 1he3_v | Flavin reductase | P30043 |
| 1q3w_v | Glycogen synthase kinase-3 beta | GSK3B_HUMAN |
| 1nd5_v | Prostatic acid phosphatase | PPAP_HUMAN |
| 1sj0_v | Estrogen receptor | ESR1_HUMAN |
| 2e9u_v | Serine/threonine-protein kinase Chk1 | CHK1_HUMAN |
| 1uhl_v | Oxysterols receptor LXR-alpha | NR1H3_HUMAN |
| 1tou_v | Fatty acid-binding protein, adipocyte | FABP4_HUMAN |
| 2aa5_v | Mineralocorticoid receptor | MCR_HUMAN |
| 1u59_v | Tyrosine-protein kinase ZAP-70 | ZAP70_HUMAN |
| 2fhi_v | Bis(5-adenosyl)-triphosphatase | P49789 |
| 1fkg_v | Peptidyl-prolyl cis-trans isomerase FKBP1A | FKB1A_HUMAN |
| 1utz_v | Macrophage metalloelastase | MMP12_HUMAN |
| 1sm2_v | Tyrosine-protein kinase ITK/TSK | Q08881 |
| 1xjd_v | Protein kinase C theta type | KPCT_HUMAN |
| 1a5h_v | Tissue-type plasminogen activator | TPA_HUMAN |
| 1nxk_v | MAP kinase-activated protein kinase 2 | P49137 |
| 1nav_v | Thyroid hormone receptor alpha | P10827 |
| 1i7g_v | Peroxisome proliferator-activated receptor alpha | PPARA_HUMAN |
| 1gzr_v | Insulin-like growth factor IA | P01343 |
| 2oaz_v | Methionine aminopeptidase 2 | AMPM2_HUMAN |
| 1xoi_v | Glycogen phosphorylase, liver form | P06737 |
| 1h6g_v | Catenin alpha-1 | CTNA1_HUMAN |
| 1db5_v | Phospholipase A2, membrane associated | P14555 |
| 1xq3_v | Androgen receptor | ANDR_HUMAN |
| 1qab_v | Retinol-binding protein 4 | RET4_HUMAN |
| 1mq0_v | Cytidine deaminase | CDD_HUMAN |
| 3cm7_v | Baculoviral IAP repeat-containing protein 4 | P98170 |
| 1sd2_v | S-methyl-5-thioadenosine phosphorylase | Q13126 |
| 1bj4_v | Serine hydroxymethyltransferase, cytosolic | GLYC_HUMAN |
| 3bbt_v | Receptor tyrosine-protein kinase erbB-4 | ERBB4_HUMAN |
| 1hw9_v | 3-hydroxy-3-methylglutaryl-coenzyme A reductase | HMDH_HUMAN |
| 1hms_v | Fatty acid-binding protein, heart | FABPH_HUMAN |
| 1x89_v | Neutrophil gelatinase-associated lipocalin | NGAL_HUMAN |
| 3d7t_v | Tyrosine-protein kinase CSK | CSK_HUMAN |
| 1l2j_v | Estrogen receptor beta | ESR2_HUMAN |
| 1fe3_v | Fatty acid-binding protein, brain | FABP7_HUMAN |
| 1o1v_v | Gastrotropin | FABP6_HUMAN |
| 1ilh_v | Nuclear receptor subfamily 1 group I member 2 | NR1I2_HUMAN |
| 1nmx_v | Thymidylate kinase | KTHY_HUMAN |
| 1jqe_v | Histamine N-methyltransferase | P50135 |
| 1svh_v | cAMP-dependent protein kinase, alpha-catalytic subunit | P00517 |
| 1h0c_v | Serine--pyruvate aminotransferase | SPYA_HUMAN |
| 1uzf_v | Angiotensin-converting enzyme | ACE_HUMAN |
| 13gs_v | Glutathione S-transferase P | GSTP1_HUMAN |
| 1pw6_v | Interleukin-2 | IL2_HUMAN |
| 1yvl_v | Signal transducer and activator of transcription 1-alpha/beta | STAT1_HUMAN |
| 2fgi_v | Basic fibroblast growth factor receptor 1 | FGFR1_HUMAN |
| 1nhx_v | Phosphoenolpyruvate carboxykinase, cytosolic [GTP] | P35558 |
| 1hki_v | Chitotriosidase-1 | Q13231 |
| 1isj_v | ADP-ribosyl cyclase 2 | BST1_HUMAN |
| 2vd1_v | Glutathione-requiring prostaglandin D synthase | PTGD2_HUMAN |
| 1qcf_v | Tyrosine-protein kinase HCK | HCK_HUMAN |
| 1p62_v | Deoxycytidine kinase | DCK_HUMAN |
| 1yq7_v | Farnesyl pyrophosphate synthetase | FPPS_HUMAN |
| 1lt8_v | Betaine--homocysteine S-methyltransferase 1 | Q93088 |
| 1p5j_v | L-serine dehydratase | P20132 |
| 1gzu_v | Nicotinamide mononucleotide adenylyltransferase 1 | Q9HAN9 |
| 1itu_v | Dipeptidase 1 | DPEP1_HUMAN |
| 1t46_v | Mast/stem cell growth factor receptor | KIT_HUMAN |
| 1s9j_v | Dual specificity mitogen-activated protein kinase kinase 1 | MP2K1_HUMAN |
| 1nhz_v | Glucocorticoid receptor | GCR_HUMAN |
| 2ojj_v | Mitogen-activated protein kinase 1 | MK01_HUMAN |
| 1gkd_v | Matrix metalloproteinase-9 | MMP9_HUMAN |
| 1tfg_v | Transforming growth factor beta-2 | TGFB2_HUMAN |
| 1l9n_v | Protein-glutamine gamma-glutamyltransferase E | TGM3_HUMAN |
| 2fs9_v | Tryptase beta-2 | TRYB2_HUMAN |
| 1ekv_v | Branched-chain-amino-acid aminotransferase, mitochondrial | O15382 |
| 1fd0_v | Retinoic acid receptor gamma | RARG_HUMAN |
| 1x0n_v | Growth factor receptor-bound protein 2 | GRB2_HUMAN |
| 1ljr_v | Glutathione S-transferase theta-2 | GSTT2_HUMAN |
| 1gsf_v | Glutathione S-transferase A1 | P08263 |
| 1q4x_v | Thyroid hormone receptor beta | P10828 |
| 1g3m_v | Estrogen sulfotransferase | ST1E1_HUMAN |
| 1h9u_v | Retinoic acid receptor RXR-beta | RXRB_HUMAN |
| 1gw6_v | Leukotriene A-4 hydrolase | LKHA4_HUMAN |
| 1nqc_v | Cathepsin S | CATS_HUMAN |
| 2yxj_v | Bcl-2-like protein 1 | Q07817 |
| 1jbq_v | Cystathionine beta-synthase | P35520 |
| 1r6u_v | Tryptophanyl-tRNA synthetase, cytoplasmic | P23381 |
| 2p4i_v | Angiopoietin-1 receptor | TIE2_HUMAN |
| 1i00_v | Thymidylate synthase | TYSY_HUMAN |
| 2gpq_v | Eukaryotic translation initiation factor 4E | P06730 |
| 3fxv_v | Bile acid receptor | Q96RI1 |
| 1zpb_v | Coagulation factor XI | FA11_HUMAN |
| 1sir_v | Glutaryl-CoA dehydrogenase, mitochondrial | Q92947 |
| 2b7a_v | Tyrosine-protein kinase JAK2 | JAK2_HUMAN |
| 1q5h_v | Deoxyuridine 5-triphosphate nucleotidohydrolase, mitochondrial | DUT_HUMAN |
| 1r1i_v | Neprilysin | NEP_HUMAN |
| 1xcx_v | Pancreatic alpha-amylase | AMYP_HUMAN |
| 1ice_v | Caspase-1 | CASP1_HUMAN |
| 1tdi_v | Glutathione S-transferase A3 | Q16772 |
| 1xw5_v | Glutathione S-transferase Mu 2 | GSTM2_HUMAN |
| 1iri_v | Glucose-6-phosphate isomerase | G6PI_HUMAN |
| 1kpf_v | Histidine triad nucleotide-binding protein 1 | HINT1_HUMAN |
| 1dkf_v | Retinoic acid receptor alpha | RARA_HUMAN |
| 1wms_v | Ras-related protein Rab-9 | P51151 |
| 1u4m_v | C-C motif chemokine 5 | CCL5_HUMAN |
| 2can_v | Ornithine aminotransferase, mitochondrial | OAT_HUMAN |
| 3cbs_v | Cellular retinoic acid-binding protein 2 | P29373 |
| 1oth_v | Ornithine carbamoyltransferase, mitochondrial | P00480 |
| 1rhj_v | Caspase-3 | CASP3_HUMAN |
| 1yj6_v | Glutathione S-transferase Mu 1 | GSTM1_HUMAN |
| 1q91_v | 5(3)-deoxyribonucleotidase, mitochondrial | Q9NPB1 |
| 1g1t_v | E-selectin | LYAM2_HUMAN |
| 1s0z_v | Vitamin D3 receptor | VDR_HUMAN |
| 1jk7_v | Serine/threonine-protein phosphatase PP1-gamma catalytic subunit | P36873 |
| 2fpy_v | Dihydroorotate dehydrogenase, mitochondrial | PYRD_HUMAN |
| 1qki_v | Glucose-6-phosphate 1-dehydrogenase | G6PD_HUMAN |
| 1liw_v | Pyruvate kinase isozymes R/L | KPYR_HUMAN |
| 2bel_v | Corticosteroid 11-beta-dehydrogenase isozyme 1 | DHI1_HUMAN |
| 2f4j_v | Proto-oncogene tyrosine-protein kinase ABL1 | P00519 |
| 1k6m_v | 6-phosphofructo-2-kinase/fructose-2,6-biphosphatase 1 | P07953 |
| 1xap_v | Retinoic acid receptor beta | RARB_HUMAN |
| 1g55_v | tRNA (cytosine-5-)-methyltransferase | O14717 |
| 1r55_v | ADAM 33 | ADA33_HUMAN |
| 1z6t_v | Apoptotic protease-activating factor 1 | APAF_HUMAN |
| 1t31_v | Chymase | CMA1_HUMAN |
| 1hi4_v | Non-secretory ribonuclease | P10153 |
| 1r5l_v | Alpha-tocopherol transfer protein | P49638 |
| 2auh_v | Insulin receptor | INSR_HUMAN |
| 1ln3_v | Phosphatidylcholine transfer protein | Q9UKL6 |
| 1cm0_v | Histone acetyltransferase PCAF | Q92831 |
| 1nus_v | Nicotinamide mononucleotide adenylyltransferase 3 | Q96T66 |
| 1q20_v | Sulfotransferase family cytosolic 2B member 1 | O00204 |
| 1xrj_v | Uridine-cytidine kinase 2 | Q9BZX2 |
| 1xmm_v | Scavenger mRNA-decapping enzyme DcpS | Q96C86 |
| 2c6q_v | GMP reductase 2 | Q9P2T1 |
| 1s0x_v | Nuclear receptor ROR-alpha | RORA_HUMAN |
| 1fro_v | Lactoylglutathione lyase | LGUL_HUMAN |
| 1b55_v | Tyrosine-protein kinase BTK | BTK_HUMAN |
| 1bzy_v | Hypoxanthine-guanine phosphoribosyltransferase | P00492 |
| 1jkl_v | Death-associated protein kinase 1 | DAPK1_HUMAN |
| 1l8j_v | Endothelial protein C receptor | EPCR_HUMAN |
| 1il0_v | Hydroxyacyl-coenzyme A dehydrogenase, mitochondrial | Q16836 |
| 1nb0_v | Riboflavin kinase | Q969G6 |
| 1pbk_v | FK506-binding protein 3 | FKBP3_HUMAN |
| 1yzg_v | ADP-ribosylation factor-like protein 5B | Q9D4P0 |
| 1ls6_v | Sulfotransferase 1A1 | ST1A1_HUMAN |
| 1unq_v | RAC-alpha serine/threonine-protein kinase | AKT1_HUMAN |
| 1dug_v | Fibrinogen gamma chain | FIBG_HUMAN |
| - | Dopamine D2 receptor (by homology) | P14416 |
| - | Dopamine transporter (by homology) | Q01959 |
| - | Multidrug and toxin extrusion protein 1 | Q96FL8 |
| - | Neuronal acetylcholine receptor; alpha4/beta2 | P43681 P17787 |
| - | Neuronal acetylcholine receptor; alpha3/beta4 | P32297 P30926 |
| - | Butyrylcholinesterase | P06276 |
| - | Neuronal acetylcholine receptor; alpha2/beta4 | P30926 Q15822 |
| - | Neuronal acetylcholine receptor; alpha3/beta2 | P32297 P17787 |
| - | Dopamine D1 receptor | P21728 |
| - | Muscarinic acetylcholine receptor M4 | P08173 |
| - | Phosphodiesterase 1A | P54750 |
| - | Serotonin 7 (5-HT7) receptor | P34969 |
| - | Serotonin 1a (5-HT1a) receptor | P08908 |
| - | Coagulation factor VII/tissue factor | P13726 |
| - | Dopamine D3 receptor | P35462 |
| - | Alpha-1a adrenergic receptor | P35348 |
| - | Sigma opioid receptor | Q99720 |
| - | Dopamine D5 receptor | P21918 |
| - | Dopamine D4 receptor | P21917 |
| - | Inhibitor of apoptosis protein 3 | P98170 |
| - | Baculoviral IAP repeat-containing protein 3 | Q13489 |
| - | Baculoviral IAP repeat-containing protein 2 | Q13490 |
| - | Protein kinase C gamma | P05129 |
| - | Protein kinase C alpha | P17252 |
| - | Protein kinase C beta | P05771 |
| - | Alpha-1b adrenergic receptor | P35368 |
| - | Serine/threonine-protein kinase PIM1 | P11309 |
| - | Serine/threonine-protein kinase PIM2 | Q9P1W9 |
| - | Hepatocyte growth factor receptor | P08581 |
| - | Thromboxane A2 receptor | P21731 |
| - | Gonadotropin-releasing hormone receptor | P30968 |
| - | Insulin-like growth factor I receptor | P08069 |
| - | 3-phosphoinositide dependent protein kinase-1 | O15530 |
| - | PI3-kinase p110-delta subunit | O00329 |
| - | PI3-kinase p110-beta subunit | P42338 |
| - | PI3-kinase p110-gamma subunit | P48736 |
| - | PI3-kinase p110-alpha subunit | P42336 |
| - | Epidermal growth factor receptor erbB1 | P00533 |
| - | Tyrosine-protein kinase TIE-2 | Q02763 |
| - | Beta-1 adrenergic receptor | P08588 |
| - | Adrenergic receptor beta | P07550 |
| - | Alpha-1d adrenergic receptor | P25100 |
| - | Vascular endothelial growth factor receptor 2 | P35968 |
| - | Cyclin-dependent kinase 1 | P06493 |
| - | Histone-lysine N-methyltransferase, H3 lysine-9 specific 5 | Q9H9B1 |
| - | Histone-lysine N-methyltransferase, H3 lysine-9 specific 3 | Q96KQ7 |
| - | Muscarinic acetylcholine receptor M1 | P11229 |
| - | Tyrosine-protein kinase LCK | P06239 |
| - | Cyclin-dependent kinase 2/cyclin E1 | P24864 P24941 |
| - | CDK9/cyclin T1 | P50750 O60563 |
| - | Tyrosine-protein kinase SRC | P12931 |
| - | Tyrosine-protein kinase HCK | P08631 |
| - | Tyrosine-protein kinase Lyn | P07948 |
| - | Tyrosine-protein kinase BTK | Q06187 |
| - | Serine/threonine-protein kinase 33 | Q9BYT3 |
| - | Serotonin 2a (5-HT2a) receptor | P28223 |
| - | Serine/threonine-protein kinase mTOR | P42345 |
| - | Protein kinase C delta | Q05655 |
| - | Protein kinase C (PKC) | Q05513 |
| - | Serine/threonine-protein kinase AKT | P31749 |
| - | Telomerase reverse transcriptase | O14746 |
| - | Serine/threonine-protein kinase PLK4 | O00444 |
| - | TGF-beta receptor type I | P36897 |
| - | Vasopressin V1b receptor | P47901 |
| - | C-C chemokine receptor type 4 | P51679 |
| - | Ileal bile acid transporter | Q12908 |
| - | Fatty acid synthase | P49327 |
| - | Methionine aminopeptidase 1 | P53582 |
| - | Ribosomal protein S6 kinase 1 | P23443 |
| - | Serine/threonine-protein kinase Aurora-A | O14965 |
| - | Protein kinase C theta | Q04759 |
| - | Hematopoietic prostaglandin D synthase | O60760 |
| - | Receptor-interacting serine/threonine-protein kinase 3 | Q9Y572 |
| - | Vascular endothelial growth factor receptor 1 | P17948 |
| - | Tyrosine-protein kinase receptor FLT3 | P36888 |
| - | Insulin receptor | P06213 |
| - | Platelet-derived growth factor receptor alpha | P16234 |
| - | NPM/ALK (Nucleophosmin/ALK tyrosine kinase receptor) | P06748 Q9UM73 |
| - | Matrix metalloproteinase 3 | P08254 |
| - | Matrix metalloproteinase 1 | P03956 |
| - | Nitric-oxide synthase, brain | P29475 |
| - | Fibroblast growth factor receptor 1 | P11362 |
| - | Bcl2-antagonist of cell death (BAD) | Q92934 |
| - | ALK tyrosine kinase receptor | Q9UM73 |
| - | Nitric oxide synthase, inducible | P35228 |
| - | Nitric-oxide synthase, endothelial | P29474 |
| - | Serine/threonine-protein kinase PIM3 | Q86V86 |
| - | Dual specificity tyrosine-phosphorylation-regulated kinase 1B | Q9Y463 |
| - | Macrophage colony stimulating factor receptor | P07333 |
| - | Platelet-derived growth factor receptor beta | P09619 |
| - | Cyclin-dependent kinase 2/cyclin A | P24941 P78396 P20248 |
| - | Protein kinase C iota | P41743 |
| - | MAP kinase p38 alpha | Q16539 |
| - | Renin | P00797 |
| - | Tyrosine-protein kinase JAK2 | O60674 |
| - | Cathepsin E | P14091 |
| - | Phosphodiesterase 11A | Q9HCR9 |
| - | Thrombin and coagulation factor X | P00742 |
| - | Cyclin-dependent kinase 2 | P24941 |
| - | Cyclin-dependent kinase 4 | P11802 |
