# Supplementary material for: Effect of Cepharanthine on the Stemness of Lung Squamous Cell Carcinoma Based on Network Pharmacology and Bioinformatics
Source: Biomed Res Int. 2022 Nov 28;2022:5956526. doi: 10.1155/2022/5956526 (PMC9723418; doi:10.1155/2022/5956526)
Supplement: Supplementary 2 — Table S2 Lung squamous cell carcinoma-related genes gained from GeneCards database. [file 5956526.f2.docx]

**Table S2** Lung squamous cell carcinoma-related genes gained from GeneCard database.

| Gene Symbol | GC ID |
| --- | --- |
| TP53 | GC17M007661 |
| EGFR | GC07P055019 |
| BRCA1 | GC17M043044 |
| PTEN | GC10P090708 |
| BRCA2 | GC13P032315 |
| KRAS | GC12M025204 |
| PIK3CA | GC03P179148 |
| ATM | GC11P108222 |
| MET | GC07P116672 |
| CDKN2A | GC09M021967 |
| CDH1 | GC16P068737 |
| ERBB2 | GC17P039687 |
| BRAF | GC07M140730 |
| MIR21 | GC17P059841 |
| AKT1 | GC14M104769 |
| CHEK2 | GC22M028687 |
| APC | GC05P112707 |
| MLH1 | GC03P036993 |
| CTNNB1 | GC03P041236 |
| MSH2 | GC02P047402 |
| RB1 | GC13P048303 |
| CASP8 | GC02P201233 |
| MIR34A | GC01M009151 |
| ALK | GC02M029190 |
| CCND1 | GC11P069641 |
| HRAS | GC11M002647 |
| MSH6 | GC02P047695 |
| STK11 | GC19P001177 |
| MIR205 | GC01P209432 |
| PALB2 | GC16M023603 |
| NBN | GC08M089933 |
| TERT | GC05M001253 |
| FASLG | GC01P172628 |
| MIR145 | GC05P149430 |
| KIT | GC04P054657 |
| FGFR3 | GC04P001795 |
| NRAS | GC01M114704 |
| MIR17 | GC13P091350 |
| MIR221 | GC0XM045746 |
| SMAD4 | GC18P051028 |
| MIR126 | GC09P136670 |
| DICER1 | GC14M095086 |
| PMS2 | GC07M005973 |
| MAP2K1 | GC15P066386 |
| MIR200C | GC12P018573 |
| BRIP1 | GC17M061679 |
| MIR222 | GC0XM045747 |
| RET | GC10P043121 |
| MIR141 | GC12P018574 |
| MYC | GC08P127735 |
| TGFBR2 | GC03P030623 |
| STAT3 | GC17M042313 |
| BCL2 | GC18M063123 |
| MIR200B | GC01P001167 |
| MIR20A | GC13P091533 |
| FGFR2 | GC10M121478 |
| IL6 | GC07P022725 |
| TGFB1 | GC19M041301 |
| BARD1 | GC02M214725 |
| MIR200A | GC01P003359 |
| CDKN1B | GC12P018790 |
| MIR125A | GC19P053334 |
| EGFR-AS1 | GC07M055179 |
| PTCH1 | GC09M095442 |
| EPCAM | GC02P047345 |
| MIR15A | GC13M050049 |
| TNF | GC06P076864 |
| POLE | GC12M132666 |
| VEGFA | GC06P043770 |
| MUTYH | GC01M045329 |
| ESR1 | GC06P151656 |
| CDK4 | GC12M057743 |
| VHL | GC03P012055 |
| ROS1 | GC06M117287 |
| MTOR | GC01M011106 |
| MIR34C | GC11P111675 |
| MIR210 | GC11M002651 |
| MIR98 | GC0XM053622 |
| IRF1 | GC05M132440 |
| NF1 | GC17P031094 |
| BAX | GC19P048954 |
| PTPRC | GC01P198607 |
| TSC2 | GC16P009895 |
| LRRC56 | GC11P000518 |
| MIR29A | GC07M130876 |
| PRKN | GC06M161348 |
| ERCC6 | GC10M049454 |
| FAS | GC10P090723 |
| MIR34B | GC11P111686 |
| MIR93 | GC07M101539 |
| FLCN | GC17M017206 |
| TNFRSF10B | GC08M023020 |
| SMARCA4 | GC19P010932 |
| MIR143 | GC05P149410 |
| RAD51D | GC17M035092 |
| EGF | GC04P109912 |
| MIR155 | GC21P025573 |
| RAD51C | GC17P058692 |
| RAD50 | GC05P132556 |
| MDM2 | GC12P068808 |
| MIR29C | GC01M207802 |
| CDKN1A | GC06P077011 |
| MIR18A | GC13P091536 |
| MIR19A | GC13P091532 |
| MIRLET7D | GC09P094178 |
| TSC1 | GC09M132891 |
| POLD1 | GC19P052405 |
| IL1B | GC02M112829 |
| MIRLET7C | GC21P016551 |
| MIR133B | GC06P052148 |
| MIR146A | GC05P160485 |
| MRE11 | GC11M095776 |
| SRC | GC20P037344 |
| SLC22A18 | GC11P002899 |
| MIRLET7G | GC03M052268 |
| MAPK1 | GC22M021759 |
| PTPN11 | GC12P112418 |
| FBXW7 | GC04M152321 |
| MIR137 | GC01M098046 |
| NKX2-1 | GC14M036516 |
| IFNG | GC12M068154 |
| MUC1 | GC01M155185 |
| PPP2R1B | GC11M111695 |
| MIR486-1 | GC08M041660 |
| AR | GC0XP067544 |
| IL10 | GC01M206767 |
| CXCL8 | GC04P073740 |
| EP300 | GC22P041091 |
| MIR183 | GC07M129818 |
| ING1 | GC13P110712 |
| CD274 | GC09P005450 |
| MIR499A | GC20P034990 |
| MIR107 | GC10M089600 |
| CASP3 | GC04M184627 |
| MIR429 | GC01P003360 |
| BAP1 | GC03M052401 |
| PPARG | GC03P012287 |
| MIRLET7B | GC22P046119 |
| MMP9 | GC20P046008 |
| NOTCH1 | GC09M137332 |
| AXIN2 | GC17M065528 |
| CXCR4 | GC02M136114 |
| JAK3 | GC19M017824 |
| ERCC2 | GC19M045349 |
| CYP2A6 | GC19M040843 |
| FANCC | GC09M095099 |
| TP73 | GC01P003652 |
| HIF1A | GC14P061695 |
| MIR185 | GC22P033275 |
| TGFBR1 | GC09P099104 |
| PIK3R1 | GC05P068215 |
| MIRLET7E | GC19P053332 |
| MMP2 | GC16P055390 |
| MMP1 | GC11M102810 |
| BCL10 | GC01M085265 |
| MIR373 | GC19P062508 |
| MIR203A | GC14P108823 |
| MIR31 | GC09M021705 |
| MIR130A | GC11P057641 |
| MIR372 | GC19P062507 |
| DDR2 | GC01P162631 |
| PTGS2 | GC01M186640 |
| CD44 | GC11P035139 |
| MIR30D | GC08M134804 |
| TP63 | GC03P189598 |
| RAD51 | GC15P040694 |
| FH | GC01M241499 |
| RAG1 | GC11P036534 |
| BLM | GC15P090717 |
| FGFR1 | GC08M038400 |
| MIR197 | GC01P109549 |
| CTLA4 | GC02P203867 |
| MEN1 | GC11M064803 |
| MIR30E | GC01P040754 |
| JAK2 | GC09P004985 |
| MIR127 | GC14P108752 |
| FHIT | GC03M059747 |
| DLEC1 | GC03P038038 |
| NFE2L2 | GC02M177227 |
| STAT1 | GC02M190908 |
| IGF2 | GC11M002733 |
| MIRLET7A3 | GC22P046112 |
| MAP3K8 | GC10P030489 |
| MIR128-2 | GC03P035750 |
| IL7R | GC05P035852 |
| KDR | GC04M055078 |
| MXRA5 | GC0XM003308 |
| GATA2 | GC03M128479 |
| ADA | GC20M044620 |
| MIR27A | GC19M014387 |
| TERC | GC03M169765 |
| SETD2 | GC03M047033 |
| AURKA | GC20M056370 |
| JUN | GC01M058780 |
| CTNNA1 | GC05P138613 |
| MSH3 | GC05P080654 |
| BIRC5 | GC17P078214 |
| IL2 | GC04M122451 |
| PRKD1 | GC14M029576 |
| HNF1B | GC17M037686 |
| MIR106B | GC07M101540 |
| ABCB1 | GC07M087504 |
| FGF2 | GC04P122826 |
| HLA-DRB1 | GC06M032578 |
| WWOX | GC16P078099 |
| MIR223 | GC0XP066018 |
| PDCD1 | GC02M241849 |
| PDGFRA | GC04P054229 |
| IGF1R | GC15P098648 |
| RAG2 | GC11M036575 |
| NFKBIA | GC14M035401 |
| XRCC3 | GC14M103697 |
| ITGB1 | GC10M032932 |
| MALAT1 | GC11P068989 |
| ERCC1 | GC19M062024 |
| BCL2L1 | GC20M031664 |
| MIR148A | GC07M025993 |
| CYP1A1 | GC15M074719 |
| SMARCB1 | GC22P023786 |
| MIR182 | GC07M129770 |
| CEACAM5 | GC19P051844 |
| GSTP1 | GC11P067583 |
| CD4 | GC12P006786 |
| XRCC2 | GC07M152644 |
| KLF6 | GC10M003779 |
| GSTM1 | GC01P109687 |
| IL4 | GC05P132673 |
| HGF | GC07M081699 |
| XRCC1 | GC19M043543 |
| RHOA | GC03M049359 |
| ABL1 | GC09P130713 |
| CDC73 | GC01P193121 |
| WT1 | GC11M032365 |
| SDHB | GC01M017842 |
| PCNA | GC20M005114 |
| SLMAP | GC03P057858 |
| NFKB1 | GC04P102501 |
| MIR204 | GC09M070809 |
| CAV1 | GC07P116524 |
| KRT19 | GC17M041523 |
| PGBD3 | GC10M049517 |
| ERBB3 | GC12P057065 |
| OGG1 | GC03P011996 |
| SOX9 | GC17P072121 |
| MGMT | GC10P129467 |
| RAC1 | GC07P006377 |
| CDK2 | GC12P055966 |
| ICAM1 | GC19P010310 |
| KITLG | GC12M088492 |
| MKI67 | GC10M128096 |
| RAF1 | GC03M012583 |
| CDKN2B | GC09M022002 |
| MIR16-1 | GC13M050048 |
| CCL2 | GC17P034255 |
| CARD11 | GC07M002906 |
| MITF | GC03P069788 |
| CXCL12 | GC10M044294 |
| PTK2 | GC08M140657 |
| SOX2 | GC03P181711 |
| XIAP | GC0XP123859 |
| CSF2 | GC05P132073 |
| HOXB13 | GC17M048725 |
| MIR214 | GC01M172234 |
| KRT7 | GC12P052232 |
| TLR2 | GC04P153684 |
| EZH2 | GC07M148807 |
| SPP1 | GC04P087975 |
| FOXP3 | GC0XM049250 |
| MAPK8 | GC10P048306 |
| PARP1 | GC01M226360 |
| BCL6 | GC03M187721 |
| IGF1 | GC12M102395 |
| NCAM1 | GC11P112961 |
| ENG | GC09M127815 |
| CSF3 | GC17P040015 |
| MCL1 | GC01M151420 |
| H19 | GC11M001995 |
| B2M | GC15P044711 |
| MIR451A | GC17M028861 |
| MLH3 | GC14M075013 |
| CDK1 | GC10P060772 |
| MIR100 | GC11M122152 |
| DCC | GC18P052340 |
| CASP9 | GC01M015491 |
| PDGFRB | GC05M150113 |
| MIR23B | GC09P095085 |
| SMAD3 | GC15P067063 |
| SUFU | GC10P102503 |
| IL2RA | GC10M006010 |
| MIR10B | GC02P176150 |
| FN1 | GC02M215360 |
| RARB | GC03P024689 |
| MIR106A | GC0XM134219 |
| TGFA | GC02M070447 |
| IDH1 | GC02M208236 |
| MIR96 | GC07M129774 |
| CREBBP | GC16M006452 |
| MIR150 | GC19M049500 |
| CDK6 | GC07M092604 |
| ING3 | GC07P120950 |
| MIR140 | GC16P069934 |
| ERBB4 | GC02M211375 |
| ENO2 | GC12P006913 |
| IL7 | GC08M078689 |
| KRT5 | GC12M052514 |
| VIM | GC10P017227 |
| GNAS | GC20P058839 |
| MAPK3 | GC16M035783 |
| VEGFC | GC04M176683 |
| FLT1 | GC13M028300 |
| TYMS | GC18P000657 |
| NPM1 | GC05P171387 |
| SFTPC | GC08P022156 |
| MMP14 | GC14P030861 |
| BUB1B | GC15P040161 |
| FLT3 | GC13M028003 |
| CFTR | GC07P117287 |
| MSR1 | GC08M016107 |
| RASSF1 | GC03M050329 |
| RUNX1 | GC21M034787 |
| CCNA2 | GC04M121816 |
| PLAU | GC10P073909 |
| TNFSF10 | GC03M172505 |
| PECAM1 | GC17M064319 |
| ABCG2 | GC04M088090 |
| POU5F1 | GC06M061255 |
| DNMT1 | GC19M010133 |
| CCR6 | GC06P167111 |
| HLA-B | GC06M061261 |
| ERCC4 | GC16P013920 |
| CCNB1 | GC05P069167 |
| SLC2A1 | GC01M042925 |
| PGR | GC11M101030 |
| XPA | GC09M097654 |
| ESR2 | GC14M064084 |
| MYCN | GC02P015949 |
| CD40 | GC20P046118 |
| FANCM | GC14P045135 |
| MAPK14 | GC06P077003 |
| MIR146B | GC10P102436 |
| EPHB2 | GC01P022710 |
| MIR195 | GC17M007018 |
| MIRLET7A1 | GC09P094175 |
| ZAP70 | GC02P097734 |
| IDH2 | GC15M090083 |
| PROM1 | GC04M015965 |
| TLR4 | GC09P117704 |
| LZTS1 | GC08M020246 |
| CYCS | GC07M025118 |
| YAP1 | GC11P102110 |
| KEAP1 | GC19M010486 |
| KRT18 | GC12P052948 |
| RARA | GC17P040309 |
| MIR25 | GC07M100093 |
| HOTAIR | GC12M053962 |
| FLT4 | GC05M180607 |
| MIR335 | GC07P130496 |
| CD34 | GC01M207880 |
| SDHD | GC11P112087 |
| HLA-A | GC06P076821 |
| POT1 | GC07M124822 |
| HFE | GC06P026087 |
| ARID1A | GC01P026693 |
| SMAD2 | GC18M047809 |
| CDC42 | GC01P022264 |
| SERPINA1 | GC14M094376 |
| VCAM1 | GC01P100719 |
| CCL5 | GC17M035871 |
| HAVCR2 | GC05M157063 |
| SP1 | GC12P053380 |
| HBB | GC11M006229 |
| KRT8 | GC12M052897 |
| CASP10 | GC02P201182 |
| PRKCD | GC03P053156 |
| ZEB1 | GC10P031318 |
| CDH2 | GC18M027950 |
| ABCC1 | GC16P015949 |
| PLK1 | GC16P024137 |
| NF2 | GC22P029603 |
| NTRK1 | GC01P156815 |
| CADM1 | GC11M115169 |
| RELA | GC11M065653 |
| MTHFR | GC01M011785 |
| DNMT3A | GC02M025228 |
| IL1RN | GC02P120921 |
| SYK | GC09P091849 |
| TOP2A | GC17M040388 |
| GREM1 | GC15P038163 |
| MIR199B | GC09M128244 |
| PDPN | GC01P013583 |
| MIR192 | GC11M064891 |
| NME1 | GC17P051161 |
| CHGA | GC14P092929 |
| EPHA2 | GC01M016124 |
| MIR193B | GC16P014307 |
| GLI1 | GC12P057460 |
| DNMT3B | GC20P032762 |
| IGFBP3 | GC07M045912 |
| MEG3 | GC14P109055 |
| HMOX1 | GC22P035380 |
| FOS | GC14P075278 |
| BMP2 | GC20P006767 |
| SYP | GC0XM049187 |
| MMP7 | GC11M102425 |
| TIMP1 | GC0XP047583 |
| NQO1 | GC16M069706 |
| PIK3CG | GC07P106865 |
| IL3 | GC05P132060 |
| ALB | GC04P073397 |
| TYMP | GC22M050525 |
| HLA-G | GC06P076812 |
| PDGFB | GC22M055561 |
| TWIST1 | GC07M019020 |
| CHEK1 | GC11P125625 |
| GJA1 | GC06P121436 |
| CD28 | GC02P203706 |
| THBS1 | GC15P039581 |
| TET2 | GC04P105145 |
| ATR | GC03M142449 |
| CREB1 | GC02P207529 |
| SHH | GC07M155799 |
| HSP90AA1 | GC14M102080 |
| ABRAXAS1 | GC04M083460 |
| U2AF1 | GC21M043092 |
| MUC4 | GC03M195746 |
| AKT2 | GC19M040230 |
| ANXA5 | GC04M121667 |
| ERCC5 | GC13P102845 |
| E2F1 | GC20M033675 |
| BUB1 | GC02M110637 |
| PRKCA | GC17P066302 |
| CRNN | GC01M152381 |
| MIR375 | GC02M219001 |
| MIR331 | GC12P095308 |
| INS | GC11M002159 |
| SFTPB | GC02M085657 |
| UCA1 | GC19P015828 |
| CDKN3 | GC14P054398 |
| TFRC | GC03M196027 |
| MAD1L1 | GC07M001815 |
| HNF1A | GC12P125499 |
| CEACAM6 | GC19P041750 |
| ETV6 | GC12P011649 |
| CD8A | GC02M086784 |
| PPM1D | GC17P060600 |
| PHB1 | GC17M049406 |
| HMGB1 | GC13M030456 |
| MPO | GC17M058269 |
| MIR483 | GC11M002736 |
| SMARCE1 | GC17M040624 |
| CTAG1B | GC0XM154617 |
| KRT20 | GC17M040875 |
| PAX5 | GC09M036828 |
| BIRC3 | GC11P102317 |
| PTHLH | GC12M027959 |
| RNASEL | GC01M182573 |
| PLAUR | GC19M043646 |
| MIR22 | GC17M001713 |
| WRAP53 | GC17P010614 |
| SFTPA1 | GC10P091431 |
| MIR122 | GC18P058451 |
| MIR144 | GC17M033777 |
| SOD2 | GC06M159669 |
| MIR193A | GC17P031559 |
| BSG | GC19P000571 |
| PMS1 | GC02P189784 |
| DLC1 | GC08M013083 |
| DSP | GC06P007541 |
| SNAI2 | GC08M048917 |
| KRT14 | GC17M041582 |
| IL17A | GC06P052186 |
| BMP6 | GC06P007726 |
| LGALS3 | GC14P055124 |
| FGFR4 | GC05P177086 |
| SNAI1 | GC20P049982 |
| TIMP2 | GC17M078852 |
| IL13 | GC05P132656 |
| HLA-DQB1 | GC06M061358 |
| MIR142 | GC17M058331 |
| CCN2 | GC06M131948 |
| MYB | GC06P135180 |
| CCR7 | GC17M041052 |
| HSPB1 | GC07P076302 |
| TOP1 | GC20P041028 |
| CA9 | GC09P035673 |
| MIR296 | GC20M058817 |
| XPC | GC03M019709 |
| CYP1B1 | GC02M038066 |
| MUC16 | GC19M008848 |
| MIF | GC22P023894 |
| SDHC | GC01P161314 |
| AREG | GC04P074445 |
| SMO | GC07P131429 |
| CD36 | GC07P080369 |
| RTEL1 | GC20P063658 |
| RIPK1 | GC06P003155 |
| MIR342 | GC14P100109 |
| RNF6 | GC13M026132 |
| CBL | GC11P119206 |
| LOX | GC05M122063 |
| CCND3 | GC06M041934 |
| FANCD2 | GC03P010026 |
| IFNA1 | GC09P021577 |
| CFLAR | GC02P201117 |
| PVT1 | GC08P127849 |
| ITGA3 | GC17P050055 |
| MAP3K1 | GC05P056815 |
| SMAD7 | GC18M048919 |
| THY1 | GC11M119417 |
| ZFHX3 | GC16M072782 |
| ERCC3 | GC02M127257 |
| EWSR1 | GC22P033676 |
| FOXN1 | GC17P028506 |
| RPS6KB1 | GC17P059893 |
| ACTB | GC07M005527 |
| LUCAT1 | GC05M091054 |
| BMI1 | GC10P022326 |
| CCAT1 | GC08M127207 |
| GRP | GC18P059220 |
| CRP | GC01M159725 |
| CDC25A | GC03M048173 |
| MME | GC03P155024 |
| MMP3 | GC11M102835 |
| MIR181A1 | GC01M198860 |
| GATA1 | GC0XP048786 |
| TYR | GC11P089177 |
| ELAC2 | GC17M012991 |
| CEACAM3 | GC19P041796 |
| SERPINA3 | GC14P094612 |
| IL1A | GC02M112773 |
| HDAC1 | GC01P032292 |
| NRG1 | GC08P031639 |
| TIMP3 | GC22P033834 |
| LEP | GC07P128241 |
| RASA1 | GC05P087267 |
| MIR206 | GC06P052144 |
| NOS2 | GC17M027756 |
| BCL2L11 | GC02P111119 |
| PALLD | GC04P168497 |
| ECT2 | GC03P172750 |
| GPC3 | GC0XM133535 |
| CCNE1 | GC19P029811 |
| NOS3 | GC07P150990 |
| TGFB2 | GC01P218345 |
| MIR10A | GC17M048579 |
| CD40LG | GC0XP136649 |
| MCM4 | GC08P047965 |
| NEAT1 | GC11P068984 |
| DPYD | GC01M097015 |
| PDCD4 | GC10P110871 |
| HMMR | GC05P163480 |
| FANCE | GC06P076991 |
| PTK2B | GC08P027311 |
| CCAT2 | GC08P127400 |
| MIR26A1 | GC03P037969 |
| RUNX3 | GC01M024899 |
| MIRLET7I | GC12P062606 |
| MIR199A1 | GC19M010817 |
| TNFRSF1A | GC12M006328 |
| CTTN | GC11P070398 |
| SERPINE1 | GC07P101127 |
| SKP2 | GC05P036151 |
| CDKN2B-AS1 | GC09P021994 |
| HSPA5 | GC09M125234 |
| LMNA | GC01P156082 |
| MT-CYB | GCMTP014749 |
| DAPK1 | GC09P087497 |
| MIR128-1 | GC02P135665 |
| FGF7 | GC15P049423 |
| IKBKB | GC08P042271 |
| EDN1 | GC06P012256 |
| CTNND1 | GC11P058081 |
| PLA2G2A | GC01M019975 |
| TUG1 | GC22P030969 |
| MIR196A2 | GC12P054900 |
| HDAC9 | GC07P018086 |
| RRAS2 | GC11M014299 |
| MUC5B | GC11P001583 |
| CALR | GC19P012938 |
| RAD54L | GC01P046313 |
| CDC25C | GC05M138285 |
| NRP1 | GC10M033177 |
| ITGB3 | GC17P051053 |
| MIR15B | GC03P160404 |
| SIRT1 | GC10P067884 |
| FOXM1 | GC12M002857 |
| POSTN | GC13M037562 |
| DHFR | GC05M080626 |
| PIK3CD | GC01P009629 |
| EIF4E | GC04M098879 |
| ITGAV | GC02P186589 |
| CLDN7 | GC17M007259 |
| BCL11B | GC14M099169 |
| MIR424 | GC0XM134685 |
| CSF1 | GC01P109911 |
| IFI27 | GC14P094104 |
| MIR23A | GC19M014388 |
| GAS5 | GC01M173947 |
| NTHL1 | GC16M006280 |
| IRS1 | GC02M226731 |
| KLRK1 | GC12M019788 |
| VDR | GC12M047841 |
| BMP4 | GC14M053949 |
| SOS1 | GC02M039016 |
| BAK1 | GC06M033572 |
| BAD | GC11M084618 |
| KDM4C | GC09P006720 |
| GATA3 | GC10P008045 |
| CCND2 | GC12P018505 |
| MAX | GC14M065073 |
| TNFRSF10A | GC08M023190 |
| HSPA4 | GC05P133051 |
| AXL | GC19P041219 |
| FOXO1 | GC13M040555 |
| CTSB | GC08M011842 |
| RSPO1 | GC01M037612 |
| NSD1 | GC05P177134 |
| PTPRJ | GC11P048002 |
| DKC1 | GC0XP154762 |
| IL15 | GC04P141636 |
| MMP13 | GC11M102942 |
| VEGFD | GC0XM015345 |
| ACTA2 | GC10M088935 |
| MIR191 | GC03M050871 |
| MUC5AC | GC11P001581 |
| CD24 | GC06M106969 |
| STK4 | GC20P044966 |
| ETS1 | GC11M128458 |
| FSCN1 | GC07P005592 |
| TRB | GC07P147873 |
| IGF2R | GC06P159969 |
| KLF4 | GC09M107484 |
| FBN1 | GC15M048408 |
| EGR1 | GC05P138465 |
| NFKB2 | GC10P102394 |
| LHCGR | GC02M048686 |
| CIB1 | GC15M090229 |
| PPP2R1A | GC19P061809 |
| APEX1 | GC14P020455 |
| EZR | GC06M158765 |
| FOXO3 | GC06P108559 |
| FGF10 | GC05M044340 |
| JUP | GC17M041754 |
| MIR30A | GC06M071403 |
| CSF1R | GC05M150053 |
| SPARC | GC05M151661 |
| GSK3B | GC03M119821 |
| EPO | GC07P100720 |
| ITGA6 | GC02P172427 |
| H2AC18 | GC01M151368 |
| GJB2 | GC13M020187 |
| ELANE | GC19P002165 |
| LIG4 | GC13M108207 |
| PRF1 | GC10M070597 |
| ICOSLG | GC21M044222 |
| MVP | GC16P039370 |
| ITK | GC05P157158 |
| MSMB | GC10M046033 |
| ITGA5 | GC12M054793 |
| GSTT1 | GC22Mi00270 |
| MT-CO1 | GCMTP005906 |
| ICOS | GC02P203937 |
| CYP2E1 | GC10P133520 |
| MIR181A2 | GC09P124692 |
| PXN | GC12M120210 |
| GDNF | GC05M037812 |
| NAT2 | GC08P018391 |
| MCC | GC05M113022 |
| LCK | GC01P032251 |
| STAT5B | GC17M042199 |
| PCAT1 | GC08P126553 |
| LAMC2 | GC01P183186 |
| SHC1 | GC01M154962 |
| ALDH1A1 | GC09M072900 |
| COL1A1 | GC17M050183 |
| MIR224 | GC0XM151958 |
| CD79A | GC19P041877 |
| HMGA2 | GC12P065824 |
| XRCC5 | GC02P216107 |
| OPCML | GC11M132405 |
| ANXA1 | GC09P073151 |
| SOD1 | GC21P031659 |
| RECQL | GC12M021468 |
| ABCC2 | GC10P099782 |
| MYD88 | GC03P038139 |
| CDKN2C | GC01P050960 |
| MXI1 | GC10P110208 |
| MIR16-2 | GC03P160413 |
| IL6R | GC01P154405 |
| TNFRSF1B | GC01P012167 |
| YWHAE | GC17M002492 |
| SERPINB5 | GC18P063476 |
| IL4R | GC16P027659 |
| CASC2 | GC10P118046 |
| DROSHA | GC05M031401 |
| ANGPT2 | GC08M006499 |
| PKM | GC15M072199 |
| S100A4 | GC01M153543 |
| TRIP13 | GC05P000892 |
| TLR9 | GC03M052222 |
| ZEB2 | GC02M144384 |
| KMT2D | GC12M049018 |
| ACE | GC17P063477 |
| CYP19A1 | GC15M051208 |
| GZMB | GC14M024630 |
| PARN | GC16M014435 |
| NQO2 | GC06P003166 |
| EPHB4 | GC07M101609 |
| IDO1 | GC08P039891 |
| SFTPD | GC10M079937 |
| BECN1 | GC17M042810 |
| CLU | GC08M027596 |
| GAPDH | GC12P018545 |
| AXIN1 | GC16M000287 |
| PAX8 | GC02M113215 |
| INSR | GC19M007112 |
| KLK3 | GC19P050854 |
| ALCAM | GC03P105366 |
| CYP2D6 | GC22M042126 |
| CCR5 | GC03P046383 |
| MIR9-1 | GC01M156420 |
| MSLN | GC16P009836 |
| KCNN4 | GC19M061975 |
| COPA | GC01M160288 |
| YY1 | GC14P100238 |
| SERPINB3 | GC18M063655 |
| RECQL4 | GC08M145260 |
| SST | GC03M187668 |
| IL5 | GC05M132541 |
| MC1R | GC16P089912 |
| PDGFRL | GC08P017576 |
| SFTPA2 | GC10M081337 |
| WRN | GC08P031033 |
| CXCR2 | GC02P218125 |
| TRA | GC14P021621 |
| TXN | GC09M110243 |
| CCNH | GC05M087311 |
| POLK | GC05P075511 |
| NTRK3 | GC15M087859 |
| PRKCI | GC03P170222 |
| FLNA | GC0XM154348 |
| DDB2 | GC11P047237 |
| HERC2 | GC15M028111 |
| LGALS1 | GC22P037675 |
| CYP3A4 | GC07M099759 |
| TNFSF11 | GC13P042562 |
| HLA-DPB1 | GC06P076901 |
| CD247 | GC01M167399 |
| CCL3 | GC17M036088 |
| XRCC6 | GC22P041622 |
| SRD5A2 | GC02M031522 |
| SOCS3 | GC17M078356 |
| FGF1 | GC05M142555 |
| ENO1 | GC01M008861 |
| IL18 | GC11M112143 |
| CTSD | GC11M001752 |
| TYK2 | GC19M010350 |
| NANOG | GC12P007787 |
| PTCH2 | GC01M044819 |
| AFP | GC04P073431 |
| BIRC2 | GC11P102347 |
| LOC110806263 | GC05P001294 |
| SELE | GC01M169722 |
| CASP7 | GC10P113679 |
| IFNA2 | GC09M021384 |
| DMD | GC0XM031097 |
| EDNRA | GC04P147480 |
| NR3C1 | GC05M143277 |
| DOCK8 | GC09P000214 |
| AURKB | GC17M010127 |
| MIR215 | GC01M220117 |
| CD82 | GC11P044586 |
| MIR196A1 | GC17M048632 |
| CDH13 | GC16P082626 |
| RUNX2 | GC06P077087 |
| CEACAM1 | GC19M042507 |
| GSN | GC09P121201 |
| TINF2 | GC14M024258 |
| APOE | GC19P051999 |
| CDX2 | GC13M027962 |
| MIR139 | GC11M072615 |
| JAG1 | GC20M010637 |
| ILK | GC11P006604 |
| PKHD1 | GC06M061651 |
| TCF7L2 | GC10P112950 |
| TEK | GC09P027109 |
| CXCR3 | GC0XM071615 |
| ANPEP | GC15M089784 |
| ITGAM | GC16P039480 |
| ITGB4 | GC17P075721 |
| ASXL1 | GC20P032585 |
| KCNQ1OT1 | GC11M002796 |
| AHR | GC07P016916 |
| STMN1 | GC01M025884 |
| MALT1 | GC18P058671 |
| EPAS1 | GC02P046293 |
| HLA-DQA1 | GC06P076895 |
| THPO | GC03M184371 |
| CHUK | GC10M100188 |
| VWF | GC12M005917 |
| CEBPA | GC19M033299 |
| XPO1 | GC02M061445 |
| ASCL1 | GC12P102957 |
| ANGPT1 | GC08M107246 |
| CD9 | GC12P018518 |
| DPP4 | GC02M161992 |
| SCGB1A1 | GC11P062405 |
| FANCA | GC16M089801 |
| ANXA2 | GC15M060347 |
| ALOX5 | GC10P045374 |
| RECK | GC09P036036 |
| TAP1 | GC06M061365 |
| CASC9 | GC08M075132 |
| ITGB2 | GC21M044885 |
| IL2RB | GC22M037125 |
| DMBT1 | GC10P122560 |
| HLA-C | GC06M061260 |
| ELN | GC07P074027 |
| SF3B1 | GC02M197401 |
| MAP2K4 | GC17P012020 |
| FASN | GC17M082078 |
| YBX1 | GC01P042682 |
| DDR1 | GC06P076844 |
| MIR149 | GC02P240456 |
| RPL5 | GC01P092832 |
| SQSTM1 | GC05P179806 |
| MIR9-3 | GC15P089368 |
| FANCG | GC09M035073 |
| BRD4 | GC19M015236 |
| BDNF | GC11M027654 |
| MIR30B | GC08M134800 |
| TFAP2A | GC06M010393 |
| CD80 | GC03M119524 |
| ODC1 | GC02M010432 |
| STAT6 | GC12M057095 |
| FCGR2A | GC01P161505 |
| CHRNA5 | GC15P078565 |
| MIR99A | GC21P016539 |
| PTGER4 | GC05P040679 |
| PSCA | GC08P142670 |
| PTPN12 | GC07P077537 |
| ALDOA | GC16P030064 |
| ID1 | GC20P031605 |
| STAT5A | GC17P042287 |
| AGER | GC06M032180 |
| STIM1 | GC11P003855 |
| TTN | GC02M178525 |
| TFE3 | GC0XM049028 |
| FADD | GC11P070203 |
| TGFB3 | GC14M075958 |
| KMT2A | GC11P118436 |
| GFAP | GC17M044905 |
| GATA6 | GC18P022169 |
| HSPA8 | GC11M123057 |
| WNT5A | GC03M055465 |
| CD3D | GC11M118338 |
| HNF4A | GC20P044355 |
| EPHX1 | GC01P225810 |
| CR2 | GC01P207454 |
| CHRNA3 | GC15M079623 |
| FOXE1 | GC09P097853 |
| NGF | GC01M115285 |
| TKT | GC03M053224 |
| GDF15 | GC19P050952 |
| CXADR | GC21P017513 |
| MAGEA3 | GC0XP152698 |
| RACK1 | GC05M181618 |
| THBD | GC20M023026 |
| NPC1 | GC18M023506 |
| JAK1 | GC01M064833 |
| TUBB | GC06P076841 |
| SOCS1 | GC16M011783 |
| EPOR | GC19M011377 |
| CALCA | GC11M014945 |
| XIST | GC0XM073820 |
| NOTCH3 | GC19M015159 |
| CDC6 | GC17P040287 |
| MDM4 | GC01P204516 |
| MT-CO2 | GCMTP007587 |
| DES | GC02P219418 |
| CEACAM7 | GC19M041673 |
| TG | GC08P132866 |
| GATA4 | GC08P011676 |
| SPRY4-IT1 | GC05M142318 |
| ROCK1 | GC18M020946 |
| MIR324 | GC17M007223 |
| CAT | GC11P034460 |
| HABP2 | GC10P113550 |
| PDCD1LG2 | GC09P005510 |
| PRKDC | GC08M047773 |
| TLR3 | GC04P186059 |
| FOXP1 | GC03M070926 |
| PDGFA | GC07M000497 |
| GAST | GC17P041712 |
| ACTC1 | GC15M034790 |
| HBEGF | GC05M140332 |
| CDKN1C | GC11M002905 |
| MAGEA4 | GC0XP151912 |
| POLR1C | GC06P077073 |
| SOX4 | GC06P021593 |
| STN1 | GC10M103917 |
| ADRB2 | GC05P148825 |
| TJP1 | GC15M029699 |
| SLC4A1 | GC17M044786 |
| LEPR | GC01P065421 |
| ERG | GC21M038367 |
| PTPN22 | GC01M113813 |
| SMPD1 | GC11P006390 |
| FCGR3A | GC01M161541 |
| CDH5 | GC16P066366 |
| CP | GC03M149162 |
| COL7A1 | GC03M048564 |
| CXCL10 | GC04M076021 |
| DUSP1 | GC05M172768 |
| GRB2 | GC17M075318 |
| NOTCH2 | GC01M119911 |
| TMC8 | GC17P078130 |
| HOTTIP | GC07P027198 |
| CDH3 | GC16P068637 |
| TCF3 | GC19M001609 |
| APAF1 | GC12P098645 |
| ELAVL1 | GC19M007958 |
| MST1R | GC03M050930 |
| MS4A1 | GC11P060503 |
| AKT3 | GC01M243488 |
| P2RX7 | GC12P125508 |
| MAP2K2 | GC19M004090 |
| F2 | GC11P046720 |
| ITGA4 | GC02P181456 |
| RRM1 | GC11P004115 |
| LEF1 | GC04M108047 |
| MYCL | GC01M039895 |
| PLCG1 | GC20P041136 |
| NES | GC01M156668 |
| ARAF | GC0XP047562 |
| RARS1 | GC05P168487 |
| NDUFA13 | GC19P019515 |
| ROBO1 | GC03M078597 |
| ADAR | GC01M154582 |
| SPRY4 | GC05M142310 |
| MYO1B | GC02P191246 |
| GSTM3 | GC01M109733 |
| NUTM1 | GC15P034343 |
| SART1 | GC11P069032 |
| COMT | GC22P019941 |
| MT-ND1 | GCMTP003309 |
| NAPSA | GC19M062271 |
| PRKAA1 | GC05M040759 |
| SDC1 | GC02M020200 |
| DKK1 | GC10P052314 |
| CCN1 | GC01P085581 |
| CD81 | GC11P002505 |
| PGF | GC14M074941 |
| MIR24-2 | GC19M014386 |
| RHOB | GC02P020447 |
| F3 | GC01M094570 |
| CCKBR | GC11P006259 |
| RPSA | GC03P039406 |
| NTRK2 | GC09P084668 |
| ADH1C | GC04M099336 |
| SLC19A1 | GC21M045493 |
| MTDH | GC08P097643 |
| MIR320A | GC08M022262 |
| WEE1 | GC11P009573 |
| GNAQ | GC09M077716 |
| HDAC2 | GC06M113933 |
| CCL11 | GC17P034285 |
| GLI2 | GC02P120735 |
| DDX3X | GC0XP041333 |
| ALDH2 | GC12P111766 |
| LYN | GC08P055879 |
| MAGEA1 | GC0XP153179 |
| HPC3 | GC20U900275 |
| CYTOR | GC02P087941 |
| SNHG1 | GC11M084565 |
| SREBF1 | GC17M017810 |
| TNC | GC09M115019 |
| ZMYND10 | GC03M050965 |
| PCAP | GC01U990208 |
| HOPX | GC04M056647 |
| CALB2 | GC16P071358 |
| FYN | GC06M111660 |
| ADAM12 | GC10M126012 |
| S100B | GC21M050156 |
| MIR32 | GC09M109046 |
| MYO18B | GC22P025742 |
| HPC4 | GC07U901751 |
| IRF4 | GC06P000391 |
| LIF | GC22M030240 |
| RNF139 | GC08P124474 |
| HPGD | GC04M174490 |
| MIR29B1 | GC07M130877 |
| DIABLO | GC12M122208 |
| POLH | GC06P043576 |
| IGFBP2 | GC02P216632 |
| TCF4 | GC18M055222 |
| TRIM28 | GC19P058544 |
| HPC14 | GC11U901301 |
| PTPN13 | GC04P086594 |
| CD86 | GC03P122055 |
| MMP10 | GC11M102770 |
| UCHL1 | GC04P041256 |
| ZBTB16 | GC11P114059 |
| MIR449A | GC05M055171 |
| CD22 | GC19P035319 |
| HPC6 | GC22U900503 |
| HPC10 | GC08U901117 |
| MIR502 | GC0XP050014 |
| MMP12 | GC11M102862 |
| HPC5 | GC03U900622 |
| RABL3 | GC03M120686 |
| CASP1 | GC11M105025 |
| FAM13A | GC04M088725 |
| ATRX | GC0XM077504 |
| EPHA3 | GC03P089077 |
| CDK5 | GC07M151053 |
| NDRG1 | GC08M133237 |
| HPC7 | GC15U901009 |
| ITGAL | GC16P030472 |
| TLR5 | GC01M223643 |
| RPS19 | GC19P051849 |
| BAG1 | GC09M033245 |
| MIR661 | GC08M143945 |
| NUMA1 | GC11M072002 |
| KLRD1 | GC12P010226 |
| UGT1A1 | GC02P233760 |
| SELP | GC01M169558 |
| EML4 | GC02P042169 |
| KLF5 | GC13P073054 |
| BIRC7 | GC20P063235 |
| PML | GC15P073994 |
| HPC15 | GC19U901018 |
| IL12B | GC05M159314 |
| CXCR5 | GC11P118912 |
| HPSE | GC04M083292 |
| IKBKG | GC0XP154541 |
| IL11 | GC19M055364 |
| CCR3 | GC03P046276 |
| WNT4 | GC01M022190 |
| PAX6 | GC11M031784 |
| DIRC3 | GC02M217284 |
| NCOA3 | GC20P047501 |
| MSN | GC0XP065588 |
| RPL11 | GC01P023691 |
| SELL | GC01M169690 |
| RTEL1-TNFRSF6B | GC20P063657 |
| DLK1 | GC14P108874 |
| MIR532 | GC0XP050366 |
| CD55 | GC01P207321 |
| MIR181C | GC19P014002 |
| FLI1 | GC11P128686 |
| BMPR2 | GC02P202376 |
| MYLK | GC03M123610 |
| RPS27 | GC01P153991 |
| TNFRSF8 | GC01P012063 |
| CDC20 | GC01P043358 |
| PRTN3 | GC19P000840 |
| HSF1 | GC08P144291 |
| S100A8 | GC01M153391 |
| BCAR1 | GC16M075228 |
| RPS20 | GC08M056067 |
| GBA | GC01M155234 |
| LINC-ROR | GC18M057054 |
| CD3G | GC11P118344 |
| BCAR4 | GC16M011822 |
| CEBPB | GC20P050190 |
| RELB | GC19P052007 |
| ABCC3 | GC17P050634 |
| KRT13 | GC17M041500 |
| L1CAM | GC0XM153864 |
| DCK | GC04P070992 |
| TPX2 | GC20P031739 |
| CTSL | GC09P087725 |
| CXCR1 | GC02M218162 |
| CXCL1 | GC04P073869 |
| GRPR | GC0XP016141 |
| SH2B3 | GC12P111405 |
| NCOR1 | GC17M016029 |
| FARSB | GC02M222570 |
| SOX2-OT | GC03P180989 |
| CYP17A1 | GC10M102830 |
| NGFR | GC17P049495 |
| RMRP | GC09M035655 |
| ADIPOQ | GC03P186842 |
| CIITA | GC16P010880 |
| FGF4 | GC11M084877 |
| PRDX1 | GC01M045511 |
| SSTR2 | GC17P073165 |
| MTA1 | GC14P105419 |
| VTN | GC17M033771 |
| LTA | GC06P076862 |
| NOD2 | GC16P050693 |
| IFNB1 | GC09M021077 |
| KRT10 | GC17M040818 |
| BCOR | GC0XM040049 |
| NLRP1 | GC17M005499 |
| GLI3 | GC07M041960 |
| PAK1 | GC11M085111 |
| EIF2AK2 | GC02M037099 |
| SLC29A1 | GC06P044219 |
| CYP1A2 | GC15P074748 |
| AFAP1-AS1 | GC04P007756 |
| DCLRE1C | GC10M014897 |
| FUS | GC16P031180 |
| PRL | GC06M022287 |
| POMC | GC02M025160 |
| COL18A1 | GC21P045405 |
| NUP214 | GC09P131125 |
| FANCF | GC11M022600 |
| MYH9 | GC22M036281 |
| TRIM24 | GC07P138460 |
| HK2 | GC02P074833 |
| REST | GC04P056907 |
| CD2 | GC01P116754 |
| EIF4EBP1 | GC08P038359 |
| CASP2 | GC07P147632 |
| FGF8 | GC10M101770 |
| NAMPT | GC07M106248 |
| GPER1 | GC07P001885 |
| TFF1 | GC21M042362 |
| BMP7 | GC20M057168 |
| IL24 | GC01P206897 |
| FOXA1 | GC14M037589 |
| CD3E | GC11P118304 |
| S100A2 | GC01M153561 |
| CDK12 | GC17P039461 |
| PRKACA | GC19M014403 |
| TCIM | GC08P040153 |
| CSF3R | GC01M036466 |
| MCM2 | GC03P127598 |
| RHOH | GC04P040192 |
| CCNA1 | GC13P036431 |
| NLRP3 | GC01P247415 |
| FOSL1 | GC11M084714 |
| RIOX2 | GC03M097942 |
| IL1R1 | GC02P102136 |
| S100A9 | GC01P153357 |
| G6PD | GC0XM154531 |
| LDHA | GC11P018394 |
| XBP1 | GC22M028794 |
| WAS | GC0XP048676 |
| HSD17B1 | GC17P050735 |
| LCAL1 | GC06M079274 |
| PICALM | GC11M085957 |
| MTUS1 | GC08M017643 |
| GALC | GC14M087837 |
| MCAM | GC11M119308 |
| GRN | GC17P044345 |
| MIR138-1 | GC03P044115 |
| LAT | GC16P039282 |
| KIR3DL1 | GC19P062551 |
| ADH1B | GC04M099304 |
| F2R | GC05P076716 |
| WNT1 | GC12P049230 |
| BGLAP | GC01P156242 |
| ALPP | GC02P232378 |
| TGIF1 | GC18P003411 |
| TNFAIP3 | GC06P137866 |
| PLG | GC06P160702 |
| FANCL | GC02M058127 |
| CD5 | GC11P061114 |
| WIF1 | GC12M065050 |
| SFN | GC01P027280 |
| IL6ST | GC05M055935 |
| TRPV4 | GC12M109783 |
| CARMIL2 | GC16P067644 |
| PRKCE | GC02P045651 |
| SRSF2 | GC17M076734 |
| HSPG2 | GC01M021822 |
| MIR125B1 | GC11M122100 |
| SFRP1 | GC08M041262 |
| MIR338 | GC17M081126 |
| SPINK1 | GC05M147825 |
| BCL11A | GC02M060451 |
| CD63 | GC12M055725 |
| CCL4 | GC17P036103 |
| DRAIC | GC15P092253 |
| EXO1 | GC01P241847 |
| CTAG2 | GC0XM154651 |
| NECTIN4 | GC01M161071 |
| IL9 | GC05M135891 |
| WNT3 | GC17M046762 |
| NR1H2 | GC19P050329 |
| RCVRN | GC17M009896 |
| ETV4 | GC17M043527 |
| RRM2 | GC02P010123 |
| DANCR | GC04P052712 |
| NEDD9 | GC06M011183 |
| LYVE1 | GC11M010999 |
| SASH1 | GC06P148193 |
| MMP11 | GC22P023768 |
| CHI3L1 | GC01M203148 |
| LBR | GC01M225401 |
| MIR196B | GC07M027488 |
| LCP1 | GC13M046132 |
| KLRC1 | GC12M019793 |
| TNFRSF11A | GC18P062325 |
| ITGA2 | GC05P052989 |
| PTGS1 | GC09P122370 |
| PEBP1 | GC12P118135 |
| CASR | GC03P122183 |
| APP | GC21M025880 |
| TCF7 | GC05P134114 |
| TMC6 | GC17M078110 |
| MAPK10 | GC04M085990 |
| GNA11 | GC19P003094 |
| TNFRSF10D | GC08M023135 |
| ACVRL1 | GC12P051906 |
| ADAM17 | GC02M009488 |
| SNHG16 | GC17P076982 |
| NT5E | GC06P085449 |
| CD46 | GC01P207752 |
| HSPD1 | GC02M197486 |
| PTTG1 | GC05P160422 |
| TGM2 | GC20M038127 |
| MCM3 | GC06M052264 |
| PIK3CB | GC03M138652 |
| EPB41L3 | GC18M005382 |
| CDC27 | GC17M047117 |
| MIR181B1 | GC01M198858 |
| RBM6 | GC03P049940 |
| PTPN1 | GC20P050510 |
| TMPRSS2 | GC21M041464 |
| CASC8 | GC08M131040 |
| IRS2 | GC13M109752 |
| TCL1A | GC14M099754 |
| CCR4 | GC03P032951 |
| CCNG1 | GC05P163438 |
| IL21 | GC04M122612 |
| NSMCE3 | GC15M029269 |
| GADD45A | GC01P067685 |
| LRBA | GC04M150264 |
| BCYRN1 | GC02P047331 |
| MIR101-1 | GC01M065058 |
| MIAT | GC22P026646 |
| MAPK9 | GC05M180246 |
| HOXA11-AS | GC07P027184 |
| CD68 | GC17P007579 |
| RHBDF2 | GC17M076470 |
| BPIFA1 | GC20P033235 |
| CT83 | GC0XM116461 |
| FLNB | GC03P058008 |
| DDX5 | GC17M064498 |
| CLDN4 | GC07P073799 |
| RAD51B | GC14P067819 |
| SPHK1 | GC17P076376 |
| PIM1 | GC06P077019 |
| HDAC4 | GC02M239048 |
| MIR328 | GC16M067203 |
| MIR186 | GC01M071067 |
| MYOD1 | GC11P017741 |
| MTAP | GC09P021792 |
| EDNRB | GC13M077895 |
| TFEB | GC06M061505 |
| H2AX | GC11M119155 |
| TF | GC03P133805 |
| MIR124-1 | GC08M009903 |
| PNP | GC14P020468 |
| IL12RB1 | GC19M018058 |
| LAMA3 | GC18P023689 |
| LAMB3 | GC01M209614 |
| EPHA5 | GC04M065319 |
| CD244 | GC01M160830 |
| AIFM1 | GC0XM130129 |
| EPHA7 | GC06M093240 |
| E2F3 | GC06P020402 |
| FOXJ1 | GC17M076136 |
| ITGAE | GC17M004008 |
| GNRH1 | GC08M025419 |
| CD226 | GC18M069831 |
| MIR24-1 | GC09P095086 |
| RPS24 | GC10P078033 |
| PPARA | GC22P046150 |
| CYP2C19 | GC10P094762 |
| CYLD | GC16P050742 |
| FERMT1 | GC20M006074 |
| SLPI | GC20M045252 |
| GCLC | GC06M053497 |
| SLX4 | GC16M006446 |
| TLR7 | GC0XP012867 |
| CCR1 | GC03M046218 |
| CTSK | GC01M151456 |
| PARD3 | GC10M034110 |
| IVL | GC01P152881 |
| MIR27B | GC09P095097 |
| COL17A1 | GC10M104031 |
| FOXF1 | GC16P086510 |
| IGFBP5 | GC02M216672 |
| BTNL2 | GC06M032393 |
| DDIT3 | GC12M057516 |
| SEMA3B | GC03P050267 |
| TK1 | GC17M078175 |
| KRT17 | GC17M041619 |
| MIR130B | GC22P034073 |
| HNRNPA2B1 | GC07M026174 |
| PDPK1 | GC16P002537 |
| TDGF1 | GC03P046681 |
| PLEC | GC08M144096 |
| STAT4 | GC02M191029 |
| EREG | GC04P074366 |
| CCL20 | GC02P227835 |
| NCOR2 | GC12M124324 |
| LCN2 | GC09P128149 |
| CDCP1 | GC03M045082 |
| TERF1 | GC08P073003 |
| EBAG9 | GC08P109536 |
| TFPI2 | GC07M093885 |
| SLC11A1 | GC02P218382 |
| VCP | GC09M035056 |
| GPX1 | GC03M050895 |
| NET1 | GC10P005444 |
| CD276 | GC15P073683 |
| SCUBE3 | GC06P076986 |
| HNF1A-AS1 | GC12M122037 |
| CLTC | GC17P059619 |
| ACTN4 | GC19P038647 |
| CLDN1 | GC03M190305 |
| WNT7B | GC22M045920 |
| ETV1 | GC07M013891 |
| PPARGC1A | GC04M023755 |
| USP8 | GC15P050424 |
| AMER1 | GC0XM064185 |
| GPRC5A | GC12P018806 |
| GHR | GC05P042429 |
| CLEC7A | GC12M019786 |
| KNSTRN | GC15P040382 |
| FENDRR | GC16M086511 |
| PKD1 | GC16M006281 |
| CDC5L | GC06P044387 |
| MIR132 | GC17M002049 |
| CUL3 | GC02M224470 |
| PCAT6 | GC01P202810 |
| MICA | GC06P031399 |
| ESM1 | GC05M054977 |
| TIMELESS | GC12M056416 |
| CDCA7L | GC07M021900 |
| RPS10 | GC06M061429 |
| PRKCB | GC16P024142 |
| CD38 | GC04P017266 |
| ENPP2 | GC08M119556 |
| ALOX12 | GC17P006995 |
| PAWR | GC12M079574 |
| BID | GC22M017734 |
| MIR330 | GC19M062039 |
| ZFAS1 | GC20P049276 |
| PTPN3 | GC09M109375 |
| MAK | GC06M010762 |
| PSMB8 | GC06M032840 |
| PRKCZ | GC01P002050 |
| LINC00511 | GC17M072323 |
| SOX10 | GC22M056089 |
| CSK | GC15P074782 |
| FCGR2B | GC01P161663 |
| SEZ6L2 | GC16M035761 |
| MLANA | GC09P006341 |
| SERPINB4 | GC18M063637 |
| PDX1 | GC13P027921 |
| NEU1 | GC06M031857 |
| FOXC1 | GC06P001610 |
| RRM2B | GC08M102204 |
| MBL2 | GC10M052760 |
| PTPRT | GC20M042072 |
| SIRT3 | GC11M000215 |
| CUL1 | GC07P148697 |
| PRKCQ | GC10M006393 |
| PRDM14 | GC08M070051 |
| MIR17HG | GC13P091347 |
| BTC | GC04M074744 |
| CD59 | GC11M033704 |
| TINCR | GC19M005558 |
| TTK | GC06P080003 |
| SEMA3F | GC03P050269 |
| CD47 | GC03M108043 |
| TLX3 | GC05P171309 |
| TNFRSF11B | GC08M118923 |
| RPL15 | GC03P023916 |
| PIN1 | GC19P009835 |
| HDAC6 | GC0XP048801 |
| MIR497 | GC17M007022 |
| HP | GC16P072089 |
| DDX41 | GC05M177511 |
| SIX1 | GC14M060643 |
| DNAH8 | GC06P077030 |
| DTNBP1 | GC06M015523 |
| CD14 | GC05M140631 |
| SAT1 | GC0XP023784 |
| DYNC2H1 | GC11P103109 |
| TUSC2 | GC03M050960 |
| LOXL2 | GC08M023296 |
| MIR590 | GC07P074191 |
| BRDT | GC01P091949 |
| HLA-DPA1 | GC06M033064 |
| IREB2 | GC15P078437 |
| FOLH1 | GC11M084355 |
| AMACR | GC05M033986 |
| TLE1 | GC09M081583 |
| LPP | GC03P188153 |
| FANCI | GC15P089243 |
| SLC9A9 | GC03M143265 |
| PLK2 | GC05M058453 |
| F2RL1 | GC05P076818 |
| TTR | GC18P031557 |
| PPP2R2A | GC08P026292 |
| TRAF4 | GC17P049706 |
| TSG101 | GC11M018468 |
| MIR423 | GC17P030117 |
| KRT1 | GC12M052674 |
| MIR521-1 | GC19P053748 |
| ELAVL4 | GC01P050025 |
| CR1 | GC01P207496 |
| CCL17 | GC16P057411 |
| MED12 | GC0XP071118 |
| CD151 | GC11P001565 |
| FZR1 | GC19P003506 |
| NFATC1 | GC18P079395 |
| HMGA1 | GC06P076971 |
| RBM5 | GC03P050258 |
| RHOC | GC01M112701 |
| NKX2-5 | GC05M173232 |
| BCAM | GC19P051996 |
| RXRA | GC09P134317 |
| LIMD1 | GC03P045555 |
| MTHFD1 | GC14P064388 |
| MVK | GC12P109573 |
| NFATC2 | GC20M051386 |
| TUBB3 | GC16P091061 |
| SLC34A2 | GC04P025657 |
| H3-3A | GC01P226062 |
| ATG7 | GC03P012072 |
| FGA | GC04M154583 |
| SBDS | GC07M066987 |
| CHD7 | GC08P060678 |
| LRP1B | GC02M140231 |
| EPHA4 | GC02M221418 |
| PTGES | GC09M129738 |
| OCA2 | GC15M027754 |
| NFIB | GC09M014077 |
| CD69 | GC12M019779 |
| PTPN6 | GC12P018571 |
| CHL1 | GC03P000213 |
| TIAM1 | GC21M031118 |
| S100A6 | GC01M153545 |
| SGK1 | GC06M134169 |
| XRCC4 | GC05P083077 |
| DAB2IP | GC09P121566 |
| PIEZO1 | GC16M088715 |
| FAP | GC02M162170 |
| HULC | GC06P008438 |
| NR5A1 | GC09M124481 |
| MECP2 | GC0XM154021 |
| MIR28 | GC03P188688 |
| CTCF | GC16P067563 |
| BANCR | GC09M069296 |
| VIP | GC06P152750 |
| HOXB9 | GC17M048621 |
| PLAT | GC08M042174 |
| HSPA1A | GC06P076883 |
| S100A1 | GC01P153627 |
| PLA2G1B | GC12M120322 |
| HBG2 | GC11M006236 |
| CXCL9 | GC04M076001 |
| CCN4 | GC08P133192 |
| KDM1A | GC01P023019 |
| COL14A1 | GC08P120096 |
| MIR212 | GC17M002050 |
| AKR1C1 | GC10P004963 |
| VRK1 | GC14P096797 |
| SNHG12 | GC01M028578 |
| TSHR | GC14P080954 |
| MIR211 | GC15M031065 |
| ADAM10 | GC15M058588 |
| PICSAR | GC21M050144 |
| PHOX2B | GC04M041746 |
| ANAPC1 | GC02M111611 |
| PSEN1 | GC14P073136 |
| PAX3 | GC02M222199 |
| AIMP2 | GC07P006016 |
| AOC3 | GC17P042851 |
| VDAC1 | GC05M133975 |
| MAD2L1 | GC04M120055 |
| CASK | GC0XM041514 |
| BLACAT1 | GC01M205389 |
| TNFSF12 | GC17P010605 |
| TRAF6 | GC11M036467 |
| TBX2 | GC17P061399 |
| MAPK7 | GC17P049488 |
| TP73-AS1 | GC01M005222 |
| LPAR1 | GC09M110873 |
| DAXX | GC06M033318 |
| CCAR1 | GC10P068721 |
| MTR | GC01P236795 |
| CDK7 | GC05P069332 |
| CD1A | GC01P158255 |
| AGO2 | GC08M140522 |
| CSNK2A1 | GC20M000472 |
| C4A | GC06P076888 |
| LINC01133 | GC01P159959 |
| MAP3K2 | GC02M127298 |
| CHRNB4 | GC15M079625 |
| PLCE1 | GC10P093993 |
| PIK3R2 | GC19P018153 |
| HDGF | GC01M156882 |
| CCR2 | GC03P046356 |
| CYP3A5 | GC07M099648 |
| AKR1B10 | GC07P134527 |
| MCM7 | GC07M100092 |
| S100A7 | GC01M153457 |
| AGT | GC01M230702 |
| LINC00673 | GC17M072290 |
| MYH11 | GC16M015704 |
| E2F5 | GC08P085177 |
| MIR29B2 | GC01M207806 |
| SCAI | GC09M124942 |
| NONO | GC0XP071255 |
| F5 | GC01M169511 |
| AAGAB | GC15M067200 |
| SF3B2 | GC11P066050 |
| TIA1 | GC02M070209 |
| TCERG1 | GC05P146447 |
| ORAI1 | GC12P125513 |
| PCAT29 | GC15P113116 |
| CCAR2 | GC08P022604 |
| MUC3A | GC07P100949 |
| FUT4 | GC11P094544 |
| EHBP1 | GC02P062673 |
| MBP | GC18M076978 |
| TOP2B | GC03M025598 |
| E2F4 | GC16P067192 |
| IFNAR1 | GC21P033324 |
| MIR133A1 | GC18M023352 |
| MIR148B | GC12P054337 |
| HNRNPA1 | GC12P054280 |
| CLPTM1L | GC05M001317 |
| MIR31HG | GC09M021439 |
| KL | GC13P033016 |
| VTCN1 | GC01M117143 |
| PLA2G4A | GC01P186798 |
| FGF3 | GC11M084878 |
| WWTR1 | GC03M149517 |
| PBX1 | GC01P164524 |
| DAB2 | GC05M039371 |
| AGTR1 | GC03P148697 |
| PSMB9 | GC06P076897 |
| SEMA4A | GC01P156147 |
| SNHG7 | GC09M137348 |
| LDLR | GC19P011091 |
| TP53BP2 | GC01M223779 |
| DELEC1 | GC09P118334 |
| SMARCA2 | GC09P001980 |
| FDPS | GC01P155308 |
| DCN | GC12M091140 |
| MAD2L2 | GC01M011674 |
| AMFR | GC16M056361 |
| RAD52 | GC12M000912 |
| TUBA1A | GC12M049184 |
| ARNT | GC01M150809 |
| OSM | GC22M030262 |
| PIP | GC07P143132 |
| RPL18 | GC19M048615 |
| MIR218-1 | GC04P020821 |
| CDC7 | GC01P091500 |
| SNCA | GC04M089724 |
| MICB | GC06P076856 |
| WNT3A | GC01P229075 |
| PCAT7 | GC09P094555 |
| GUSB | GC07M065960 |
| CXCL13 | GC04P077511 |
| SPN | GC16P029662 |
| LASP1 | GC17P038869 |
| RALBP1 | GC18P009465 |
| AQP1 | GC07P030911 |
| PLA2G6 | GC22M056043 |
| CDC45 | GC22P019479 |
| CD99 | GC0XP002691 |
| CTSC | GC11M088211 |
| HYAL2 | GC03M050317 |
| PPP1CB | GC02P028752 |
| HPRT1 | GC0XP134460 |
| AKAP13 | GC15P112287 |
| IGF2BP3 | GC07M023316 |
| SNCG | GC10P090641 |
| ADAM9 | GC08P038996 |
| BUB3 | GC10P123154 |
| MIR365A | GC16P014309 |
| TNFRSF10C | GC08P023102 |
| KAT5 | GC11P065711 |
| MIR4435-2HG | GC02M111019 |
| F2RL3 | GC19P016888 |
| PSAP | GC10M071816 |
| APOA1 | GC11M116835 |
| MUC2 | GC11P001074 |
| HIRA | GC22M019318 |
| TNFRSF4 | GC01M001211 |
| SSX2 | GC0XM052696 |
| IRF2BP2 | GC01M234604 |
| RPS15 | GC19P001438 |
| RHO | GC03P133387 |
| RAP1A | GC01P111542 |
| FLG | GC01M152274 |
| NRP2 | GC02P205681 |
| RBX1 | GC22P040951 |
| STXBP2 | GC19P007785 |
| NKILA | GC20P057711 |
| MIR135A1 | GC03M052296 |
| SART3 | GC12M108522 |
| SPI1 | GC11M084329 |
| ALOX15B | GC17P008039 |
| KDM5B | GC01M202696 |
| ACP3 | GC03P133444 |
| IKBKE | GC01P206470 |
| IGHMBP2 | GC11P068903 |
| ASAH1 | GC08M018055 |
| TP53BP1 | GC15M043403 |
| CLDN3 | GC07M073768 |
| SAA1 | GC11P018449 |
| REL | GC02P060881 |
| LIN28B | GC06P104949 |
| NOTCH4 | GC06M061333 |
| FOXA2 | GC20M022581 |
| PTH1R | GC03P046877 |
| HELLS | GC10P094501 |
| VAV1 | GC19P006772 |
| SMAD9 | GC13M036844 |
| PODXL | GC07M131500 |
| BACH2 | GC06M089926 |
| CDC25B | GC20P003890 |
| FSHR | GC02M048953 |
| HPS6 | GC10P102065 |
| LRP1 | GC12P057128 |
| HBA2 | GC16P009810 |
| TNFRSF9 | GC01M007915 |
| DNASE1 | GC16P003611 |
| ITGA11 | GC15M068296 |
| UNC13D | GC17M075827 |
| IQGAP1 | GC15P090388 |
| MED19 | GC11M057703 |
| PIK3C2A | GC11M017506 |
| FEN1 | GC11P061793 |
| ATP2A2 | GC12P110280 |
| RGCC | GC13P041457 |
| TBL1XR1 | GC03M177019 |
| HNRNPK | GC09M091384 |
| DLL1 | GC06M170282 |
| DNAJC21 | GC05P034929 |
| MAGT1 | GC0XM077913 |
| NECTIN1 | GC11M119624 |
| SHBG | GC17P007613 |
| PTN | GC07M137227 |
| RHEB | GC07M151466 |
| SALL4 | GC20M051784 |
| MIR340 | GC05M180015 |
| ARG1 | GC06P131473 |
| GPNMB | GC07P023238 |
| PANDAR | GC06M036673 |
| INHA | GC02P219569 |
| ACTA1 | GC01M229460 |
| IFIH1 | GC02M162267 |
| CASC15 | GC06P021669 |
| UBE3A | GC15M025333 |
| VCL | GC10P073995 |
| BCAR3 | GC01M093561 |
| MIR574 | GC04P038926 |
| PAK4 | GC19P039125 |
| IL33 | GC09P006350 |
| TUSC7 | GC03P116647 |
| IBSP | GC04P087799 |
| ROR2 | GC09M091805 |
| MB | GC22M035606 |
| GLB1 | GC03M032963 |
| ANO1 | GC11P069986 |
| SPINT2 | GC19P038244 |
| GACAT2 | GC18M008695 |
| STING1 | GC05M139476 |
| XDH | GC02M031334 |
| SRA1 | GC05M140537 |
| MDK | GC11P046380 |
| IGFBP1 | GC07P047504 |
| NHP2 | GC05M178149 |
| RPS27A | GC02P055231 |
| NOX4 | GC11M089324 |
| TPM3 | GC01M154127 |
| ATF1 | GC12P050763 |
| BST2 | GC19M017403 |
| NAT1 | GC08P018179 |
| MECOM | GC03M169083 |
| GJB6 | GC13M020221 |
| PVR | GC19P051984 |
| UBE2T | GC01M202332 |
| COL11A1 | GC01M102876 |
| PAX2 | GC10P100735 |
| NEK2 | GC01M211658 |
| NTS | GC12P085876 |
| CDA | GC01P020588 |
| NAB2 | GC12P057094 |
| ATF2 | GC02M175072 |
| PCLAF | GC15M079181 |
| MYOG | GC01M203083 |
| C3 | GC19M006677 |
| CXCL2 | GC04M074097 |
| SNHG15 | GC07M044983 |
| NCOA2 | GC08M070109 |
| NCR1 | GC19P054906 |
| FURIN | GC15P090868 |
| SERPINB2 | GC18P063871 |
| CXCL5 | GC04M073995 |
| KLF2 | GC19P050893 |
| CCK | GC03M042274 |
| WWP2 | GC16P069796 |
| SRPK1 | GC06M061442 |
| SULT1A1 | GC16M028650 |
| MIR455 | GC09P114209 |
| TGFBR3 | GC01M091680 |
| CASC11 | GC08M131051 |
| ST14 | GC11P130159 |
| DMTF1 | GC07P087155 |
| KNG1 | GC03P186717 |
| POLG | GC15M089475 |
| PRDM1 | GC06P105993 |
| CHAT | GC10P049609 |
| MIR184 | GC15P079209 |
| CDH23 | GC10P071396 |
| CALD1 | GC07P134744 |
| CCL18 | GC17P036064 |
| TBK1 | GC12P064451 |
| SNHG20 | GC17P077087 |
| MIR377 | GC14P109076 |
| MIR216A | GC02M055988 |
| PKP1 | GC01P201283 |
| HBA1 | GC16P009809 |
| NOVA1 | GC14M026443 |
| KLRB1 | GC12M019777 |
| E2F2 | GC01M023624 |
| CRKL | GC22P020917 |
| RPL27 | GC17P042998 |
| DSE | GC06P116255 |
| ZFP36 | GC19P039406 |
| CCL22 | GC16P057359 |
| NMB | GC15M084655 |
| ARID1B | GC06P156777 |
| MIR503 | GC0XM134684 |
| RIT1 | GC01M155897 |
| TACC3 | GC04P001723 |
| HLA-E | GC06P076836 |
| FOXK2 | GC17P082519 |
| SMARCAL1 | GC02P216412 |
| SBF2-AS1 | GC11P009758 |
| SMC1A | GC0XM053374 |
| PRNP | GC20P004686 |
| RBL1 | GC20M036996 |
| IGFBP6 | GC12P053097 |
| HIC1 | GC17P002054 |
| FABP4 | GC08M081478 |
| JUND | GC19M018279 |
| MIR124-3 | GC20P063270 |
| NEUROD1 | GC02M181673 |
| ELF3 | GC01P202007 |
| TARDBP | GC01P011013 |
| MIR326 | GC11M075335 |
| ATF3 | GC01P212565 |
| UHRF1 | GC19P004912 |
| ATP7A | GC0XP077979 |
| ADCY10 | GC01M167809 |
| BRMS1 | GC11M084739 |
| GLUL | GC01M182378 |
| CLCA2 | GC01P086424 |
| NMBR | GC06M142059 |
| MIR30C1 | GC01P040757 |
| SERPINH1 | GC11P075562 |
| GGT1 | GC22P024583 |
| MUC6 | GC11M001012 |
| SMAD6 | GC15P066702 |
| H1-0 | GC22P038270 |
| MAL | GC02P095025 |
| TTC7A | GC02P046906 |
| PSG2 | GC19M043064 |
| PCA3 | GC09P076691 |
| TRPS1 | GC08M115408 |
| USF2 | GC19P035268 |
| ETS2 | GC21P038805 |
| FOXL2 | GC03M138944 |
| POLB | GC08P042338 |
| AICDA | GC12M008602 |
| MIR151A | GC08M140733 |
| TBX21 | GC17P047733 |
| MIR135B | GC01M205448 |
| LLGL1 | GC17P018225 |
| PFN1 | GC17M004945 |
| CSF2RA | GC0XP001571 |
| PSEN2 | GC01P226870 |
| SOX11 | GC02P005703 |
| RBL2 | GC16P053433 |
| AKR1C3 | GC10P005035 |
| TBX5 | GC12M114353 |
| MAGEC2 | GC0XM142202 |
| GFI1 | GC01M092474 |
| ITGA2B | GC17M044797 |
| TPM1 | GC15P114623 |
| FCGR3B | GC01M161623 |
| IFNGR1 | GC06M137197 |
| API5 | GC11P043311 |
| RPS14 | GC05M150443 |
| SLFN11 | GC17M035350 |
| CDC37 | GC19M010391 |
| ZNRD1ASP | GC06M063204 |
| SERPINC1 | GC01M174490 |
| UBC | GC12M124911 |
| MIR361 | GC0XM085903 |
| ID2 | GC02P008678 |
| AQP3 | GC09M033431 |
| TNFRSF6B | GC20P063696 |
| ROCK2 | GC02M011289 |
| PON1 | GC07M095297 |
| LRP6 | GC12M019815 |
| SYNE1 | GC06M152121 |
| LATS1 | GC06M149658 |
| LINC00460 | GC13P106376 |
| TP53COR1 | GC06U903133 |
| FOXD2-AS1 | GC01M047432 |
| ACY1 | GC03P051983 |
| KLRC2 | GC12M019792 |
| EPB41L4A-DT | GC05P112421 |
| PLAG1 | GC08M056244 |
| NOP10 | GC15M034341 |
| PMAIP1 | GC18P059899 |
| PAX7 | GC01P018631 |
| PARK7 | GC01P008078 |
| ANG | GC14P030633 |
| NCOA1 | GC02P024492 |
| FBXO5 | GC06M152970 |
| HYAL1 | GC03M050299 |
| RPA1 | GC17P001829 |
| HAGLR | GC02M176173 |
| IL13RA2 | GC0XM115003 |
| CD58 | GC01M116514 |
| RPS7 | GC02P003575 |
| TGM1 | GC14M024249 |
| PRLR | GC05M035048 |
| INSM1 | GC20P020368 |
| ELP1 | GC09M108868 |
| CTAG1A | GC0XP154585 |
| FARSA | GC19M012922 |
| GHET1 | GC07P149181 |
| SCRIB | GC08M144074 |
| HES1 | GC03P194136 |
| RASA2 | GC03P141487 |
| COL1A2 | GC07P094394 |
| RPL31 | GC02P100985 |
| PBOV1 | GC06M138215 |
| LGALS9 | GC17P027629 |
| HCK | GC20P032052 |
| GHRL | GC03M010285 |
| PRKG1 | GC10P050991 |
| MACC1 | GC07M020140 |
| LIMK1 | GC07P074082 |
| CCNO | GC05M055231 |
| FPGS | GC09P127794 |
| CDK9 | GC09P127966 |
| DGCR5 | GC22P033239 |
| MIR378A | GC05P149732 |
| EEF1A1 | GC06M073515 |
| SCN5A | GC03M038549 |
| CYBA | GC16M088643 |
| DPYSL5 | GC02P026847 |
| IRAK1 | GC0XM154010 |
| MELK | GC09P036572 |
| SLAMF1 | GC01M160608 |
| MIR129-1 | GC07P128207 |
| BBC3 | GC19M047220 |
| STUB1 | GC16P009834 |
| SLC9A3R1 | GC17P074749 |
| CXCL14 | GC05M135617 |
| BTRC | GC10P101354 |
| SLC7A5 | GC16M087830 |
| CUL5 | GC11P108008 |
| CMA1 | GC14M024506 |
| CYP27B1 | GC12M057757 |
| KDM4B | GC19P004969 |
| MIR154 | GC14P109062 |
| SERPINF1 | GC17P001761 |
| KISS1 | GC01M204190 |
| TGFBI | GC05P136027 |
| PTENP1 | GC09M033673 |
| SSX1 | GC0XP050286 |
| TRD | GC14P030776 |
| SDF4 | GC01M001216 |
| PHLPP1 | GC18P062715 |
| CDC16 | GC13P114234 |
| KIR2DL1 | GC19P062547 |
| NR1H4 | GC12P100473 |
| MAP2 | GC02P209424 |
| DST | GC06M056457 |
| DSG1 | GC18P031318 |
| TAP2 | GC06M032821 |
| LNCRNA-ATB | GC14P031073 |
| HPS1 | GC10M098416 |
| NSD2 | GC04P001872 |
| MIR485 | GC14P109086 |
| NBAS | GC02M014998 |
| APBB1 | GC11M006396 |
| CYP24A1 | GC20M054153 |
| PDGFD | GC11M103907 |
| TUSC3 | GC08P015417 |
| SLC16A1 | GC01M112959 |
| SMN1 | GC05P070924 |
| DCUN1D1 | GC03M182938 |
| IL16 | GC15P081159 |
| BCL3 | GC19P044742 |
| GNAI2 | GC03P050226 |
| FCER2 | GC19M007689 |
| TAGLN | GC11P117199 |
| MYH7 | GC14M023412 |
| LYST | GC01M235661 |
| H4-16 | GC12M019858 |
| ACP1 | GC02P000293 |
| CGA | GC06M087085 |
| ELOC | GC08M073939 |
| CAGE1 | GC06M007326 |
| IFITM1 | GC11P000313 |
| SPDEF | GC06M061430 |
| MEIS1 | GC02P066433 |
| SET | GC09P128809 |
| GRB7 | GC17P050338 |
| PTH | GC11M013492 |
| ACKR3 | GC02P236537 |
| METTL13 | GC01P171781 |
| BNIP3 | GC10M131966 |
| SEPTIN9 | GC17P077282 |
| DNM1L | GC12P032679 |
| NOS1 | GC12M117208 |
| RAB27A | GC15M055202 |
| LY6K | GC08P142700 |
| CFH | GC01P196621 |
| SPTAN1 | GC09P128552 |
| WNT7A | GC03M019706 |
| TRAF2 | GC09P136881 |
| RPS6KA3 | GC0XM020149 |
| SETDB1 | GC01P150926 |
| PTK6 | GC20M063528 |
| ERN1 | GC17M064039 |
| MST1 | GC03M049683 |
| FLNC | GC07P128830 |
| GCG | GC02M162142 |
| TPD52 | GC08M079920 |
| HPS5 | GC11M018278 |
| PTGER2 | GC14P052314 |
| ITGA9 | GC03P037468 |
| AHRR | GC05P000321 |
| SLC2A2 | GC03M170996 |
| FOLR1 | GC11P072190 |
| BMX | GC0XP015392 |
| CACNA2D2 | GC03M050968 |
| DSG3 | GC18P031447 |
| TBX3 | GC12M114670 |
| MIR494 | GC14P109090 |
| MAP2K3 | GC17P049577 |
| MIR219A1 | GC06P033207 |
| PRMT1 | GC19P049675 |
| KLK10 | GC19M051012 |
| NOG | GC17P056593 |
| UMPS | GC03P124730 |
| SLC2A4 | GC17P010574 |
| KRT16 | GC17M041609 |
| PTPA | GC09P129111 |
| LIG1 | GC19M048115 |
| UBA7 | GC03M049805 |
| MAP3K5 | GC06M136557 |
| CIP2A | GC03M108545 |
| AGR2 | GC07M017014 |
| TAPBP | GC06M033299 |
| REN | GC01M204154 |
| CKS1B | GC01P154974 |
| LIMS1 | GC02P108534 |
| PROX1 | GC01P213983 |
| TFDP1 | GC13P113584 |
| IL18R1 | GC02P102311 |
| MFN2 | GC01P011980 |
| CYP11A1 | GC15M074337 |
| CCNL1 | GC03M157146 |
| SEMA4D | GC09M089360 |
| PERP | GC06M138088 |
| NHEJ1 | GC02M219111 |
| CACNA1C | GC12P001970 |
| IGFBP4 | GC17P040443 |
| TACSTD2 | GC01M058575 |
| COL4A1 | GC13M110148 |
| NR3C2 | GC04M148078 |
| HSP90B1 | GC12P103930 |
| HTR2A | GC13M046831 |
| RXRG | GC01M165401 |
| CD83 | GC06P014117 |
| MAP2K5 | GC15P092245 |
| MERTK | GC02P111898 |
| DPH1 | GC17P002030 |
| PER1 | GC17M010119 |
| SH3KBP1 | GC0XM019552 |
| CDH11 | GC16M064943 |
| TREM2 | GC06M061491 |
| USB1 | GC16P057999 |
| DNM2 | GC19P010718 |
| PRPF8 | GC17M001650 |
| IRF3 | GC19M049659 |
| TIGIT | GC03P114276 |
| MIR181B2 | GC09P124693 |
| AP3B1 | GC05M078000 |
| SLC3A2 | GC11P062856 |
| WNT2 | GC07M117297 |
| SEMA3A | GC07M083955 |
| F13A1 | GC06M006144 |
| HJURP | GC02M233834 |
| HPS3 | GC03P149129 |
| CORO1A | GC16P039395 |
| JUNB | GC19P012791 |
| CD163 | GC12M007646 |
| PTPRG | GC03P061561 |
| CFL1 | GC11M065823 |
| IGHE | GC14M112095 |
| SAMD9 | GC07M093099 |
| TH | GC11M002163 |
| ITCH | GC20P034363 |
| WNT10A | GC02P218892 |
| IL32 | GC16P009928 |
| IFI16 | GC01P158969 |
| SELENBP1 | GC01M151364 |
| PSENEN | GC19P051423 |
| ACE2 | GC0XM015494 |
| MIR33A | GC22P041900 |
| GRHL2 | GC08P101492 |
| PPARD | GC06P076988 |
| RETN | GC19P007669 |
| INHBA | GC07M041668 |
| CCNE2 | GC08M094879 |
| HLA-DRA | GC06P032439 |
| NR1I2 | GC03P119780 |
| MIR26B | GC02P218402 |
| RIOX1 | GC14P073492 |
| IRX2 | GC05M002708 |
| LGR5 | GC12P071439 |
| IL21R | GC16P027663 |
| RBBP4 | GC01P032651 |
| GAB1 | GC04P143336 |
| S1PR1 | GC01P101236 |
| PTPN2 | GC18M023298 |
| ADAMTS9-AS2 | GC03P064671 |
| EHMT1 | GC09P137618 |
| CRNDE | GC16M054845 |
| MAML2 | GC11M095976 |
| PTX3 | GC03P157436 |
| VAV3 | GC01M107571 |
| MIR339 | GC07M001022 |
| BRINP1 | GC09M119153 |
| IGFBP7 | GC04M057030 |
| ACTG1 | GC17M081509 |
| MAP3K7 | GC06M090513 |
| HMGCR | GC05P075336 |
| PRMT5 | GC14M022920 |
| CTNNA2 | GC02P079185 |
| KMT2C | GC07M152134 |
| MIR628 | GC15M055372 |
| VANGL1 | GC01P115641 |
| RAD21 | GC08M116846 |
| GPT | GC08P144502 |
| IGF2BP1 | GC17P048997 |
| CALM1 | GC14P090396 |
| WNT2B | GC01P112466 |
| GLA | GC0XM101393 |
| CD200 | GC03P112332 |
| ARPC1B | GC07P099374 |
| TMEFF2 | GC02M191950 |
| DNTT | GC10P096304 |
| LTF | GC03M046435 |
| EFEMP1 | GC02M055865 |
| ARRB1 | GC11M085031 |
| GJB5 | GC01P034755 |
| SULF1 | GC08P069466 |
| ECM1 | GC01P150508 |
| PPP6C | GC09M125147 |
| CCBE1 | GC18M059430 |
| ECRG4 | GC02P106063 |
| TFF3 | GC21M042311 |
| PIK3R3 | GC01M046041 |
| CRH | GC08M066176 |
| MIR92A1 | GC13P091535 |
| SRF | GC06P043171 |
| H3C2 | GC06M026032 |
| LAMA2 | GC06P128863 |
| FAT2 | GC05M151504 |
| PLCB1 | GC20P008061 |
| KRT15 | GC17M041513 |
| PDCD5 | GC19P032581 |
| COTL1 | GC16M084566 |
| SS18 | GC18M026016 |
| LIN28A | GC01P026410 |
| MIR425 | GC03M050864 |
| ARHGAP5 | GC14P032076 |
| UBE2K | GC04P039700 |
| MEFV | GC16M006424 |
| PTPRN | GC02M219289 |
| ABCB11 | GC02M168922 |
| PRNCR1 | GC08P127079 |
| CRYAB | GC11M111908 |
| NCF2 | GC01M183555 |
| PMEL | GC12M055954 |
| AGR3 | GC07M016854 |
| MADD | GC11P047290 |
| PRKCH | GC14P061187 |
| CYP2A13 | GC19P041088 |
| NPY | GC07P024290 |
| GNRHR | GC04M067737 |
| CRK | GC17M001420 |
| TJP2 | GC09P069121 |
| TNFRSF17 | GC16P011965 |
| MIR542 | GC0XM134638 |
| USP7 | GC16M008892 |
| SPRED2 | GC02M065307 |
| MIR345 | GC14P100307 |
| MIR95 | GC04M008007 |
| COL4A5 | GC0XP108439 |
| NR4A2 | GC02M156324 |
| FBLN1 | GC22P045502 |
| FGF9 | GC13P021671 |
| MIRLET7A2 | GC11M122146 |
| PDCD6 | GC05P000272 |
| LINC00261 | GC20M022547 |
| RBM38 | GC20P057391 |
| ABCB4 | GC07M087401 |
| PTP4A3 | GC08P141391 |
| NIPBL | GC05P036876 |
| CBS | GC21M043053 |
| PKD2 | GC04P088007 |
| SAG | GC02P233371 |
| ACACA | GC17M037084 |
| PRSS1 | GC07P147603 |
| H3C1 | GC06P076612 |
| FZD7 | GC02P202034 |
| SMARCAD1 | GC04P094207 |
| NPTN-IT1 | GC15M073566 |
| TCF21 | GC06P133889 |
| HSP90AB1 | GC06P044246 |
| MADCAM1 | GC19P002079 |
| HDAC3 | GC05M141583 |
| PLOD1 | GC01P011934 |
| DNAAF5 | GC07P000726 |
| MIR125B2 | GC21P016590 |
| ATXN2 | GC12M111443 |
| SAMHD1 | GC20M036890 |
| TRAF3 | GC14P108806 |
| MDC1 | GC06M061228 |
| APC2 | GC19P002288 |
| SLC6A3 | GC05M001392 |
| BIN1 | GC02M127048 |
| TEC | GC04M048137 |
| CSTA | GC03P122325 |
| MED1 | GC17M039404 |
| FANCB | GC0XM014690 |
| MIR370 | GC14P108753 |
| GHRH | GC20M037251 |
| CAD | GC02P027217 |
| GAS1 | GC09M086944 |
| KAT2B | GC03P020043 |
| CDH10 | GC05M024522 |
| FBLN5 | GC14M091869 |
| AGAP2-AS1 | GC12P057726 |
| SULF2 | GC20M047656 |
| SKIV2L | GC06P076887 |
| GMNN | GC06P024779 |
| CUX1 | GC07P101815 |
| CAV2 | GC07P116287 |
| CTC1 | GC17M010137 |
| YWHAZ | GC08M100917 |
| SOD3 | GC04P024798 |
| CNR1 | GC06M088139 |
| NNMT | GC11P114257 |
| NR0B1 | GC0XM030304 |
| ZEB2-AS1 | GC02P144519 |
| MIR625 | GC14P065471 |
| NPPB | GC01M011858 |
| NCOA6 | GC20M034754 |
| SPAG1 | GC08P100157 |
| SLIT2 | GC04P020287 |
| WNT10B | GC12M048965 |
| CTPS1 | GC01P040979 |
| RARG | GC12M053210 |
| RHOD | GC11P069262 |
| NEK8 | GC17P028725 |
| LINC00473 | GC06M165328 |
| MIR22HG | GC17M002550 |
| NPRL2 | GC03M050966 |
| HOXA9 | GC07M027162 |
| CTSG | GC14M024573 |
| DDIAS | GC11P082899 |
| AIRE | GC21P044285 |
| PLCB4 | GC20P009024 |
| ST6GAL1 | GC03P186930 |
| RICTOR | GC05M038937 |
| PRDM2 | GC01P013866 |
| TAC1 | GC07P097731 |
| BTG1 | GC12M092140 |
| GPSM2 | GC01P108875 |
| DKK3 | GC11M011962 |
| ALPL | GC01P021508 |
| SOX30 | GC05M157624 |
| FMR1 | GC0XP147933 |
| IVNS1ABP | GC01M185306 |
| SFRP2 | GC04M153780 |
| THRB | GC03M024117 |
| GSR | GC08M030678 |
| MIR198 | GC03M120395 |
| MIR708 | GC11M079402 |
| EMP2 | GC16M010541 |
| PRAME | GC22M022547 |
| IGF2-AS | GC11P002140 |
| HDAC5 | GC17M044076 |
| GIMAP6 | GC07M150625 |
| FLT3LG | GC19P052316 |
| MIR374A | GC0XM074296 |
| MAPK12 | GC22M055226 |
| IL17F | GC06M061661 |
| NUDT1 | GC07P002242 |
| PTP4A1 | GC06P077270 |
| EGLN1 | GC01M231363 |
| FZD4 | GC11M086945 |
| C20orf85 | GC20P058150 |
| GPR68 | GC14M091232 |
| HCST | GC19P051434 |
| MMP8 | GC11M102617 |
| FAT1 | GC04M186587 |
| DOCK2 | GC05P169637 |
| TFAP2C | GC20P056629 |
| LAMP2 | GC0XM120426 |
| CD164 | GC06M109366 |
| CCNB2 | GC15P059105 |
| EFNA1 | GC01P155127 |
| IL3RA | GC0XP001336 |
| PEG10 | GC07P094656 |
| CA2 | GC08P085463 |
| UBE2C | GC20P045812 |
| LAMA5 | GC20M062307 |
| FAT4 | GC04P125315 |
| PITX2 | GC04M110617 |
| WNT11 | GC11M076186 |
| CLDN18 | GC03P137998 |
| EPB41 | GC01P028887 |
| NOP2 | GC12M006556 |
| KCNMA1 | GC10M076869 |
| MIR218-2 | GC05M168768 |
| ADM | GC11P010304 |
| UPK2 | GC11P118925 |
| ELK1 | GC0XM047635 |
| NCOA4 | GC10M046005 |
| DRD2 | GC11M113409 |
| FES | GC15P090883 |
| CACNA1G | GC17P052034 |
| CASP6 | GC04M109688 |
| IL23A | GC12P057101 |
| APOD | GC03M195568 |
| FHL2 | GC02M105357 |
| CAMP | GC03P048674 |
| MPLKIP | GC07M040126 |
| MIR615 | GC12P054033 |
| CHRM3 | GC01P239386 |
| ATF4 | GC22P039599 |
| ABCC4 | GC13M095019 |
| LGALS3BP | GC17M078971 |
| PRDX2 | GC19M012796 |
| VIPR1 | GC03P042490 |
| ALPG | GC02P232407 |
| CYP26A1 | GC10P093073 |
| DMRT1 | GC09P000831 |
| CAPRIN1 | GC11P034051 |
| IL23R | GC01P067138 |
| LAG3 | GC12P018557 |
| ID3 | GC01M023557 |
| LAMP1 | GC13P113297 |
| INTS6 | GC13M051354 |
| GSDME | GC07M024699 |
| IL10RA | GC11P117987 |
| ZNF793 | GC19P037506 |
| ECE1 | GC01M021217 |
| SOX17 | GC08P054457 |
| COPS5 | GC08M067043 |
| SPAAR | GC09P039497 |
| KRT4 | GC12M052806 |
| CTBP1 | GC04M001211 |
| NUMB | GC14M073275 |
| WFDC2 | GC20P045469 |
| DCLK1 | GC13M035768 |
| MIRLET7F1 | GC09P094257 |
| RBPJ | GC04P026165 |
| RFX5 | GC01M151340 |
| SLC40A1 | GC02M189560 |
| ATAD2 | GC08M123319 |
| RGMB-AS1 | GC05M098769 |
| SATB1 | GC03M019799 |
| AVP | GC20M003082 |
| UNG | GC12P109097 |
| MAP1B | GC05P072107 |
| NUAK1 | GC12M106063 |
| ACHE | GC07M100889 |
| IL1RL1 | GC02P102294 |
| DSG2 | GC18P031498 |
| PBK | GC08M027809 |
| DEFB1 | GC08M006870 |
| ACD | GC16M067658 |
| SIRPA | GC20P001894 |
| NORAD | GC20M036214 |
| INPPL1 | GC11P072223 |
| COL5A1 | GC09P134641 |
| ACTN1 | GC14M068874 |
| DHCR7 | GC11M071428 |
| CDCA5 | GC11M084652 |
| JMJD1C | GC10M063167 |
| PF4 | GC04M073980 |
| SERPINB1 | GC06M002833 |
| KDM6A | GC0XP044873 |
| ENAH | GC01M225486 |
| ZEB1-AS1 | GC10M031166 |
| CBR3-AS1 | GC21M036131 |
| HNRNPH1 | GC05M179614 |
| METTL3 | GC14M021498 |
| ATG5 | GC06M106045 |
| MVD | GC16M088651 |
| DSC3 | GC18M030990 |
| CKB | GC14M103519 |
| YWHAQ | GC02M009583 |
| TPM2 | GC09M035672 |
| NKX3-1 | GC08M023678 |
| RPS6 | GC09M019375 |
| PTBP1 | GC19P000797 |
| GGPS1 | GC01P235327 |
| STX11 | GC06P144169 |
| AQP5 | GC12P049961 |
| NCL | GC02M231453 |
| TRPV3 | GC17M003986 |
| IFNAR2 | GC21P033229 |
| SDCCAG8 | GC01P243255 |
| GREB1 | GC02P011482 |
| AKAP12 | GC06P151239 |
| PTPRU | GC01P029236 |
| DVL1 | GC01M001335 |
| CANX | GC05P179678 |
| MPDZ | GC09M013095 |
| URI1 | GC19P029923 |
| CBLB | GC03M105655 |
| STAG2 | GC0XP123960 |
| PRRC2A | GC06P076867 |
| ATP7B | GC13M051930 |
| ADAMTSL1 | GC09P017906 |
| TERF2 | GC16M069355 |
| ZFP36L1 | GC14M068787 |
| CHKA | GC11M068052 |
| NFATC3 | GC16P068119 |
| BRS3 | GC0XP136482 |
| DDX58 | GC09M032455 |
| SATB2 | GC02M199269 |
| PPIEL | GC01M039522 |
| TARS1 | GC05P033441 |
| MAPK8IP1 | GC11P046399 |
| PELP1 | GC17M004669 |
| ZFPM2 | GC08P104590 |
| EHMT2 | GC06M031879 |
| CD74 | GC05M150378 |
| MUC20 | GC03P195720 |
| CDH17 | GC08M094127 |
| DNAH11 | GC07P021543 |
| MIR409 | GC14P109082 |
| DYRK1A | GC21P037365 |
| FEZF1-AS1 | GC07P122303 |
| KIF11 | GC10P092574 |
| GTF2H5 | GC06P158168 |
| PROS1 | GC03M093873 |
| JAM3 | GC11P134068 |
| HK1 | GC10P069269 |
| GP5 | GC03M194395 |
| EIF4G1 | GC03P184314 |
| LGALS7 | GC19M038770 |
| NTN1 | GC17P009021 |
| ANGPTL4 | GC19P008363 |
| RBP4 | GC10M093591 |
| DHDH | GC19P052268 |
| BTG2 | GC01P203305 |
| NFKBIL1 | GC06P076858 |
| EIF3H | GC08M116642 |
| RNASE3 | GC14P020891 |
| ANK1 | GC08M041653 |
| ERCC8 | GC05M060888 |
| MTRR | GC05P007851 |
| HPN | GC19P035040 |
| RTN4 | GC02M054934 |
| H3C11 | GC06M061830 |
| CD109 | GC06P073695 |
| CTAGE1 | GC18M022413 |
| PCBP2-OT1 | GC12P053464 |
| AFAP1 | GC04M007758 |
| COX5A | GC15M074919 |
| RAD9A | GC11P069283 |
| B3GAT1 | GC11M134378 |
| NTF3 | GC12P005432 |
| S100P | GC04P006700 |
| TNFSF13 | GC17P007558 |
| TPI1 | GC12P018562 |
| LDB1 | GC10M102106 |
| SUZ12 | GC17P031937 |
| CENPF | GC01P214603 |
| NUDC | GC01P027286 |
| EGLN3 | GC14M033924 |
| GRK2 | GC11P067266 |
| TMPRSS6 | GC22M037066 |
| MGAT5 | GC02P134119 |
| SMC3 | GC10P110567 |
| SWAP70 | GC11P009664 |
| VCAN | GC05P083471 |
| MYBL2 | GC20P043667 |
| TPM4 | GC19P050886 |
| PSMB5 | GC14M023016 |
| COL4A2 | GC13P110305 |
| PDE4A | GC19P010416 |
| TTC12 | GC11P113314 |
| PTPRS | GC19M005157 |
| HSPA9 | GC05M138554 |
| SAFB | GC19P005623 |
| FUT2 | GC19P048695 |
| PRC1 | GC15M090966 |
| GPX3 | GC05P150997 |
| IL17RA | GC22P018163 |
| TSR2 | GC0XP054441 |
| MIR92A2 | GC0XM134206 |
| CD1C | GC01P158289 |
| FUZ | GC19M049806 |
| SCARB1 | GC12M124776 |
| HLA-DRB5 | GC06M061346 |
| LOXL4 | GC10M098247 |
| HTATIP2 | GC11P020363 |
| BRD2 | GC06P076900 |
| TREX1 | GC03P048695 |
| THBS4 | GC05P079991 |
| UPK3A | GC22P045284 |
| HOXA5 | GC07M027482 |
| TUBA4B | GC02P219253 |
| MUS81 | GC11P069021 |
| TOR1A | GC09M129812 |
| MAF | GC16M079204 |
| PSMC4 | GC19P051655 |
| BIVM-ERCC5 | GC13P102813 |
| BPTF | GC17P067825 |
| DNASE1L3 | GC03M058192 |
| ACVR1 | GC02M157736 |
| ITPR3 | GC06P033620 |
| CCDC39 | GC03M180602 |
| INPP5D | GC02P233059 |
| TNIP1 | GC05M151029 |
| SLC2A3 | GC12M007919 |
| MIR582 | GC05M059703 |
| LINC00858 | GC10P090606 |
| AGK | GC07P141551 |
| AGRN | GC01P001020 |
| P4HA2 | GC05M132191 |
| BLOC1S5 | GC06M008014 |
| RAB25 | GC01P156061 |
| EPHB6 | GC07P147625 |
| TRPC6 | GC11M101451 |
| KLK6 | GC19M050958 |
| KCNJ5 | GC11P128891 |
| KCNH1 | GC01M210678 |
| PHLDA1 | GC12M076025 |
| NR4A1 | GC12P052022 |
| LIG3 | GC17P034980 |
| MIR498 | GC19P062491 |
| LRIG1 | GC03M066379 |
| HOXA10 | GC07M027490 |
| YES1 | GC18M000721 |
| PIEZO2 | GC18M010670 |
| PFKFB3 | GC10P006144 |
| LINC00312 | GC03P008571 |
| LATS2 | GC13M020973 |
| FST | GC05P053480 |
| NEK10 | GC03M027128 |
| CDK8 | GC13P026254 |
| POLR2A | GC17P010603 |
| HIF1A-AS1 | GC14M061681 |
| TREM1 | GC06M041267 |
| ITGA7 | GC12M055684 |
| TYROBP | GC19M035904 |
| TRPM8 | GC02P233917 |
| PFAS | GC17P008247 |
| KLK4 | GC19M062299 |
| COL4A3 | GC02P227164 |
| MYO5A | GC15M080007 |
| FZD1 | GC07P091264 |
| CSE1L | GC20P049046 |
| KIF5B | GC10M032526 |
| PGK1 | GC0XP077980 |
| FALEC | GC01P150534 |
| NCSTN | GC01P160343 |
| IGF2BP2 | GC03M185643 |
| NFAT5 | GC16P069565 |
| HLTF | GC03M149030 |
| OPRM1 | GC06P154075 |
| ISL1 | GC05P051383 |
| UROS | GC10M125784 |
| ZNF461 | GC19M061820 |
| ASNS | GC07M097854 |
| CD207 | GC02M070830 |
| PHGDH | GC01P119660 |
| RBM10 | GC0XP047418 |
| DMPK | GC19M045769 |
| TYRP1 | GC09P012683 |
| IQANK1 | GC08P144067 |
| LINC01116 | GC02M176629 |
| SSTR1 | GC14P038207 |
| SCT | GC11M000626 |
| GTF2E2 | GC08M030578 |
| AKAP9 | GC07P091940 |
| PSAT1 | GC09P078297 |
| BCL9 | GC01P147541 |
| LRRC8A | GC09P128882 |
| TPP2 | GC13P102596 |
| FOXC2 | GC16P086577 |
| FAM168A | GC11M073400 |
| C5AR1 | GC19P047290 |
| RSPH1 | GC21M042472 |
| ITGB6 | GC02M160099 |
| CSNK2B | GC06P076868 |
| GJB1 | GC0XP071212 |
| CILK1 | GC06M061695 |
| NME2 | GC17P051165 |
| CXCR6 | GC03P046271 |
| SLC45A3 | GC01M205626 |
| EPIST | GC05M135099 |
| HLA-DQA2 | GC06P032741 |
| GSDMB | GC17M039904 |
| LY6D | GC08M142784 |
| HLA-DMA | GC06M061371 |
| FHL1 | GC0XP136146 |
| EFNB2 | GC13M106489 |
| SCARB2 | GC04M076158 |
| FTH1 | GC11M061959 |
| AHCY | GC20M034468 |
| TXNRD2 | GC22M019863 |
| TPBG | GC06P082363 |
| FKBP5 | GC06M061437 |
| ATOH1 | GC04P093828 |
| FUT3 | GC19M005843 |
| CX3CL1 | GC16P057372 |
| PTK7 | GC06P043076 |
| DEK | GC06M018224 |
| DACT1 | GC14P058633 |
| SLC7A11 | GC04M138164 |
| MAP3K20 | GC02P173076 |
| DLGAP2 | GC08P000739 |
| SCHLAP1 | GC02P180511 |
| LTBP4 | GC19P040592 |
| H3-3B | GC17M075805 |
| REV3L | GC06M111299 |
| PPIA | GC07P044807 |
| BAG6 | GC06M031639 |
| FZD6 | GC08P103298 |
| MUC13 | GC03M124905 |
| TP53TG1 | GC07M087325 |
| SKI | GC01P002228 |
| SLC17A5 | GC06M073593 |
| ELMO3 | GC16P067199 |
| DLEU1 | GC13P050117 |
| TGFB1I1 | GC16P039498 |
| FERMT2 | GC14M052857 |
| PLEK | GC02P068365 |
| SRD5A1 | GC05P006633 |
| PXDN | GC02M001635 |
| IL22 | GC12M068248 |
| SNHG5 | GC06M085650 |
| DDB1 | GC11M084500 |
| EIF2AK3 | GC02M088556 |
| IL15RA | GC10M005943 |
| NCR3 | GC06M031588 |
| KIR2DS4 | GC19P062552 |
| LACTB | GC15P112676 |
| LECT2 | GC05M135922 |
| TATDN1 | GC08M124488 |
| NUP98 | GC11M003671 |
| RHOBTB2 | GC08P022987 |
| SHMT1 | GC17M024669 |
| KRIT1 | GC07M092198 |
| G6PC1 | GC17P051152 |
| TLR8 | GC0XP012924 |
| ATP12A | GC13P024680 |
| RDX | GC11M109864 |
| RPA2 | GC01M027904 |
| PRSS8 | GC16M035913 |
| SLC17A9 | GC20P062952 |
| ID4 | GC06P019837 |
| PIAS1 | GC15P068054 |
| TYRO3 | GC15P041557 |
| FBL | GC19M039834 |
| LAMB1 | GC07M107923 |
| MAP3K14 | GC17M045263 |
| RAP1B | GC12P068610 |
| CD1B | GC01M158297 |
| SLC52A3 | GC20M000741 |
| SFTA1P | GC10M010784 |
| CPOX | GC03M098576 |
| NPPA | GC01M011846 |
| MCM5 | GC22P035400 |
| IRAIN | GC15M098645 |
| OCLN | GC05P069492 |
| CDK11A | GC01M001702 |
| CLDN5 | GC22M019523 |
| CUL4A | GC13P113208 |
| RAB11A | GC15P092241 |
| DSC2 | GC18M031058 |
| MATR3 | GC05P139274 |
| AFDN | GC06P167827 |
| OSMR | GC05P038845 |
| TACR1 | GC02M075047 |
| GART | GC21M033503 |
| CST6 | GC11P069036 |
| CDK2AP1 | GC12M123250 |
| TCOF1 | GC05P150358 |
| GP6 | GC19M055013 |
| PLD2 | GC17P004808 |
| DAG1 | GC03P049540 |
| LGALS4 | GC19M061834 |
| TAF15 | GC17P049975 |
| GAS6 | GC13M113820 |
| SIX3 | GC02P044941 |
| PLAGL1 | GC06M143940 |
| ACTR3 | GC02P113889 |
| ITPR1 | GC03P004486 |
| LOC110806306 | GC03P169764 |
| SDCBP | GC08P058539 |
| PYCARD | GC16M031201 |
| ATP4A | GC19M062663 |
| DEFB4A | GC08P007895 |
| KHDRBS1 | GC01P032013 |
| PATJ | GC01P061743 |
| SDC2 | GC08P096495 |
| NCK1 | GC03P136862 |
| ASCC1 | GC10M072096 |
| GAS6-AS1 | GC13P113815 |
| PRKD3 | GC02M037251 |
| LTBP1 | GC02P033060 |
| C4B | GC06P032014 |
| COL5A2 | GC02M189031 |
| PPP1R13L | GC19M045379 |
| RALA | GC07P039622 |
| LPAR3 | GC01M084811 |
| DLL4 | GC15P040929 |
| KIR2DS1 | GC19Mr00063 |
| ESRRA | GC11P064305 |
| LIPC | GC15P058410 |
| TOLLIP | GC11M001274 |
| RAD17 | GC05P069369 |
| ALAD | GC09M113386 |
| LAMC1 | GC01P182992 |
| CDX1 | GC05P150166 |
| TNS4 | GC17M040475 |
| MSI1 | GC12M120341 |
| PLIN2 | GC09M019159 |
| UBE2I | GC16P009841 |
| SLC12A2 | GC05P128083 |
| TRPM2-AS | GC21M044414 |
| RNY1 | GC07M148987 |
| MRC2 | GC17P062627 |
| LRP2 | GC02M169127 |
| ELMO2 | GC20M046366 |
| ENTPD1 | GC10P095711 |
| SGO1-AS1 | GC03P020174 |
| CTNND2 | GC05M010971 |
| RHAG | GC06M049605 |
| LEFTY2 | GC01M225937 |
| TNFRSF25 | GC01M006460 |
| NID1 | GC01M235975 |
| WNT6 | GC02P218859 |
| MCRS1 | GC12M049557 |
| POU5F1B | GC08P127322 |
| FECH | GC18M057544 |
| CLCA4 | GC01P086547 |
| TSPO | GC22P043151 |
| CGB5 | GC19P049043 |
| GALNS | GC16M088813 |
| BUB1B-PAK6 | GC15P040219 |
| RPS6KA2 | GC06M166409 |
| METRNL | GC17P083079 |
| PSIP1 | GC09M015464 |
| ICAM2 | GC17M064002 |
| MAL2 | GC08P119165 |
| GAD1 | GC02P170813 |
| GAL | GC11P069367 |
| SLC16A1-AS1 | GC01P112956 |
| RNY3 | GC07P149006 |
| ZNF217 | GC20M053567 |
| CDK3 | GC17P076008 |
| HAND2-AS1 | GC04P173527 |
| GNL3 | GC03P052681 |
| IAPP | GC12P021354 |
| RAD23B | GC09P107283 |
| MAPKAPK2 | GC01P206684 |
| SLC6A4 | GC17M030194 |
| USP9X | GC0XP041085 |
| TTN-AS1 | GC02P178521 |
| BLCAP | GC20M037492 |
| HHAT | GC01P210328 |
| AKR1C2 | GC10M004987 |
| SIRT2 | GC19M038878 |
| HAX1 | GC01P154273 |
| WNK1 | GC12P000733 |
| AHI1 | GC06M135283 |
| HNRNPU | GC01M244844 |
| ABCA4 | GC01M093992 |
| HDC | GC15M050241 |
| SIN3A | GC15M075369 |
| HOXB2 | GC17M048540 |
| CADM2 | GC03P085008 |
| MUC20-OT1 | GC03P196156 |
| LAMA1 | GC18M006941 |
| CRTC1 | GC19P050956 |
| PSTPIP1 | GC15P076993 |
| THBS2 | GC06M169215 |
| PLD1 | GC03M171600 |
| SIGLEC5 | GC19M062340 |
| CLOCK | GC04M055427 |
| CEACAM4 | GC19M061904 |
| DOT1L | GC19P002189 |
| POU2F1 | GC01P167190 |
| CHRNA7 | GC15P031923 |
| DLG1 | GC03M197042 |
| RPTOR | GC17P080544 |
| RELN | GC07M103471 |
| LRATD2 | GC08M131164 |
| SPTLC1 | GC09M092007 |
| CGB3 | GC19M062175 |
| RAN | GC12P130871 |
| PRKACB | GC01P084078 |
| PAX1 | GC20P021705 |
| PSMB4 | GC01P151372 |
| MIR675 | GC11M002728 |
| TOP3A | GC17M024647 |
| PDLIM2 | GC08P022578 |
| HHIP | GC04P144645 |
| KIF20B | GC10P089701 |
| EIF3A | GC10M119034 |
| NUS1 | GC06P117675 |
| ALOX15 | GC17M004630 |
| RPS3 | GC11P077447 |
| CSPG4 | GC15M075674 |
| CXCL11 | GC04M076033 |
| TUBA1B | GC12M049127 |
| CCL19 | GC09M034692 |
| GAS5-AS1 | GC01P173863 |
| PRDX3 | GC10M119167 |
| BAG3 | GC10P119651 |
| BMP1 | GC08P022164 |
| PKP2 | GC12M032790 |
| LMNB1 | GC05P126776 |
| EIF3M | GC11P032584 |
| CUL2 | GC10M035046 |
| GAB2 | GC11M078215 |
| TMSB4X | GC0XP012975 |
| RBBP6 | GC16P024537 |
| GNA13 | GC17M065009 |
| OLA1 | GC02M174072 |
| MYH10 | GC17M008474 |
| ATF6 | GC01P161766 |
| P4HB | GC17M081843 |
| SOX5 | GC12M023529 |
| SPIN1 | GC09P088388 |
| HYOU1 | GC11M119156 |
| LINC00857 | GC10P090582 |
| CDCA2 | GC08P025458 |
| RNF113A | GC0XM119870 |
| RXRB | GC06M033193 |
| EPS8 | GC12M019870 |
| ADAM15 | GC01P155050 |
| MIR495 | GC14P109091 |
| PRDM16 | GC01P003437 |
| FAM3C | GC07M121349 |
| ARF6 | GC14P049895 |
| SPON2 | GC04M001166 |
| MAPRE1 | GC20P032819 |
| POLR1H | GC06P079647 |
| LINC01589 | GC22M055147 |
| WDR1 | GC04M010075 |
| SLC9A1 | GC01M027109 |
| TBXT | GC06M166158 |
| F11R | GC01M160995 |
| UGT1A6 | GC02P233691 |
| CLIC1 | GC06M061314 |
| MBD4 | GC03M129430 |
| PHLPP2 | GC16M071637 |
| MIR134 | GC14P109061 |
| DUSP6 | GC12M089347 |
| GZMA | GC05P055102 |
| MAP1LC3A | GC20P034546 |
| ENPEP | GC04P110365 |
| MIR637 | GC19M003961 |
| RSPO2 | GC08M107899 |
| GNAS-AS1 | GC20M058811 |
| KCNH2 | GC07M150944 |
| LAMTOR2 | GC01P156054 |
| PMEPA1 | GC20M057648 |
| GPC1 | GC02P240435 |
| CDCA4 | GC14M105009 |
| PBXIP1 | GC01M154944 |
| BIRC6 | GC02P032484 |
| ANXA3 | GC04P078551 |
| RSF1 | GC11M085116 |
| MYBL1 | GC08M066562 |
| PPP1CA | GC11M084788 |
| GC | GC04M071741 |
| LUC7L2 | GC07P139344 |
| CHFR | GC12M132822 |
| SERPINE2 | GC02M223975 |
| MFAP5 | GC12M008637 |
| GAD2 | GC10P026216 |
| C14orf132 | GC14P096040 |
| WDR77 | GC01M111439 |
| XAGE1A | GC0XP052496 |
| RAC3 | GC17P082031 |
| LTO1 | GC11M084871 |
| WNT5B | GC12P001529 |
| P2RY1 | GC03P152835 |
| CSTB | GC21M043772 |
| TRPV1 | GC17M003565 |
| CDR1-AS | GC0XU902169 |
| VSIR | GC10M071748 |
| GPR87 | GC03M151294 |
| IHH | GC02M219054 |
| FLVCR1 | GC01P212858 |
| AP3D1 | GC19M004434 |
| FTO | GC16P053787 |
| MIR873 | GC09M028880 |
| TIE1 | GC01P043300 |
| TNK2 | GC03M195863 |
| PAK2 | GC03P196739 |
| ILF3 | GC19P010655 |
| MNDA | GC01P158801 |
| HECA | GC06P139135 |
| FAM107A | GC03M058714 |
| ISG15 | GC01P001001 |
| ITGB5 | GC03M124761 |
| SPTBN1 | GC02P054456 |
| TRIM21 | GC11M004384 |
| TMPRSS11A | GC04M067909 |
| LINC01433 | GC20P004193 |
| FBP1 | GC09M094603 |
| MYO6 | GC06P075749 |
| TEAD1 | GC11P012674 |
| EPG5 | GC18M045800 |
| BCL7B | GC07M073536 |
| KLK7 | GC19M062303 |
| STK33 | GC11M008335 |
| CCL21 | GC09M034709 |
| EVPL | GC17M076004 |
| CSNK2A2 | GC16M058157 |
| S100A14 | GC01M153614 |
| MYEF2 | GC15M048134 |
| STK19 | GC06P031971 |
| SETD1A | GC16P039459 |
| ADGRG1 | GC16P057610 |
| BCL2L2 | GC14P030949 |
| POR | GC07P075899 |
| PHB2 | GC12M006965 |
| PMP22 | GC17M015229 |
| UPF1 | GC19P018831 |
| GRIN2B | GC12M013437 |
| PVALB | GC22M036800 |
| PPBP | GC04M073986 |
| UGCG | GC09P111896 |
| PLK4 | GC04P127880 |
| POGZ | GC01M151402 |
| BCL2A1 | GC15M080585 |
| CYP2C9 | GC10P094938 |
| SLC22A2 | GC06M160185 |
| CAMK2G | GC10M073812 |
| PRDX6 | GC01P173477 |
| LPXN | GC11M084411 |
| PDGFC | GC04M156760 |
| AKIP1 | GC11P008911 |
| FBXO11 | GC02M047789 |
| STC1 | GC08M023841 |
| PFKM | GC12P048105 |
| NACC1 | GC19P013579 |
| FZD8 | GC10M035638 |
| KLF10 | GC08M102648 |
| CACNA1G-AS1 | GC17M050772 |
| KDM6B | GC17P007834 |
| LINC00880 | GC03M157081 |
| FGR | GC01M027706 |
| SKP1 | GC05M134148 |
| ASAP1-IT1 | GC08M130295 |
| TCP1 | GC06M159778 |
| C5 | GC09M120952 |
| XAGE1B | GC0XM052513 |
| SPEN | GC01P015848 |
| PCAT2 | GC08M127072 |
| CYB5R3 | GC22M055106 |
| CRABP1 | GC15P078340 |
| TWIST2 | GC02P238848 |
| RPL6 | GC12M112320 |
| CYGB | GC17M076527 |
| SRSF1 | GC17M058000 |
| TRPV6 | GC07M142871 |
| ICAM5 | GC19P010289 |
| JAG2 | GC14M105140 |
| PIGR | GC01M206928 |
| MCM6 | GC02M135839 |
| POLI | GC18P054274 |
| RMI2 | GC16P011250 |
| SP3 | GC02M173882 |
| RAB5A | GC03P019948 |
| NOX1 | GC0XM100843 |
| HSPB2 | GC11P111913 |
| CDCA3 | GC12M006844 |
| HOXA-AS2 | GC07P027107 |
| S100A11 | GC01M152032 |
| ERAP1 | GC05M096760 |
| CST3 | GC20M023646 |
| PPP2CA | GC05M134194 |
| NEFL | GC08M024950 |
| CSNK1A1 | GC05M149492 |
| EIF3D | GC22M036510 |
| APTX | GC09M032886 |
| MBTPS2 | GC0XP021839 |
| SPOP | GC17M049598 |
| RBP1 | GC03M139517 |
| HTRA1 | GC10P122461 |
| RPE65 | GC01M068428 |
| DDC | GC07M050458 |
| KIR2DL2 | GC19Mr00108 |
| MIR202 | GC10M133247 |
| HSPH1 | GC13M031134 |
| FERMT3 | GC11P064286 |
| SOCS2 | GC12P093569 |
| AFF4 | GC05M132875 |
| SEMA3C | GC07M080742 |
| TRRAP | GC07P098877 |
| SPINT1 | GC15P040844 |
| AURKC | GC19P057230 |
| GPX4 | GC19P001103 |
| PRKAB1 | GC12P119632 |
| LINC01852 | GC15M038062 |
| LAMA4 | GC06M112107 |
| AMBP | GC09M114060 |
| GSTA1 | GC06M052791 |
| ANLN | GC07P036389 |
| MIR490 | GC07P136903 |
| COL6A3 | GC02M237324 |
| GSS | GC20M034928 |
| CYSLTR1 | GC0XM078271 |
| ATRIP | GC03P048688 |
| HDAC8 | GC0XM072329 |
| SMAD1 | GC04P145481 |
| PRPF31 | GC19P062519 |
| TMPO | GC12P098515 |
| MSI2 | GC17P057255 |
| SCGB3A1 | GC05M180590 |
| PEA15 | GC01P160205 |
| CCL26 | GC07M075769 |
| TXNRD1 | GC12P104215 |
| EPRS1 | GC01M219969 |
| LOC730101 | GC06P052664 |
| MIR337 | GC14P109070 |
| GRIN2A | GC16M009753 |
| TNFRSF19 | GC13P023570 |
| CPS1-IT1 | GC02P210617 |
| LPCAT1 | GC05M001456 |
| MTSS1 | GC08M124550 |
| NUP88 | GC17M005997 |
| PIAS3 | GC01M145848 |
| LTBP3 | GC11M065538 |
| HAS2 | GC08M121594 |
| ZFP36L2 | GC02M043184 |
| PRODH | GC22M018912 |
| HMBS | GC11P119084 |
| ATP1B1 | GC01P169105 |
| SCGB2A2 | GC11P062269 |
| SUMO1 | GC02M202206 |
| HEIH | GC05M181175 |
| SCAP | GC03M047413 |
| RARRES2 | GC07M150333 |
| GPI | GC19P051335 |
| ABI1 | GC10M026746 |
| NMRAL2P | GC03P185961 |
| NKX2-8 | GC14M036580 |
| TUBB2A | GC06M003153 |
| UGT1A7 | GC02P233681 |
| PI3 | GC20P045174 |
| BMPR1B | GC04P094757 |
| KIF15 | GC03P046241 |
| CDC42BPB | GC14M102932 |
| PENK | GC08M056436 |
| EMD | GC0XP154379 |
| AIF1 | GC06P076863 |
| PCAT19 | GC19M061899 |
| NABP1 | GC02P191678 |
| ESRRG | GC01M216503 |
| GLS | GC02P190880 |
| PIK3R4 | GC03M130678 |
| QKI | GC06P163414 |
| RYBP | GC03M072371 |
| VEGFB | GC11P064234 |
| EOMES | GC03M027715 |
| NR2F2 | GC15P096325 |
| AKT1S1 | GC19M049869 |
| ABCC6 | GC16M016148 |
| MIA2 | GC14P039230 |
| SPA17 | GC11P124673 |
| CAMK2N1 | GC01M020482 |
| ARTN | GC01P043933 |
| THBS3 | GC01M155195 |
| KIR3DS1 | GC19MR00058 |
| UROD | GC01P045270 |
| DHX9 | GC01P182839 |
| PDE5A | GC04M119494 |
| DCT | GC13M094436 |
| RASSF2 | GC20M004780 |
| LINC00968 | GC08M056496 |
| CTHRC1 | GC08P103371 |
| MYBPC3 | GC11M084328 |
| GLIPR1 | GC12P075480 |
| CCNT1 | GC12M048688 |
| UCP2 | GC11M073974 |
| SLC1A2 | GC11M035256 |
| MIR199A2 | GC01M172235 |
| SMARCD2 | GC17M063832 |
| TUBG1 | GC17P042609 |
| GUCY1B2 | GC13M050994 |
| OGFR | GC20P062804 |
| MUC7 | GC04P070430 |
| MMP16 | GC08M088032 |
| ADNP | GC20M050888 |
| MAFA-AS1 | GC08P143417 |
| MDH2 | GC07P076048 |
| BHLHE40 | GC03P004980 |
| ANO7 | GC02P241188 |
| HOXA13 | GC07M027494 |
| GP1BA | GC17P004932 |
| RASSF5 | GC01P206507 |
| SLC22A4 | GC05P132294 |
| FTL | GC19P048965 |
| UIMC1 | GC05M176905 |
| MSX2 | GC05P174724 |
| MFGE8 | GC15M088898 |
| ITGB7 | GC12M053191 |
| RFC1 | GC04M039291 |
| GNB3 | GC12P006839 |
| SLC39A1 | GC01M153960 |
| RORC | GC01M151806 |
| CFI | GC04M109732 |
| LINC00313 | GC21M043440 |
| HOXA11 | GC07M027487 |
| CD1E | GC01P158354 |
| REG1A | GC02P079120 |
| NUPR1 | GC16M028532 |
| CARD9 | GC09M136361 |
| FAR2P1 | GC02M130012 |
| KDM5C | GC0XM053176 |
| PRAL | GC17M006773 |
| BRD7 | GC16M050313 |
| GTF2I | GC07P074660 |
| PIP5K1C | GC19M004531 |
| FOXP2 | GC07P114086 |
| NTSR1 | GC20P062708 |
| CSNK1E | GC22M055249 |
| ZNF268 | GC12P133181 |
| CSF2RB | GC22P036913 |
| SLC22A5 | GC05P132369 |
| PEX1 | GC07M092487 |
| CYB5A | GC18M074250 |
| SURF1 | GC09M133351 |
| EEF2 | GC19M003976 |
| MLLT3 | GC09M020341 |
| CAPN1 | GC11P068856 |
| PDE4D | GC05M058969 |
| MAGED2 | GC0XP054807 |
| PUF60 | GC08M143816 |
| KPNA2 | GC17P068035 |
| PURA | GC05P140076 |
| AQP2 | GC12P049950 |
| CT45A1 | GC0XP135713 |
| MIR20B | GC0XM134217 |
| TRIM29 | GC11M120111 |
| IST1 | GC16P071920 |
| PSMA4 | GC15P078540 |
| RERE | GC01M008364 |
| STAT2 | GC12M056341 |
| TGM3 | GC20P002296 |
| PAEP | GC09P135561 |
| ST8SIA1 | GC12M022063 |
| BACH1 | GC21P029194 |
| VASP | GC19P052028 |
| DGCR8 | GC22P020080 |
| DPF2 | GC11P068981 |
| YWHAH | GC22P031944 |
| NNT-AS1 | GC05M043950 |
| UBE2N | GC12M093406 |
| RUVBL1 | GC03M128064 |
| WASF3 | GC13P026557 |
| MIR511 | GC10P017845 |
| GPX2 | GC14M064939 |
| LOC111589215 | GC17P051430 |
| STEAP3 | GC02P119222 |
| P2RY2 | GC11P073202 |
| TCHP | GC12P109900 |
| LINC02633 | GC10P043313 |
| UTF1 | GC10P133230 |
| ICAM3 | GC19M010388 |
| PDK1 | GC02P172555 |
| CDKN2D | GC19M010566 |
| MIR302A | GC04M112751 |
| RAB7A | GC03P133360 |
| DOK7 | GC04P003465 |
| IRAK4 | GC12P043758 |
| RORA | GC15M060488 |
| SOX3 | GC0XM140502 |
| EBF3 | GC10M129835 |
| LYZ | GC12P069348 |
| MRC1 | GC10P017809 |
| KCNMB2-AS1 | GC03M178525 |
| OLFM4 | GC13P053028 |
| ANKRD26 | GC10M026938 |
| CWF19L1 | GC10M100232 |
| EGOT | GC03M004790 |
| CCL7 | GC17P034270 |
| KSR1 | GC17P027456 |
| M6PR | GC12M009008 |
| PIWIL1 | GC12P130337 |
| PDLIM5 | GC04P094451 |
| EIF2S1 | GC14P067359 |
| SLC8A1 | GC02M040078 |
| TBX20 | GC07M035237 |
| ABCG1 | GC21P042199 |
| CISH | GC03M050974 |
| FLOT1 | GC06M061233 |
| MAOA | GC0XP043654 |
| GSK3A | GC19M061925 |
| PUM1 | GC01M030931 |
| CPS1 | GC02P210477 |
| BCHE | GC03M165772 |
| FCGR1A | GC01P149788 |
| CD248 | GC11M066314 |
| PSMC5 | GC17P063827 |
| MAP2K7 | GC19P007903 |
| PRPF6 | GC20P063981 |
| ADGRE5 | GC19P014381 |
| MIR367 | GC04M112647 |
| PMM2 | GC16P008788 |
| CLDN11 | GC03P170418 |
| MIEN1 | GC17M039728 |
| TET1 | GC10P068560 |
| PCAT4 | GC04P079827 |
| ACVR2B | GC03P038453 |
| ERC1 | GC12P000972 |
| STC2 | GC05M173314 |
| MLXIPL | GC07M073593 |
| MUC21 | GC06P076847 |
| SERBP1 | GC01M067407 |
| MALINC1 | GC05M140073 |
| CCR8 | GC03P039757 |
| FGF5 | GC04P080266 |
| GGH | GC08M063015 |
| CNTN1 | GC12P040692 |
| RACGAP1 | GC12M049978 |
| NFATC4 | GC14P024365 |
| LAMP3 | GC03M183122 |
| CLMP | GC11M123069 |
| ABCB5 | GC07P020615 |
| LCOR | GC10P096832 |
| LINC01234 | GC12M113584 |
| ITGA8 | GC10M015513 |
| CHD1 | GC05M098853 |
| ADORA2B | GC17P015927 |
| TRPC4 | GC13M037636 |
| CDK11B | GC01M005159 |
| GDF1 | GC19M018843 |
| CCR9 | GC03P046268 |
| WWC1 | GC05P168291 |
| NCR3LG1 | GC11P017351 |
| NDUFS4 | GC05P053560 |
| GATA5 | GC20M062464 |
| SCAT1 | GC17M078605 |
| ADORA2A | GC22P024417 |
| POLQ | GC03M121431 |
| CTSS | GC01M150730 |
| CEP55 | GC10P093496 |
| TFAP2B | GC06P077131 |
| TES | GC07P116210 |
| THRA | GC17P040058 |
| CLDN6 | GC16M003014 |
| ALKBH3 | GC11P043902 |
| GIPC1 | GC19M014450 |
| BRF1 | GC14M112089 |
| ZIC2 | GC13P099981 |
| GJC1 | GC17M044808 |
| RAPGEF1 | GC09M131576 |
| RRP1B | GC21P043659 |
| PBX2 | GC06M032184 |
| CA12 | GC15M063321 |
| SLC34A1 | GC05P177459 |
| TRPM7 | GC15M050552 |
| ZCCHC8 | GC12M122472 |
| MARCKS | GC06P113857 |
| SYNPO2 | GC04P118850 |
| RAD18 | GC03M008775 |
| TBXAS1 | GC07P139777 |
| SMARCC2 | GC12M056531 |
| CLSPN | GC01M035720 |
| ACAN | GC15P112336 |
| LIMA1 | GC12M050175 |
| GTF2H4 | GC06P076845 |
| CIC | GC19P042268 |
| CLDN23 | GC08P008701 |
| ZNF703 | GC08P037695 |
| NEFH | GC22P029480 |
| STEAP2 | GC07P090167 |
| TRIB3 | GC20P000361 |
| SLC1A5 | GC19M062097 |
| CELF2 | GC10P010462 |
| KLK2 | GC19P050861 |
| OVCA2 | GC17P002041 |
| CUL4B | GC0XM120524 |
| FLOT2 | GC17M033776 |
| AGAP2 | GC12M057723 |
| FKBP1A | GC20M001369 |
| PA2G4 | GC12P057064 |
| RECQL5 | GC17M075626 |
| HPS4 | GC22M026443 |
| C1S | GC12P018578 |
| ALOX5AP | GC13P030713 |
| ASIP | GC20P034413 |
| PORCN | GC0XP050296 |
| SELPLG | GC12M108621 |
| ABCC5 | GC03M183919 |
| XK | GC0XP037685 |
| LINC00210 | GC01P217892 |
| ARHGAP27P1 | GC17M065196 |
| EPHA1 | GC07M143390 |
| KIR2DS5 | GC19Mr00079 |
| HSD11B1 | GC01P209686 |
| RCN1 | GC11P032090 |
| FGFBP1 | GC04M015937 |
| RPS16 | GC19M039433 |
| EPHB3 | GC03P184561 |
| FUCA1 | GC01M023845 |
| HSD3B1 | GC01P119507 |
| GCH1 | GC14M054842 |
| EIF3I | GC01P032221 |
| ARID2 | GC12P045729 |
| SNAPC5 | GC15M079253 |
| G3BP1 | GC05P151771 |
| HGS | GC17P081683 |
| SMC4 | GC03P160399 |
| CRABP2 | GC01M156701 |
| ATAD3B | GC01P003381 |
| ALX4 | GC11M044238 |
| PPIG | GC02P169584 |
| ERRFI1 | GC01M008004 |
| KANK1 | GC09P000474 |
| TIMP4 | GC03M012153 |
| AIM2 | GC01M159062 |
| MIR489 | GC07M093483 |
| MBD2 | GC18M054151 |
| CRAT | GC09M129094 |
| EPHX2 | GC08P027490 |
| COL6A1 | GC21P045981 |
| WDR11 | GC10P120851 |
| DVL2 | GC17M007225 |
| GRIN1 | GC09P137138 |
| PLS3 | GC0XP115560 |
| TRIM27 | GC06M028903 |
| LDOC1 | GC0XM141111 |
| CTNNA3 | GC10M065912 |
| CXCL3 | GC04M074036 |
| MAPKAPK3 | GC03P050611 |
| GARS1 | GC07P030580 |
| PRPH | GC12P049293 |
| TNFRSF18 | GC01M001203 |
| SIRT6 | GC19M004174 |
| SNRNP200 | GC02M097769 |
| RPL29 | GC03M052024 |
| PON2 | GC07M095404 |
| LIN7C | GC11M027494 |
| POU2F2 | GC19M042086 |
| SOX1 | GC13P112067 |
| COL4A4 | GC02M226973 |
| ADAM8 | GC10M133262 |
| LINC01194 | GC05P012578 |
| ADCYAP1 | GC18P000895 |
| SERPINB13 | GC18P063586 |
| DPAGT1 | GC11M119096 |
| CELSR1 | GC22M046360 |
| MIR491 | GC09P020716 |
| VPS33A | GC12M122229 |
| FRS2 | GC12P069471 |
| SPAG9 | GC17M050962 |
| TRAP1 | GC16M006450 |
| ATXN3 | GC14M099702 |
| HIPK2 | GC07M139561 |
| UBB | GC17P016380 |
| PPP1R1B | GC17P039626 |
| LINC00342 | GC02M095807 |
| POLR2L | GC11M002669 |
| MAP3K11 | GC11M084684 |
| SYT7 | GC11M061513 |
| PDIA3 | GC15P043746 |
| RCC2 | GC01M017406 |
| PCAT14 | GC22P033473 |
| LINC01186 | GC0XM046258 |
| CAPN2 | GC01P223701 |
| HBP1 | GC07P107168 |
| HEXA | GC15M072340 |
| NCR2 | GC06P077045 |
| MX1 | GC21P041420 |
| EIF5A | GC17P007306 |
| SLC6A2 | GC16P055656 |
| DDX39B | GC06M031530 |
| NDFIP1 | GC05P142108 |
| LBP | GC20P038346 |
| LGR6 | GC01P202193 |
| MAPK11 | GC22M050263 |
| TAB2 | GC06P149218 |
| FBN2 | GC05M128257 |
| SOX18 | GC20M064047 |
| TNFSF9 | GC19P006531 |
| POFUT1 | GC20P032207 |
| EVA1A | GC02M075469 |
| PAPPA | GC09P118342 |
| ARNTL | GC11P013276 |
| HSD11B2 | GC16P067433 |
| PAK6 | GC15P040217 |
| AZGP1 | GC07M099967 |
| SEMA4F | GC02P074654 |
| AATBC | GC21M043806 |
| CABLES1 | GC18P023134 |
| PCAT18 | GC18M026922 |
| CFHR1 | GC01P196822 |
| E2F7 | GC12M077021 |
| LINC-PINT | GC07M130971 |
| NOD1 | GC07M030424 |
| HIF1A-AS2 | GC14M061747 |
| DDX17 | GC22M038483 |
| CGB7 | GC19M049054 |
| ALDH1A2 | GC15M079106 |
| ATP5F1A | GC18M046081 |
| UPK1A | GC19P051406 |
| MSH5 | GC06P076879 |
| NUP133 | GC01M229463 |
| CAMTA1 | GC01P006928 |
| DBH | GC09P133636 |
| BANF1 | GC11P066002 |
| CASP4 | GC11M104942 |
| PPL | GC16M006504 |
| PDZD2 | GC05P031639 |
| POLD3 | GC11P074526 |
| APOBEC3G | GC22P039085 |
| PTGIS | GC20M049503 |
| BCAS2 | GC01M114567 |
| NUP62 | GC19M049906 |
| LAPTM4B | GC08P097775 |
| MAPRE2 | GC18P034976 |
| LY75 | GC02M159803 |
| RLIM | GC0XM074669 |
| CYP4B1 | GC01P046757 |
| NEDD8 | GC14M024216 |
| PCSK2 | GC20P017226 |
| SLC39A6 | GC18M036108 |
| LTBP2 | GC14M074498 |
| MAML1 | GC05P179732 |
| SORT1 | GC01M109310 |
| LMOD1 | GC01M201896 |
| FOXCUT | GC06P001604 |
| TNFRSF14 | GC01P002555 |
| FXYD5 | GC19P035154 |
| LSP1 | GC11P001852 |
| POLR2E | GC19M001086 |
| TOPBP1 | GC03M133600 |
| UGT1A9 | GC02P233671 |
| DUOX2 | GC15M045092 |
| PBX3 | GC09P125747 |
| HNMT | GC02P137964 |
| BLOC1S5-TXNDC5 | GC06M007890 |
| CTBP2 | GC10M124984 |
| ARRDC3 | GC05M091368 |
| CSMD1 | GC08M002953 |
| TM9SF4 | GC20P032109 |
| HADHA | GC02M026190 |
| HEY1 | GC08M079764 |
| UBR5 | GC08M102252 |
| ZNF224 | GC19P044094 |
| THEMIS2 | GC01P027872 |
| PCSK1 | GC05M096391 |
| APRT | GC16M088810 |
| MEF2D | GC01M156463 |
| DLEU2 | GC13M049913 |
| LTB4R | GC14P024311 |
| PIK3C3 | GC18P041955 |
| PPP5C | GC19P052069 |
| WASF2 | GC01M027404 |
| OGT | GC0XP071534 |
| EYA2 | GC20P046894 |
| BRD3 | GC09M134030 |
| BCL2L12 | GC19P052342 |
| PER2 | GC02M238244 |
| TNXB | GC06M061322 |
| GNAT1 | GC03P050274 |
| EMG1 | GC12P006970 |
| FZD10 | GC12P130162 |
| SLCO1B3 | GC12P020810 |
| RNF168 | GC03M196468 |
| NEXN-AS1 | GC01M077955 |
| PINX1 | GC08M010764 |
| SOAT1 | GC01P179262 |
| MIR136 | GC14P108755 |
| VAPB | GC20P058389 |
| CDH16 | GC16M066908 |
| JARID2 | GC06P015385 |
| EEF1A2 | GC20M063488 |
| REG4 | GC01M119794 |
| GLO1 | GC06M061455 |
| SLC45A2 | GC05M033944 |
| IL1RAPL2 | GC0XP104566 |
| PIP5K1A | GC01P151198 |
| EGR2 | GC10M062811 |
| HEXB | GC05P074640 |
| NR5A2 | GC01P199996 |
| CCDC88A | GC02M055287 |
| FXR1 | GC03P180868 |
| KIF2A | GC05P062306 |
| ANXA4 | GC02P069644 |
| MIR138-2 | GC16P056981 |
| MPP1 | GC0XM154779 |
| ATP1A1 | GC01P116372 |
| SLC5A8 | GC12M101155 |
| IRF2 | GC04M184387 |
| MIR371A | GC19P053787 |
| UBAC2 | GC13P099200 |
| CXCL16 | GC17M004733 |
| FUT8 | GC14P065411 |
| YWHAB | GC20P044885 |
| KAT8 | GC16P039473 |
| PABPC1 | GC08M100685 |
| UGT8 | GC04P114598 |
| ENDOG | GC09P128818 |
| CLDN2 | GC0XP106900 |
| YWHAG | GC07M076608 |
| MIR376C | GC14P109075 |
| POGLUT1 | GC03P119468 |
| SLC31A1 | GC09P113221 |
| XAB2 | GC19M007619 |
| CNR2 | GC01M023870 |
| LRAT | GC04P154626 |
| HNRNPL | GC19M038836 |
| WT1-AS | GC11P032434 |
| LDHB | GC12M021635 |
| SOX6 | GC11M015949 |
| CERS1 | GC19M018868 |
| PKN1 | GC19P014433 |
| KHSRP | GC19M006413 |
| RNF8 | GC06P077022 |
| TSPAN32 | GC11P002302 |
| TRPC1 | GC03P142724 |
| H4C1 | GC06P076613 |
| SLC35C2 | GC20M046345 |
| CDK20 | GC09M087966 |
| PINK1 | GC01P020634 |
| CD200R1 | GC03M112921 |
| SMYD3 | GC01M245749 |
| BDKRB2 | GC14P096205 |
| MUCL1 | GC12P054830 |
| IRF6 | GC01M209785 |
| ARG2 | GC14P067619 |
| EIF3E | GC08M108163 |
| UBA52 | GC19P018563 |
| CCT3 | GC01M156308 |
| FGB | GC04P154587 |
| CPQ | GC08P096645 |
| AGGF1 | GC05P077029 |
| PRUNE2 | GC09M076611 |
| HOXA1 | GC07M027092 |
| HNRNPM | GC19P008444 |
| SCN10A | GC03M038713 |
| UBQLN2 | GC0XP056563 |
| RGS2 | GC01P192809 |
| HSD17B3 | GC09M096240 |
| SALL2 | GC14M021521 |
| PC | GC11M066848 |
| PAF1 | GC19M039385 |
| NDRG2 | GC14M021016 |
| KDM3B | GC05P138352 |
| PNPLA6 | GC19P007534 |
| ITGB8 | GC07P020329 |
| WNT9A | GC01M227920 |
| TUBA1C | GC12P049188 |
| KIF7 | GC15M089608 |
| HBB-LCR | GC11P005334 |
| SLC43A1 | GC11M084394 |
| KPNB1 | GC17P047649 |
| HUS1 | GC07M048023 |
| ATP2A3 | GC17M003923 |
| ZNRF3 | GC22P028883 |
| SELENOP | GC05M042800 |
| CPA1 | GC07P130380 |
| LGR4 | GC11M027365 |
| EIF6 | GC20M035278 |
| SLC25A1 | GC22M019264 |
| IL13RA1 | GC0XP118727 |
| C1QBP | GC17M005432 |
| IL27 | GC16M028645 |
| CD160 | GC01P145719 |
| FSTL1 | GC03M120392 |
| PRDX5 | GC11P064317 |
| SERPINB9 | GC06M002887 |
| HOTAIRM1 | GC07P027095 |
| PDCD7 | GC15M065117 |
| LGALS7B | GC19P051539 |
| PTGER3 | GC01M070852 |
| DTL | GC01P212035 |
| ADRB1 | GC10P114044 |
| FOXO4 | GC0XP071095 |
| KLF9 | GC09M070384 |
| NAA80 | GC03M050952 |
| OXT | GC20P003068 |
| HLA-DMB | GC06M032934 |
| NAGA | GC22M042058 |
| HEY2 | GC06P125730 |
| CTSE | GC01M206009 |
| SPRR3 | GC01P153001 |
| HYAL3 | GC03M050951 |
| KAT2A | GC17M042113 |
| GCLM | GC01M093885 |
| DOCK6 | GC19M011199 |
| KIF14 | GC01M200551 |
| FOSL2 | GC02P028392 |
| LINC02412 | GC12P093175 |
| EFL1 | GC15M082131 |
| IRS4 | GC0XM108720 |
| TFF2 | GC21M042346 |
| CCN5 | GC20P044715 |
| MTHFD1L | GC06P150865 |
| ATP1B2 | GC17P010613 |
| FCN3 | GC01M027729 |
| ZFP42 | GC04P187995 |
| YTHDF1 | GC20M063195 |
| NBEAL2 | GC03P046979 |
| MMP19 | GC12M055835 |
| MIR99B | GC19P051692 |
| PLK3 | GC01P044799 |
| BGN | GC0XP153494 |
| ADARB1 | GC21P045073 |
| RAB1A | GC02M065072 |
| COL4A6 | GC0XM108155 |
| MAT2A | GC02P085746 |
| KISS1R | GC19P002167 |
| FAM3D-AS1 | GC03P058607 |
| PPP1R10 | GC06M030600 |
| LRP8 | GC01M053243 |
| ENSG00000266919 | GC17M033810 |
| F7 | GC13P113105 |
| HSD3B2 | GC01P119414 |
| ZFP57 | GC06M029672 |
| ANXA11 | GC10M080150 |
| TSPY1 | GC0YP009469 |
| HTRA3 | GC04P008269 |
| KRT6A | GC12M052488 |
| BAMBI | GC10P028685 |
| BMPER | GC07P033944 |
| CLDND1 | GC03M098497 |
| FSCN2 | GC17P081541 |
| CRTC3 | GC15P090529 |
| CDH15 | GC16P089171 |
| CPLANE1 | GC05M037199 |
| PNN | GC14P039175 |
| IGSF8 | GC01M160061 |
| EIF4A1 | GC17P007572 |
| APPL1 | GC03P057227 |
| PDS5B | GC13P032586 |
| MIR572 | GC04P017217 |
| LORICRIN | GC01P153262 |
| MTA2 | GC11M084550 |
| SCD | GC10P100347 |
| LMX1B | GC09P126614 |
| EEF1E1-BLOC1S5 | GC06M008019 |
| SNRNP70 | GC19P049085 |
| FABP5 | GC08P081282 |
| PYY | GC17M043952 |
| LARP1 | GC05P154682 |
| REV1 | GC02M099383 |
| TLX2 | GC02P074534 |
| AJUBA | GC14M022971 |
| MMP21 | GC10M125756 |
| COLQ | GC03M019737 |
| RPRD1B | GC20P038033 |
| SIPA1 | GC11P065638 |
| CAST | GC05P096525 |
| LY96 | GC08P073991 |
| CDC42BPA | GC01M226989 |
| RFC4 | GC03M186789 |
| RIPK3 | GC14M024336 |
| TRPM2 | GC21P044350 |
| BATF2 | GC11M064987 |
| TMPRSS4 | GC11P118077 |
| RND3 | GC02M150468 |
| RALGDS | GC09M133097 |
| MCPH1 | GC08P006406 |
| MIR217 | GC02M055982 |
| PMPCA | GC09P136410 |
| PCSEAT | GC21P041577 |
| FGF19 | GC11M084873 |
| DND1 | GC05M140670 |
| HLA-DOB | GC06M061363 |
| SKIL | GC03P170357 |
| PCAT5 | GC10P035729 |
| SP100 | GC02P230415 |
| NSD3 | GC08M038269 |
| SLC39A7 | GC06P033200 |
| HMCES | GC03P129278 |
| NR2C2 | GC03P014947 |
| TNFRSF12A | GC16P003018 |
| F8 | GC0XM154835 |
| POU3F3 | GC02P104855 |
| ASPH | GC08M061500 |
| RFC3 | GC13P033818 |
| FLII | GC17M018244 |
| RETREG1 | GC05M016472 |
| NAIP | GC05M070968 |
| DBN1 | GC05M177456 |
| PTAFR | GC01M028147 |
| GNA12 | GC07M002728 |
| NDN | GC15M023686 |
| CENPE | GC04M103105 |
| TNFAIP8L2 | GC01P151156 |
| IER3 | GC06M030743 |
| PDS5A | GC04M039824 |
| RUSC1-AS1 | GC01M155317 |
| NKIRAS1 | GC03M024167 |
| ARHGEF7 | GC13P111114 |
| SMURF2 | GC17M064542 |
| P2RY12 | GC03M151336 |
| TXNIP | GC01M145992 |
| PCSK9 | GC01P055039 |
| NUP93 | GC16P056970 |
| RPS18 | GC06P076924 |
| SRP72 | GC04P056466 |
| PRPF4 | GC09P113275 |
| TAT | GC16M071565 |
| S100A12 | GC01M153373 |
| ANKRD11 | GC16M089267 |
| ING4 | GC12M006650 |
| EPSTI1 | GC13M042886 |
| SERPINF2 | GC17P001742 |
| RYR2 | GC01P237042 |
| NAA15 | GC04P139301 |
| COL8A1 | GC03P099638 |
| CASP5 | GC11M104995 |
| DDX39A | GC19M014408 |
| DPPA4 | GC03M109326 |
| ATP2C1 | GC03P130850 |
| LTBR | GC12P006375 |
| ZBTB7A | GC19M004578 |
| RPL3 | GC22M055086 |
| MLF1 | GC03P158571 |
| CELF1 | GC11M084332 |
| UHRF2 | GC09P006413 |
| MIR9-2 | GC05M088666 |
| SSTR4 | GC20P023035 |
| MYO1C | GC17M001464 |
| IL37 | GC02P120918 |
| BNC1 | GC15M083255 |
| SREBF2 | GC22P041833 |
| COL6A2 | GC21P046098 |
| PMVK | GC01M154924 |
| CTCFL | GC20M057495 |
| KIAA0586 | GC14P058427 |
| MT2A | GC16P056939 |
| DIAPH1 | GC05M141516 |
| UBR4 | GC01M019074 |
| ANKK1 | GC11P113387 |
| GDF5 | GC20M035433 |
| MAP2K6 | GC17P069414 |
| NUDT6 | GC04M122888 |
| USP22 | GC17M020999 |
| KLK11 | GC19M062307 |
| SPINK7 | GC05P148312 |
| XPO5 | GC06M043522 |
| HSPA1B | GC06P076882 |
| HUWE1 | GC0XM053532 |
| STK17A | GC07P043582 |
| KIF23 | GC15P069414 |
| EGLN2 | GC19P051782 |
| PTPRO | GC12P018866 |
| DUSP4 | GC08M029334 |
| MAP1LC3B | GC16P087384 |
| PLOD2 | GC03M146069 |
| RING1 | GC06P033208 |
| USP28 | GC11M113797 |
| ZNF148 | GC03M125225 |
| LINC01627 | GC09M037423 |
| SEC14L2 | GC22P030396 |
| SEC24C | GC10P073744 |
| PSMC6 | GC14P052707 |
| KLK8 | GC19M050996 |
| CDH4 | GC20P061252 |
| HOXB7 | GC17M048607 |
| DCBLD2 | GC03M098795 |
| HOXD13 | GC02P176092 |
| YY1AP1 | GC01M155659 |
| TARBP2 | GC12P053499 |
| KCNA5 | GC12P005043 |
| STYK1 | GC12M019796 |
| PSMD10 | GC0XM108084 |
| TK2 | GC16M066508 |
| DYNC2I2 | GC09M128636 |
| GPC5 | GC13P091398 |
| IL1R2 | GC02P101991 |
| GHSR | GC03M172443 |
| SYMPK | GC19M045815 |
| PADI4 | GC01P017308 |
| TCF7L1 | GC02P085133 |
| NR6A1 | GC09M124517 |
| SPIB | GC19P050418 |
| ZYX | GC07P143381 |
| HNRNPC | GC14M021930 |
| FOXG1 | GC14P028766 |
| SLC4A2 | GC07P151057 |
| FKBP4 | GC12P002795 |
| STOM | GC09M121338 |
| RPL7A | GC09P133348 |
| TUBB4A | GC19M006496 |
| ADD1 | GC04P002855 |
| GPD2 | GC02P156435 |
| CLEC2D | GC12P018672 |
| SMCHD1 | GC18P002649 |
| AKR1A1 | GC01P045550 |
| ELF1 | GC13M040933 |
| DLST | GC14P074881 |
| DACH1 | GC13M071437 |
| SIRT7 | GC17M081911 |
| NDC80 | GC18P002571 |
| PLXNC1 | GC12P094150 |
| UBASH3B | GC11P122655 |
| SETD5 | GC03P009402 |
| DRD1 | GC05M175440 |
| MYL9 | GC20P036541 |
| CTSH | GC15M079642 |
| KLF17 | GC01P044048 |
| BCLAF1 | GC06M136256 |
| BLZF1 | GC01P169367 |
| PRKCG | GC19P053879 |
| ARFGEF2 | GC20P048921 |
| HLA-DQB2 | GC06M032756 |
| ACLY | GC17M041866 |
| ABCD1 | GC0XP153724 |
| MTA3 | GC02P042494 |
| ZBTB20 | GC03M114315 |
| CHAF1A | GC19P004402 |
| KCNE1 | GC21M034446 |
| GNS | GC12M064713 |
| SMAD5 | GC05P136132 |
| VIL1 | GC02P218419 |
| PROC | GC02P127418 |
| DEF6 | GC06P076985 |
| NAA10 | GC0XM153929 |
| ZMIZ1 | GC10P079068 |
| IMPG1 | GC06M075921 |
| HSPA14 | GC10P014847 |
| MBNL1 | GC03P152243 |
| IRAK3 | GC12P066242 |
| PES1 | GC22M030576 |
| VAV2 | GC09M133761 |
| AIMP1 | GC04P106315 |
| CCKAR | GC04M026483 |
| DHRS9 | GC02P169064 |
| OXA1L | GC14P022766 |
| RALY | GC20P033993 |
| HNRNPUL1 | GC19P041262 |
| ATG16L1 | GC02P233246 |
| DVL3 | GC03P184155 |
| LINC02042 | GC03M112737 |
| UBAP2L | GC01P154219 |
| LINC01628 | GC02M066923 |
| MIR381 | GC14P109079 |
| ASAP1 | GC08M130052 |
| RDH12 | GC14P067701 |
| DOCK8-AS1 | GC09M000226 |
| DOCK1 | GC10P126905 |
| GSDMA | GC17P050345 |
| ERVW-1 | GC07M092468 |
| NRIP1 | GC21M014961 |
| ACKR2 | GC03P042804 |
| DLGAP5 | GC14M055148 |
| MIR301A | GC17M059151 |
| MIR103A1 | GC05M168560 |
| SND1 | GC07P127652 |
| CLDN8 | GC21M030214 |
| SPOCK1 | GC05M136975 |
| ANXA6 | GC05M151100 |
| RIPK2 | GC08P089786 |
| ZFYVE9 | GC01P052142 |
| PSMD2 | GC03P184298 |
| CBFA2T2 | GC20P033490 |
| CLDN10 | GC13P095433 |
| APOBEC3B | GC22P038982 |
| SMURF1 | GC07M099027 |
| CRB3 | GC19P006463 |
| DUSP19 | GC02P183078 |
| RPL23 | GC17M038847 |
| NFIC | GC19P003314 |
| PSMD6 | GC03M063973 |
| TTLL12 | GC22M055113 |
| TM4SF1 | GC03M149370 |
| LTA4H | GC12M096000 |
| KRT2 | GC12M052695 |
| CDH6 | GC05P031193 |
| HLA-F | GC06P076807 |
| SLK | GC10P103967 |
| GCM1 | GC06M061696 |
| PTPRA | GC20P002864 |
| MNT | GC17M002384 |
| CORO1C | GC12M108645 |
| FAM98A | GC02M033532 |
| EFNB1 | GC0XP068828 |
| PFKP | GC10P003066 |
| ILF2 | GC01M153661 |
| DEPDC1 | GC01M068474 |
| DMTN | GC08P022048 |
| PCBP2 | GC12P053452 |
| USP6 | GC17P005116 |
| GRK5 | GC10P119207 |
| ZKSCAN3 | GC06P028349 |
| STOML2 | GC09M035099 |
| SSB | GC02P169791 |
| SLC12A4 | GC16M068065 |
| HORMAD1 | GC01M151449 |
| S100A10 | GC01M151955 |
| H1-5 | GC06M061822 |
| CBX2 | GC17P079778 |
| MMP17 | GC12P131828 |
| CALM2 | GC02M047124 |
| XCL1 | GC01P168576 |
| JMJD6 | GC17M076718 |
| SPINK5 | GC05P148025 |
| NFIA | GC01P060865 |
| SLC25A11 | GC17M004937 |
| ESRP1 | GC08P094808 |
| ANAPC2 | GC09M137174 |
| NDUFAF2 | GC05P060945 |
| PSMC3 | GC11M084330 |
| TRPC3 | GC04M121879 |
| CYP2B6 | GC19P040991 |
| SNHG3 | GC01P028506 |
| CNDP2 | GC18P074495 |
| MAPK13 | GC06P077004 |
| ST3GAL1 | GC08M133454 |
| EIF4A3 | GC17M080135 |
| NR0B2 | GC01M027041 |
| EIF3B | GC07P002354 |
| HCFC2 | GC12P104064 |
| CCHCR1 | GC06M061251 |
| PTPRD | GC09M008307 |
| IL36G | GC02P112973 |
| MAPKAPK5 | GC12P111842 |
| PAGE4 | GC0XP049829 |
| ST3GAL4 | GC11P126355 |
| SIK3 | GC11M116843 |
| PSME3 | GC17P042824 |
| LINC01502 | GC09P135574 |
| PYGO2 | GC01M154957 |
| CASZ1 | GC01M010642 |
| DDX6 | GC11M118748 |
| PAX9 | GC14P036657 |
| PSMA3 | GC14P058244 |
| MMP26 | GC11P004706 |
| APLN | GC0XM129645 |
| PIK3R5 | GC17M008878 |
| CCT7 | GC02P073233 |
| ADIPOR1 | GC01M202940 |
| ZNF384 | GC12M006841 |
| RPA3 | GC07M007974 |
| CD8B | GC02M086815 |
| CHRNE | GC17M004897 |
| MT-ND2 | GCMTP004472 |
| RPL19 | GC17P039200 |
| SEPTIN2 | GC02P241316 |
| ABCF1 | GC06P030571 |
| DUSP3 | GC17M043766 |
| NLK | GC17P049653 |
| PTPRF | GC01P043527 |
| TDRG1 | GC06P077040 |
| USP25 | GC21P015730 |
| TUT1 | GC11M084544 |
| SEM1 | GC07M096483 |
| CD93 | GC20M023079 |
| PTPRZ1 | GC07P121873 |
| KRT12 | GC17M040861 |
| KRT3 | GC12M052789 |
| RMI1 | GC09P083980 |
| BMP5 | GC06M055753 |
| PKP4 | GC02P158456 |
| MAP3K3 | GC17P063622 |
| NOLC1 | GC10P102152 |
| RARRES1 | GC03M158696 |
| RPS3A | GC04P151099 |
| RPL22 | GC01M006179 |
| PYHIN1 | GC01P158900 |
| USP53 | GC04P119212 |
| NSUN2 | GC05M006599 |
| LINC00958 | GC11M012877 |
| EPHB1 | GC03P134795 |
| TIGAR | GC12P018506 |
| HSPA2 | GC14P064535 |
| PTGDR | GC14P052267 |
| SDCBP2 | GC20M001309 |
| ADK | GC10P074152 |
| ZFPM2-AS1 | GC08M105546 |
| LTB | GC06M061305 |
| CARD8 | GC19M062134 |
| RNF31 | GC14P024146 |
| PPP1R15A | GC19P048872 |
| ETV5 | GC03M186046 |
| TNFAIP6 | GC02P151357 |
| NEB | GC02M151485 |
| CDK5RAP3 | GC17P047967 |
| E2F6 | GC02M011434 |
| SRSF3 | GC06P077008 |
| UGT2B17 | GC04M068537 |
| AGO1 | GC01P035869 |
| BNIP3L | GC08P026296 |
| REG3A | GC02M079157 |
| TRIM14 | GC09M098830 |
| ZNF180 | GC19M061998 |
| KLK5 | GC19M050943 |
| HCRT | GC17M042185 |
| DNAJA3 | GC16P004425 |
| TRIM32 | GC09P116687 |
| SRSF5 | GC14P069727 |
| ATF7 | GC12M053527 |
| FNDC3B | GC03P172039 |
| PTPN14 | GC01M214348 |
| PANX1 | GC11P094128 |
| LY6E | GC08P143017 |
| ZMAT3 | GC03M179017 |
| PGRMC1 | GC0XP119236 |
| CBR1 | GC21P036069 |
| HTR3A | GC11P113974 |
| MMP15 | GC16P058025 |
| CBX3 | GC07P026201 |
| EFNA3 | GC01P155078 |
| TNFAIP8 | GC05P119268 |
| MUCL3 | GC06P078375 |
| CCT8 | GC21M029055 |
| MYH3 | GC17M010628 |
| ANK3 | GC10M060026 |
| DCUN1D3 | GC16M020869 |
| SI | GC03M164978 |
| HRH4 | GC18P024460 |
| PSMD4 | GC01P151256 |
| ZFX | GC0XP024148 |
| CREB3L4 | GC01P153967 |
| ANOS1 | GC0XM008528 |
| GNAI3 | GC01P109548 |
| XAF1 | GC17P006757 |
| LINC00339 | GC01P022263 |
| IL19 | GC01P206770 |
| TAFAZZIN | GC0XP154413 |
| RUBCNL | GC13M046343 |
| SEMA3E | GC07M083363 |
| H1-3 | GC06M061821 |
| CLEC3B | GC03P046252 |
| MAPKAP1 | GC09M125437 |
| FAAH | GC01P046394 |
| ATP2B1 | GC12M089588 |
| SLC1A1 | GC09P004490 |
| WASL | GC07M123681 |
| RNF2 | GC01P185045 |
| CCNG2 | GC04P077158 |
| AP2M1 | GC03P184174 |
| ACTL6A | GC03P179562 |
| S1PR2 | GC19M010223 |
| DNAH17 | GC17M078423 |
| SLC25A13 | GC07M096120 |
| ANKRD1 | GC10M090912 |
| LINC00963 | GC09P129483 |
| SMARCD1 | GC12P050085 |
| DPYSL2 | GC08P026514 |
| ULK1 | GC12P131894 |
| STK17B | GC02M196133 |
| CAP1 | GC01P040101 |
| MIR744 | GC17P012081 |
| ARHGDIA | GC17M081867 |
| MYH8 | GC17M010390 |
| TFDP2 | GC03M141944 |
| MAPK6 | GC15P051952 |
| LSM2 | GC06M061319 |
| SLC29A3 | GC10P071320 |
| LOC111674463 | GC07P117476 |
| PCGEM1 | GC02P192749 |
| HLA-DOA | GC06M033004 |
| STEAP4 | GC07M088372 |
| SPRR1B | GC01P153031 |
| MAGED1 | GC0XP051803 |
| KRT6B | GC12M052446 |
| PITX1 | GC05M135027 |
| FOLR2 | GC11P072216 |
| LEMD3 | GC12P065169 |
| DLG5 | GC10M077790 |
| BLMH | GC17M030248 |
| NEK6 | GC09P124259 |
| VASH1 | GC14P076761 |
| DUXAP9 | GC14P030457 |
| SOX13 | GC01P204074 |
| ASAH2 | GC10M050182 |
| PDE2A | GC11M072576 |
| RPL12 | GC09M127447 |
| PREX1 | GC20M048624 |
| DIDO1 | GC20M062877 |
| EPX | GC17P058192 |
| CDK14 | GC07P090471 |
| GOLPH3 | GC05M032124 |
| PCSK7 | GC11M117199 |
| GATAD1 | GC07P092447 |
| ARID3B | GC15P074541 |
| RDH11 | GC14M067676 |
| GABBR1 | GC06M029555 |
| PMF1 | GC01P156212 |
| BOK | GC02P241558 |
| EEF2K | GC16P022217 |
| CARM1 | GC19P010871 |
| UBE2V2 | GC08P047998 |
| PSME2 | GC14M024143 |
| ADIPOR2 | GC12P001670 |
| MIR608 | GC10P100974 |
| TPD52L2 | GC20P063865 |
| NR2F1 | GC05P093583 |
| CEP78 | GC09P078236 |
| KCNN3 | GC01M154697 |
| MLKL | GC16M074672 |
| INCENP | GC11P062142 |
| ZNF341 | GC20P033731 |
| DAPK3 | GC19M003958 |
| SNHG6 | GC08M066921 |
| CLIC4 | GC01P024745 |
| SOX7 | GC08M010723 |
| NINJ1 | GC09M093121 |
| LGMN | GC14M092703 |
| ZNF750 | GC17M082829 |
| MASP2 | GC01M011026 |
| FBLIM1 | GC01P015756 |
| CTNNBIP1 | GC01M009848 |
| DFFA | GC01M010456 |
| FER1L4 | GC20M035558 |
| PSMD9 | GC12P125523 |
| DUSP22 | GC06P000292 |
| PLXNA1 | GC03P126988 |
| RGS1 | GC01P192575 |
| EDN2 | GC01M041478 |
| PRKACG | GC09M069024 |
| ABCC10 | GC06P043427 |
| LOC106721785 | GC13P032314 |
| STK3 | GC08M098372 |
| KAT6A | GC08M041929 |
| CPSF6 | GC12P069239 |
| RABGAP1L | GC01P174159 |
| YTHDC2 | GC05P113513 |
| MIR422A | GC15M063870 |
| TRIM26 | GC06M030184 |
| UGT1A8 | GC02P233618 |
| LGALS8 | GC01P236518 |
| ABT1 | GC06P076642 |
| DLL3 | GC19P039498 |
| HCG11 | GC06P076630 |
| UBQLN4 | GC01M156033 |
| PANTR1 | GC02M104806 |
| GLDC | GC09M006522 |
| FADS1 | GC11M061799 |
| GCNT2 | GC06P010492 |
| SSPN | GC12P026118 |
| SYNM | GC15P099098 |
| KMT2E | GC07P104978 |
| EPB42 | GC15M043608 |
| CEBPD | GC08M047759 |
| TFAM | GC10P058385 |
| IL17RC | GC03P009917 |
| MIR1-1 | GC20P063248 |
| LINC00941 | GC12P030757 |
| ZIC1 | GC03P147393 |
| RPS11 | GC19P049496 |
| ALDH1L1 | GC03M126103 |
| ARPC2 | GC02P218217 |
| MAP4K3 | GC02M039249 |
| LINC01554 | GC05P095838 |
| NPR1 | GC01P153840 |
| TOB1 | GC17M050862 |
| RBMS1 | GC02M160272 |
| PIK3C2B | GC01M204422 |
| MNX1-AS1 | GC07P157010 |
| PLXNB1 | GC03M048403 |
| ATP1A3 | GC19M041966 |
| CARMIL1 | GC06P025350 |
| NOB1 | GC16M069944 |
| ALYREF | GC17M081887 |
| CLEC4A | GC12P018599 |
| PTGDS | GC09P137026 |
| THRAP3 | GC01P036224 |
| PUM2 | GC02M020316 |
| MED14 | GC0XM040648 |
| MAP4 | GC03M047850 |
| ITPR2 | GC12M026336 |
| HIF1AN | GC10P100529 |
| PON3 | GC07M095359 |
| TCF20 | GC22M042160 |
| TNRC6C | GC17P077959 |
| NAIF1 | GC09M128061 |
| LMO4 | GC01P087329 |
| CIRBP | GC19P001259 |
| RPS13 | GC11M017507 |
| PAK5 | GC20M009538 |
| TALDO1 | GC11P001551 |
| ARHGAP15 | GC02P143070 |
| ADAMTS1 | GC21M026835 |
| DAAM1 | GC14P059188 |
| FUBP1 | GC01M077944 |
| HIF3A | GC19P046297 |
| ATIC | GC02P215311 |
| PYM1 | GC12M055902 |
| F12 | GC05M177402 |
| DOK1 | GC02P074549 |
| NSF | GC17P046590 |
| BRD9 | GC05M001068 |
| KIF1A | GC02M240713 |
| ZNF652 | GC17M049289 |
| FOXF2 | GC06P001390 |
| CBY1 | GC22P038656 |
| GIT1 | GC17M029573 |
| SMARCC1 | GC03M047585 |
| GOLM1 | GC09M086026 |
| EFNB3 | GC17P010617 |
| TNKS2 | GC10P091798 |
| HESX1 | GC03M057207 |
| DAZAP1 | GC19P001407 |
| BRINP3 | GC01M190067 |
| PRSS2 | GC07P147617 |
| OVOL2 | GC20M017956 |
| WNT9B | GC17P046833 |
| REPS2 | GC0XP016946 |
| PIR | GC0XM015402 |
| ELF4 | GC0XM130064 |
| TTF1 | GC09M132375 |
| FZD2 | GC17P044557 |
| PAG1 | GC08M080967 |
| CCL27 | GC09M034662 |
| DRD4 | GC11P001544 |
| H3C12 | GC06M061831 |
| MYO18A | GC17M033780 |
| AHSG | GC03P186622 |
| KLK13 | GC19M062311 |
| GSTO2 | GC10P104268 |
| APLNR | GC11M057233 |
| SVEP1 | GC09M110365 |
| SIGMAR1 | GC09M034634 |
| DKK2 | GC04M106921 |
| SGPL1 | GC10P070815 |
| EMP1 | GC12P013196 |
| H1-4 | GC06P077457 |
| CCN6 | GC06P112053 |
| RPL7 | GC08M073290 |
| KDM3A | GC02P086440 |
| EYA4 | GC06P133240 |
| PCBP1 | GC02P070087 |
| TTF2 | GC01P117060 |
| U2AF1L4 | GC19M061812 |
| NIBAN1 | GC01M184791 |
| SESN2 | GC01P028270 |
| ACTN2 | GC01P236686 |
| TEAD4 | GC12P002959 |
| PRPS1 | GC0XP107628 |
| SLC22A3 | GC06P160348 |
| ILF3-DT | GC19M010652 |
| KCNK2 | GC01P215005 |
| PPP2R5C | GC14P109164 |
| RPS25 | GC11M119015 |
| USP1 | GC01P062436 |
| EAF2 | GC03P121835 |
| GLIS3 | GC09M003816 |
| LMO7 | GC13P075620 |
| CPA4 | GC07P130293 |
| ELF2 | GC04M139028 |
| LGI1 | GC10P093757 |
| CACNA2D1 | GC07M081946 |
| PHEX | GC0XP022032 |
| CDKL5 | GC0XP018425 |
| UBE4B | GC01P010032 |
| UVSSA | GC04P001341 |
| NT5C2 | GC10M103088 |
| MIR302B | GC04M112760 |
| ATXN10 | GC22P045673 |
| H6PD | GC01P009234 |
| GADD45B | GC19P002476 |
| RPS6KA6 | GC0XM084058 |
| ARHGAP35 | GC19P046860 |
| PTS | GC11P112226 |
| NCAPG | GC04P017812 |
| SLC5A1 | GC22P032043 |
| ERLIN2 | GC08P037736 |
| CHD3 | GC17P010622 |
| TRIO | GC05P014143 |
| TRIB2 | GC02P012717 |
| CLCN2 | GC03M184346 |
| MED15 | GC22P033315 |
| TLE3 | GC15M070047 |
| LOC107303338 | GC03P012022 |
| PDYN | GC20M001978 |
| TBC1D3 | GC17M038181 |
| SDC3 | GC01M030869 |
| CHRM2 | GC07P136868 |
| ASCL2 | GC11M002744 |
| IRX3 | GC16M054283 |
| COPS8 | GC02P237085 |
| RBM14 | GC11P069114 |
| RBM15 | GC01P110338 |
| PSMD1 | GC02P231056 |
| EHF | GC11P034621 |
| POU4F1 | GC13M078598 |
| RHOG | GC11M003848 |
| GLRX | GC05M095752 |
| SIAH1 | GC16M048357 |
| RPL38 | GC17P074204 |
| ARL4C | GC02M234493 |
| EIF5A2 | GC03M170888 |
| MAP3K9 | GC14M070722 |
| LARGE1 | GC22M035371 |
| PSMD3 | GC17P039980 |
| TNRC6B | GC22P040044 |
| HSD17B10 | GC0XM053431 |
| CLCN3 | GC04P169612 |
| POU3F2 | GC06P098834 |
| DIO2 | GC14M080197 |
| LINC01503 | GC09P129332 |
| WDR5 | GC09P134135 |
| MAGEA6 | GC0XM152766 |
| IMMT | GC02M086144 |
| ADGRE2 | GC19M014733 |
| MYDGF | GC19M004641 |
| APOA4 | GC11M116820 |
| SP7 | GC12M053326 |
| MFHAS1 | GC08M008783 |
| COPS2 | GC15M049106 |
| DDIT4 | GC10P072273 |
| MYEOV | GC11P069313 |
| IL27RA | GC19P014031 |
| UBE2D1 | GC10P058334 |
| CHD5 | GC01M006104 |
| EFS | GC14M023356 |
| KIF2C | GC01P044739 |
| SYTL2 | GC11M085694 |
| PEX13 | GC02P061017 |
| RAB23 | GC06M061749 |
| MT3 | GC16P056589 |
| BATF | GC14P075523 |
| NHLRC2 | GC10P113854 |
| LETM1 | GC04M001811 |
| TP53I3 | GC02M024078 |
| CALM3 | GC19P046601 |
| USF1 | GC01M161039 |
| CGAS | GC06M073414 |
| DUOX1 | GC15P045129 |
| KIFC1 | GC06P033391 |
| ETFA | GC15M079552 |
| A2M | GC12M009067 |
| ALDH3A1 | GC17M019737 |
| HOXB5 | GC17M048591 |
| BDKRB1 | GC14P096290 |
| GPR65 | GC14P088005 |
| FRMD4A | GC10M013643 |
| KIF22 | GC16P039367 |
| ARPC3 | GC12M110434 |
| TMSB10 | GC02P084905 |
| ALDH9A1 | GC01M165677 |
| FOSB | GC19P045467 |
| CCT4 | GC02M061868 |
| TUBA4A | GC02M219249 |
| VPS45 | GC01P150088 |
| INHBB | GC02P121070 |
| DHPS | GC19M013460 |
| CHERP | GC19M016517 |
| CARD10 | GC22M055077 |
| MPG | GC16P009804 |
| ANKRD55 | GC05M056099 |
| ATAD5 | GC17P049800 |
| SEC16A | GC09M136440 |
| TMEM97 | GC17P028319 |
| SLC39A4 | GC08M144409 |
| RNU4ATAC | GC02P121601 |
| CYFIP1 | GC15M022867 |
| SP140 | GC02P230203 |
| VTI1A | GC10P112446 |
| IFIT3 | GC10P089327 |
| TUBB4B | GC09P137241 |
| PGD | GC01P010398 |
| PDLIM7 | GC05M177483 |
| PSMC2 | GC07P103344 |
| FRAT1 | GC10P097319 |
| SCGB1D2 | GC11P062260 |
| SIRT5 | GC06P013574 |
| ULBP2 | GC06P149941 |
| RAD23A | GC19P013573 |
| H1-1 | GC06M026018 |
| SORD | GC15P045023 |
| NFIX | GC19P013569 |
| ATF6B | GC06M032115 |
| KLK15 | GC19M050825 |
| GSTO1 | GC10P104235 |
| LEMD2 | GC06M061422 |
| TAB1 | GC22P039419 |
| IL36A | GC02P113005 |
| PITX3 | GC10M102230 |
| PFN2 | GC03M149964 |
| RHOU | GC01P228644 |
| NELFCD | GC20P058981 |
| WNK3 | GC0XM054279 |
| CAPZB | GC01M019339 |
| CDKN2A-DT | GC09P021967 |
| FEZF1 | GC07M122301 |
| EIF4B | GC12P053006 |
| PDE3B | GC11P014643 |
| EVI5 | GC01M092508 |
| FAM111B | GC11P059107 |
| SHROOM3 | GC04P076435 |
| KDM2B | GC12M121402 |
| CALML4 | GC15M068190 |
| ADAM28 | GC08P024294 |
| FZD5 | GC02M207762 |
| DHX16 | GC06M030653 |
| RGS5 | GC01M163111 |
| CNBP | GC03M129167 |
| LGALS2 | GC22M037570 |
| PABPC4 | GC01M039560 |
| GTF2H1 | GC11P018454 |
| ALDH7A1 | GC05M126541 |
| PDK2 | GC17P051774 |
| NELFA | GC04M002119 |
| VAMP8 | GC02P085561 |
| CNMD | GC13M052704 |
| DDX11 | GC12P031073 |
| PPFIA1 | GC11P070270 |
| GAK | GC04M000849 |
| SLC18A2 | GC10P117241 |
| RPL13A | GC19P049487 |
| HOXA3 | GC07M027481 |
| IL18RAP | GC02P102418 |
| GALNT3 | GC02M165747 |
| CD37 | GC19P052305 |
| HOXB4 | GC17M048575 |
| RIMS1 | GC06P071886 |
| SRI | GC07M088205 |
| DDX53 | GC0XP022999 |
| WDFY3 | GC04M084669 |
| PPARGC1B | GC05P149730 |
| PHF8 | GC0XM053936 |
| CLCF1 | GC11M067364 |
| SPAM1 | GC07P123925 |
| CRNKL1 | GC20M020034 |
| CXCL17 | GC19M042428 |
| ARHGAP31 | GC03P119294 |
| C15orf48 | GC15P045430 |
| UGT2B15 | GC04M068646 |
| TRIM25 | GC17M056836 |
| TCF19 | GC06P076850 |
| CFB | GC06P031945 |
| RFC5 | GC12P118013 |
| BCCIP | GC10P125823 |
| SLC35F2 | GC11M107790 |
| KIF20A | GC05P138189 |
| ZNF667-AS1 | GC19P056477 |
| RTN4R | GC22M020241 |
| SALL1 | GC16M051135 |
| ETV7 | GC06M061445 |
| C2CD3 | GC11M074012 |
| TEP1 | GC14M020365 |
| GRB10 | GC07M050590 |
| MSMP | GC09M035753 |
| DPT | GC01M168696 |
| ZYG11A | GC01P052842 |
| SUN2 | GC22M055082 |
| NID2 | GC14M052004 |
| RGS14 | GC05P177357 |
| FUT6 | GC19M005830 |
| NR1D1 | GC17M040092 |
| HLA-H | GC06P076814 |
| CCL24 | GC07M076562 |
| H2AC20 | GC01P150096 |
| MIR302C | GC04M112752 |
| IMPDH2 | GC03M050872 |
| RPL24 | GC03M101681 |
| VPS52 | GC06M061394 |
| GPBAR1 | GC02P218259 |
| ROMO1 | GC20P035699 |
| PPP2CB | GC08M030762 |
| HK3 | GC05M176882 |
| PLEKHG5 | GC01M006466 |
| HAS1 | GC19M062343 |
| SLC12A6 | GC15M034229 |
| MYOF | GC10M093306 |
| DDAH2 | GC06M031727 |
| NIBAN2 | GC09M127558 |
| LACAT1 | GC17P077254 |
| MFAP2 | GC01M016974 |
| HIP1 | GC07M075533 |
| SOX21-AS1 | GC13P094712 |
| H4C8 | GC06M061840 |
| RUVBL2 | GC19P048993 |
| MT-CO3 | GCMTP009209 |
| GLRX3 | GC10P130136 |
| RGS3 | GC09P118316 |
| RPL8 | GC08M145292 |
| BTG3 | GC21M017594 |
| KRT34 | GC17M041377 |
| POU1F1 | GC03M087259 |
| LRPPRC | GC02M043850 |
| PYCR1 | GC17M081932 |
| PKP3 | GC11P000436 |
| GADD45G | GC09P089605 |
| RAB38 | GC11M087809 |
| HDAC10 | GC22M050245 |
| PARVB | GC22P043999 |
| LETMD1 | GC12P051047 |
| U2AF2 | GC19P055654 |
| DRD3 | GC03M114128 |
| LNPEP | GC05P096935 |
| RPL13 | GC16P091043 |
| SLC25A5 | GC0XP119468 |
| RAD1 | GC05M034905 |
| MELTF | GC03M196980 |
| METAP2 | GC12P095473 |
| LAMTOR5 | GC01M110401 |
| SNRPA | GC19P040750 |
| ZNF699 | GC19M009294 |
| VPS4B | GC18M063389 |
| ESPL1 | GC12P053268 |
| DLG4 | GC17M007189 |
| HEATR3 | GC16P050065 |
| HASPIN | GC17P004168 |
| PRKAR2A | GC03M048744 |
| PYGM | GC11M064746 |
| WDR26 | GC01M224385 |
| DAP3 | GC01P155758 |
| FOXK1 | GC07P004682 |
| DNMBP | GC10M099875 |
| POMGNT2 | GC03M043121 |
| IFITM3 | GC11M000319 |
| SPDYE4 | GC17M010143 |
| DPM1 | GC20M050934 |
| PSMD14 | GC02P161308 |
| BTLA | GC03M112463 |
| NEU3 | GC11P074988 |
| PTRH2 | GC17M059674 |
| LONP1 | GC19M005691 |
| RAB31 | GC18P009701 |
| BNC2 | GC09M016410 |
| CAPG | GC02M085394 |
| SLC16A4 | GC01M110362 |
| FOXD3 | GC01P063323 |
| CRTC2 | GC01M153947 |
| MTNR1B | GC11P092969 |
| CA8 | GC08M060187 |
| RPS2 | GC16M006274 |
| TWNK | GC10P100993 |
| ZMYM2 | GC13P020023 |
| PCSK5 | GC09P075890 |
| RCOR1 | GC14P102592 |
| CCNL2 | GC01M001385 |
| USP4 | GC03M049277 |
| MAP7 | GC06M136342 |
| BLOC1S6 | GC15P045588 |
| ULBP1 | GC06P149963 |
| CHD1L | GC01P147686 |
| MAU2 | GC19P019320 |
| ERVFRD-1 | GC06M011103 |
| CETN2 | GC0XM152827 |
| LPIN1 | GC02P011677 |
| MIR7-1 | GC09M091385 |
| ARID3A | GC19P002168 |
| FCGRT | GC19P049506 |
| CHRNA9 | GC04P040337 |
| MEST | GC07P130486 |
| RANBP1 | GC22P020115 |
| RIOK1 | GC06P007389 |
| SHARPIN | GC08M144098 |
| AARS1 | GC16M070550 |
| LYAR | GC04M004270 |
| RPL9 | GC04M039452 |
| CAPN10 | GC02P240586 |
| FGFRL1 | GC04P001197 |
| HOXC13 | GC12P053938 |
| FZD9 | GC07P073433 |
| VOPP1 | GC07M055434 |
| KRBOX4 | GC0XP046481 |
| BAIAP2L1 | GC07M098294 |
| RPS9 | GC19P063068 |
| KCMF1 | GC02P084971 |
| AAAS | GC12M053307 |
| CDSN | GC06M031115 |
| WDR6 | GC03P049007 |
| CACUL1 | GC10M118674 |
| SPRTN | GC01P231337 |
| SNIP1 | GC01M037534 |
| ADGRB1 | GC08P142449 |
| GHRHR | GC07P030938 |
| GNRH2 | GC20P003866 |
| CRHR1 | GC17P045784 |
| GCC2 | GC02P108432 |
| ATP5F1B | GC12M056639 |
| PRAC1 | GC17M048721 |
| TRIM68 | GC11M004577 |
| EFNA4 | GC01P155063 |
| UACA | GC15M070654 |
| BAZ1B | GC07M073440 |
| ABCA12 | GC02M214931 |
| TASOR2 | GC10P005684 |
| PRDM5 | GC04M120686 |
| CRLF1 | GC19M018572 |
| C3AR1 | GC12M008058 |
| TRA2B | GC03M185914 |
| TKTL1 | GC0XP154295 |
| TRIM8 | GC10P102643 |
| CISD2 | GC04P102868 |
| H3C10 | GC06P076683 |
| LNX1 | GC04M053504 |
| PAH | GC12M102836 |
| MIR155HG | GC21P025581 |
| RAP1GAP | GC01M021596 |
| UCP1 | GC04M140559 |
| TAGLN2 | GC01M159918 |
| HAS3 | GC16P069105 |
| PPP1R9B | GC17M050133 |
| KDM4A | GC01P043650 |
| NOP53 | GC19P052154 |
| TRADD | GC16M067154 |
| SH2B1 | GC16P039275 |
| BPI | GC20P038304 |
| BLOC1S3 | GC19P045178 |
| SCG5 | GC15P032641 |
| HTR1A | GC05M063960 |
| BTN3A2 | GC06P026365 |
| ATRAID | GC02P027212 |
| MARCKSL1 | GC01M032334 |
| HEPH | GC0XP066162 |
| POLR2B | GC04P056977 |
| CSN1S1 | GC04P069932 |
| PIWIL2 | GC08P022275 |
| DNAJA1 | GC09P033025 |
| PSMC3IP | GC17M042572 |
| SCYL1 | GC11P065525 |
| GRK6 | GC05P177403 |
| CAMK2A | GC05M150219 |
| EGFL6 | GC0XP013569 |
| IRF9 | GC14P024161 |
| FBLN2 | GC03P013565 |
| POLD2 | GC07M044114 |
| CASC20 | GC20P006358 |
| SIX2 | GC02M045005 |
| PTMA | GC02P231707 |
| GRM5 | GC11M088504 |
| NASP | GC01P045583 |
| KCNJ6 | GC21M037607 |
| EMILIN2 | GC18P002846 |
| RPL4 | GC15M066498 |
| RCHY1 | GC04M075479 |
| MOK | GC14M102224 |
| LFNG | GC07P002512 |
| ATAD3A | GC01P003384 |
| STRAP | GC12P015882 |
| MIR563 | GC03P015873 |
| ACER3 | GC11P076860 |
| SLC1A4 | GC02P064988 |
| PPT2 | GC06P032153 |
| RNF14 | GC05P141958 |
| TNRC6A | GC16P024611 |
| EDA | GC0XP069618 |
| USP10 | GC16P084702 |
| MIR181D | GC19P013874 |
| NLRC5 | GC16P056990 |
| NMU | GC04M055595 |
| TOMM20 | GC01M235109 |
| FPR2 | GC19P051752 |
| NUP205 | GC07P135557 |
| PEX2 | GC08M076980 |
| MT-ND3 | GCMTP010061 |
| MIR190A | GC15P113081 |
| CFAP251 | GC12P125609 |
| SERPIND1 | GC22P033337 |
| EIF2S2 | GC20M034088 |
| KPNA1 | GC03M122421 |
| RAP2B | GC03P153162 |
| OPRD1 | GC01P028812 |
| DCAF8 | GC01M160215 |
| ZNF77 | GC19M002933 |
| CHP2 | GC16P024139 |
| PSPN | GC19M006375 |
| RPN2 | GC20P037178 |
| DTX1 | GC12P113056 |
| BFSP1 | GC20M017493 |
| PGM1 | GC01P063593 |
| PI16 | GC06P077018 |
| RPLP1 | GC15P092255 |
| USP14 | GC18P000158 |
| H3-4 | GC01M228427 |
| CCL8 | GC17P034319 |
| SLC30A1 | GC01M211571 |
| CLEC4D | GC12P008509 |
| ADH5 | GC04M099070 |
| RPS8 | GC01P044775 |
| ADAMTS4 | GC01M161184 |
| OR51E2 | GC11M004680 |
| NMT1 | GC17P050900 |
| STXBP5 | GC06P147204 |
| CORIN | GC04M047596 |
| AFAP1L2 | GC10M114281 |
| MMS19 | GC10M097458 |
| POLR1G | GC19P052023 |
| FOXC2-AS1 | GC16M086566 |
| ARL2BP | GC16P057245 |
| MYH14 | GC19P052389 |
| H3C4 | GC06M061832 |
| PTOV1 | GC19P052357 |
| NKX6-1 | GC04M084491 |
| ITLN1 | GC01M160876 |
| DNAJB6 | GC07P157335 |
| RBM3 | GC0XP048574 |
| LINC00115 | GC01M005101 |
| ANKHD1 | GC05P145497 |
| SFRP4 | GC07M037912 |
| COPB1 | GC11M014436 |
| HNRNPD | GC04M082352 |
| MIR346 | GC10M086264 |
| FRY | GC13P031877 |
| FOXQ1 | GC06P001312 |
| NDUFS1 | GC02M206114 |
| DUXAP8 | GC22P015784 |
| FBXO4 | GC05P042000 |
| SNHG17 | GC20M038621 |
| TSR1 | GC17M002322 |
| MIR92B | GC01P155195 |
| GSDMD | GC08P143553 |
| LY6G5B | GC06P076871 |
| EHD2 | GC19P047713 |
| CCT2 | GC12P069585 |
| AGFG1 | GC02P227473 |
| GFER | GC16P001984 |
| SIX4 | GC14M060709 |
| LPAR6 | GC13M048389 |
| PCDH7 | GC04P030722 |
| CTSA | GC20P045890 |
| NCBP1 | GC09P097633 |
| IL20 | GC01P206866 |
| ARHGEF12 | GC11P120336 |
| HNRNPDL | GC04M082422 |
| PRDX4 | GC0XP023665 |
| HMGN2 | GC01P026473 |
| KLF14 | GC07M130731 |
| EEF1D | GC08M143579 |
| STT3A | GC11P125592 |
| TRPM1 | GC15M031001 |
| UBE2D3 | GC04M102794 |
| EXOC2 | GC06M000485 |
| OLIG1 | GC21P033070 |
| NFIL3 | GC09M091409 |
| SHOX2 | GC03M158095 |
| RLN2 | GC09M005306 |
| MANF | GC03P051385 |
| MIR124-2 | GC08P064379 |
| CNTLN | GC09P017124 |
| COL12A1 | GC06M075084 |
| NBPF3 | GC01P021765 |
| MIR100HG | GC11M122029 |
| KMT5A | GC12P125547 |
| MLPH | GC02P237485 |
| TSHZ3 | GC19M031855 |
| RTRAF | GC14P051992 |
| DSC1 | GC18M031129 |
| NEK7 | GC01P198156 |
| DSPP | GC04P087608 |
| SENP3 | GC17P010607 |
| PDHX | GC11P034894 |
| POLA1 | GC0XP024693 |
| LZTS2 | GC10P100996 |
| SOAT2 | GC12P053103 |
| EIF3G | GC19M010115 |
| CPM | GC12M068842 |
| LUM | GC12M091102 |
| ADAMTS2 | GC05M179110 |
| DOCK5 | GC08P025184 |
| PRIMPOL | GC04P184649 |
| HTR1B | GC06M077478 |
| DENR | GC12P122752 |
| TP53INP1 | GC08M094925 |
| IRX5 | GC16P054930 |
| MIPEP | GC13M023730 |
| USP15 | GC12P062260 |
| PTGER1 | GC19M014444 |
| SNHG14 | GC15P037740 |
| DHX30 | GC03P047802 |
| POLR1A | GC02M086021 |
| MAGEA12 | GC0XP152733 |
| FAM215A | GC17P043917 |
| FGG | GC04M154604 |
| MS4A2 | GC11P060088 |
| PTPN23 | GC03P047416 |
| PLTP | GC20M045898 |
| VPS33B | GC15M090998 |
| TIRAP | GC11P126284 |
| ARPC5 | GC01M183621 |
| PEX16 | GC11M084305 |
| HNRNPR | GC01M023303 |
| PCDH10 | GC04P133149 |
| MAOB | GC0XM043766 |
| UGT2B7 | GC04P069051 |
| MTMR3 | GC22P033694 |
| MT1G | GC16M056666 |
| PHF20 | GC20P035771 |
| TRIP10 | GC19P006737 |
| SEC61A1 | GC03P128051 |
| KIR3DX1 | GC19P062536 |
| CXCL6 | GC04P073837 |
| SIM2 | GC21P036699 |
| IRX1 | GC05P003596 |
| MAP3K15 | GC0XM019360 |
| H2AC21 | GC01M151386 |
| CGN | GC01P151483 |
| KRTCAP2 | GC01M155187 |
| CPT1A | GC11M068754 |
| RPS5 | GC19P058386 |
| INPP4A | GC02P098465 |
| RPL23A | GC17P028719 |
| SFRP5 | GC10M097766 |
| RPS23 | GC05M082273 |
| CMKLR1 | GC12M108288 |
| NUMBL | GC19M040665 |
| PRPF19 | GC11M060890 |
| EPHA8 | GC01P022563 |
| RIPK4 | GC21M041739 |
| TMEM8B | GC09P035814 |
| SERPINB6 | GC06M002948 |
| GSDMC | GC08M129705 |
| MIB1 | GC18P021704 |
| LINC00668 | GC18M006922 |
| DSG4 | GC18P031377 |
| ZNF408 | GC11P046700 |
| CYP2C8 | GC10M095038 |
| SLC9A3R2 | GC16P009893 |
| KIF3B | GC20P032277 |
| CAPN9 | GC01P230747 |
| SIRPG | GC20M001628 |
| RGR | GC10P090599 |
| ADPRH | GC03P119579 |
| COBL | GC07M051016 |
| RPL10 | GC0XP154389 |
| GABRA2 | GC04M046243 |
| TET3 | GC02P073986 |
| KLF12 | GC13M073686 |
| ATP6AP1 | GC0XP154428 |
| ASF1A | GC06P118894 |
| CKAP5 | GC11M084316 |
| ST3GAL3 | GC01P043705 |
| RGS4 | GC01P163038 |
| TRH | GC03P129974 |
| CYP26B1 | GC02M072129 |
| NEIL1 | GC15P075346 |
| MIP | GC12M056449 |
| TCFL5 | GC20M062841 |
| DLX5 | GC07M097020 |
| ZFR | GC05M032390 |
| MAST4 | GC05P066596 |
| SRPK2 | GC07M105110 |
| SLC2A5 | GC01M009036 |
| UGT1A | GC02P233592 |
| CAVIN2 | GC02M191835 |
| GPX7 | GC01P052602 |
| TCHH | GC01M152106 |
| DDX20 | GC01P111755 |
| CLEC6A | GC12P008455 |
| RSL1D1 | GC16M011833 |
| ADAMTS9 | GC03M064501 |
| MIR654 | GC14P109096 |
| IK | GC05P145500 |
| ALKBH1 | GC14M077672 |
| SPANXD | GC0XM141697 |
| C20orf204 | GC20P064916 |
| POU2F3 | GC11P120236 |
| SCGB2A1 | GC11P062227 |
| ZHX2 | GC08P122781 |
| SLC7A8 | GC14M023125 |
| CHST15 | GC10M124006 |
| LOXL1 | GC15P073925 |
| TASP1 | GC20M013105 |
| SMYD2 | GC01P214281 |
| CRLF3 | GC17M030769 |
| NDUFS8 | GC11P068030 |
| ADCY8 | GC08M130780 |
| POLL | GC10M101578 |
| ABHD11-AS1 | GC07P074558 |
| GEMIN4 | GC17M000744 |
| KIF4A | GC0XP070290 |
| MDH1 | GC02P063557 |
| KHDRBS3 | GC08P135457 |
| ALKBH5 | GC17P018183 |
| AATK | GC17M081117 |
| POLG2 | GC17M064477 |
| HM13 | GC20P031514 |
| ACYP2 | GC02P053970 |
| DEGS1 | GC01P224175 |
| TPH1 | GC11M018040 |
| KRT9 | GC17M041565 |
| RNASE1 | GC14M020801 |
| COMMD1 | GC02P061888 |
| PSD3 | GC08M018527 |
| MYBBP1A | GC17M004538 |
| MYO1D | GC17M032492 |
| BTN3A1 | GC06P026402 |
| ACADVL | GC17P007219 |
| DCUN1D5 | GC11M103051 |
| PLOD3 | GC07M101205 |
| EIF3L | GC22P037848 |
| MMP24 | GC20P035226 |
| NPEPPS | GC17P047522 |
| PPP2R5A | GC01P212285 |
| CKAP4 | GC12M106237 |
| H1-2 | GC06M026056 |
| SF3A1 | GC22M030331 |
| SH3BP4 | GC02P234951 |
| SUMF1 | GC03M003700 |
| TCTN1 | GC12P110614 |
| PDE4DIP | GC01P148808 |
| PCDH1 | GC05M141900 |
| PRR12 | GC19P049591 |
| MYO10 | GC05M016661 |
| RASGRF1 | GC15M078959 |
| RPS4X | GC0XM072255 |
| MRPL28 | GC16M000357 |
| CCNC | GC06M099542 |
| FAM120A | GC09P093716 |
| TNFRSF21 | GC06M047231 |
| WRNIP1 | GC06P002766 |
| SRR | GC17P002303 |
| CDIPT | GC16M035767 |
| ATP5PO | GC21M033904 |
| CFLAR-AS1 | GC02M201140 |
| ABCE1 | GC04P145097 |
| VGLL4 | GC03M011723 |
| SIAH2 | GC03M150741 |
| ERBIN | GC05P065931 |
| LRFN2 | GC06M061469 |
| WNK4 | GC17P050794 |
| PDHA1 | GC0XP019343 |
| KLF7 | GC02M207074 |
| KTN1 | GC14P055559 |
| MAGEA2 | GC0XM152749 |
| BICD2 | GC09M092711 |
| PTGES3 | GC12M056667 |
| COL16A1 | GC01M031653 |
| PDE3A | GC12P020294 |
| H4C15 | GC01M151374 |
| UBQLN1 | GC09M083659 |
| DMAP1 | GC01P044214 |
| UBE2V1 | GC20M050082 |
| CAMKK1 | GC17M003860 |
| WASHC4 | GC12P105108 |
| SOCS6 | GC18P070288 |
| MZF1 | GC19M062660 |
| CREM | GC10P035126 |
| DARS2 | GC01P173824 |
| RNH1 | GC11M002648 |
| NIPAL4 | GC05P157460 |
| ATP8B2 | GC01P154325 |
| CDO1 | GC05M115804 |
| RAB2A | GC08P060516 |
| MAGEA9 | GC0XP149781 |
| HECTD4 | GC12M112160 |
| RCAN1 | GC21M034513 |
| NAP1L1 | GC12M076036 |
| ITPKC | GC19P051776 |
| NFX1 | GC09P033292 |
| CAPZA1 | GC01P112619 |
| MCU | GC10P072692 |
| MIA | GC19P040771 |
| PSMC1 | GC14P090256 |
| CST7 | GC20P024949 |
| GPR143 | GC0XM009725 |
| NCK2 | GC02P105744 |
| ZNF169 | GC09P094259 |
| GEMIN2 | GC14P039114 |
| FOXN3 | GC14M099693 |
| DEFA5 | GC08M007057 |
| GALNT2 | GC01P230057 |
| SP4 | GC07P021434 |
| GNL2 | GC01M037566 |
| TSPAN4 | GC11P001564 |
| PHF19 | GC09M120855 |
| HYLS1 | GC11P125883 |
| DCAF7 | GC17P063550 |
| CASC18 | GC12P105704 |
| PLXDC2 | GC10P019769 |
| PLAA | GC09M026903 |
| CKS2 | GC09P089311 |
| NEIL2 | GC08P011769 |
| ISG20 | GC15P088635 |
| ARHGEF4 | GC02P130836 |
| GBX2 | GC02M236165 |
| NME3 | GC16M001770 |
| FBXO22 | GC15P075903 |
| HNRNPA3 | GC02P177212 |
| PKIB | GC06P122472 |
| GRHL3 | GC01P024425 |
| ABHD2 | GC15P089087 |
| ZNF639 | GC03P179323 |
| MRPL23 | GC11P001948 |
| BABAM2 | GC02P027889 |
| SINHCAF | GC12M031283 |
| BASP1 | GC05P017065 |
| GSE1 | GC16P085171 |
| TRIP12 | GC02M229763 |
| NR1I3 | GC01M161229 |
| SLC35E3 | GC12P068746 |
| GALK1 | GC17M075751 |
| LIMCH1 | GC04P041362 |
| ARF4 | GC03M057572 |
| MYL12B | GC18P003261 |
| TRMT10C | GC03P101561 |
| DNA2 | GC10M068414 |
| TUFM | GC16M035630 |
| PCGF1 | GC02M074505 |
| PURB | GC07M044879 |
| DYNLL1 | GC12P120469 |
| ENOX2 | GC0XM130623 |
| RHBDF1 | GC16M000058 |
| RPL14 | GC03P040458 |
| PTGFR | GC01P078303 |
| TNXA | GC06M061325 |
| TXNDC5 | GC06M007893 |
| PCSK6 | GC15M114818 |
| ARFGAP1 | GC20P063272 |
| DCD | GC12M054644 |
| PSMB10 | GC16M067937 |
| TBCK | GC04M106041 |
| NOL11 | GC17P067717 |
| WASHC5 | GC08M131396 |
| LINC00707 | GC10P006780 |
| PWP1 | GC12P107685 |
| RASGRP3 | GC02P033436 |
| PLA2G5 | GC01P020028 |
| EFHD2 | GC01P015409 |
| PUS1 | GC12P131929 |
| DOCK4 | GC07M111726 |
| PITRM1 | GC10M003138 |
| SRPX2 | GC0XP100648 |
| SLC25A24 | GC01M108134 |
| TGM4 | GC03P044874 |
| PCOTH | GC13P023889 |
| HMGB2 | GC04M173331 |
| ANGPTL2 | GC09M127087 |
| BLOC1S1 | GC12P055852 |
| HOXA7 | GC07M027153 |
| PIDD1 | GC11M002666 |
| H4C12 | GC06M061836 |
| ACVR1C | GC02M157526 |
| MEPE | GC04P087821 |
| AP3B2 | GC15M087751 |
| PLCD1 | GC03M038008 |
| SNX10 | GC07P026291 |
| TRIM39 | GC06P076830 |
| PDP1 | GC08P093857 |
| UBA2 | GC19P034428 |
| HELZ | GC17M067070 |
| OAT | GC10M124397 |
| KDSR | GC18M063327 |
| SLIT3 | GC05M168661 |
| LUC7L3 | GC17P050719 |
| DGKQ | GC04M000958 |
| HSD17B13 | GC04M087303 |
| RPS12 | GC06P132814 |
| CCL25 | GC19P008052 |
| PUS7 | GC07M105439 |
| NAV3 | GC12P077341 |
| H4C11 | GC06P076684 |
| DEFB103B | GC08M007430 |
| SLC10A2 | GC13M103043 |
| CCT5 | GC05P010236 |
| MYO1G | GC07M044962 |
| EBF2 | GC08M025841 |
| ARSH | GC0XP003006 |
| PSORS1C1 | GC06P031114 |
| LHX3 | GC09M136196 |
| ABCD3 | GC01P094418 |
| IFI30 | GC19P018173 |
| TSPAN8 | GC12M071125 |
| PI4K2A | GC10P097640 |
| POLM | GC07M044362 |
| RAB10 | GC02P026033 |
| PGA3 | GC11P061203 |
| PARP4 | GC13M024420 |
| FABP1 | GC02M088122 |
| BHLHE41 | GC12M026120 |
| ALDH1A3 | GC15P100877 |
| UBE3C | GC07P157138 |
| SORBS2 | GC04M185585 |
| SNRPB | GC20M002461 |
| KNTC1 | GC12P122527 |
| RPLP0 | GC12M120196 |
| DDX19A-DT | GC16M070553 |
| POLR3A | GC10M078393 |
| BTN2A2 | GC06P026382 |
| FAF2 | GC05P176447 |
| MNX1 | GC07M156994 |
| MTF1 | GC01M037810 |
| GRK3 | GC22P033592 |
| CDH8 | GC16M061647 |
| GLT8D1 | GC03M052694 |
| SGCB | GC04M052019 |
| MAN2A1 | GC05P109689 |
| MASTL | GC10P027154 |
| NME4 | GC16P000396 |
| HOXC8 | GC12P054899 |
| MIR302D | GC04M112648 |
| PPIB | GC15M064155 |
| PLXNA2 | GC01M208023 |
| USP48 | GC01M021678 |
| HCP5 | GC06P031400 |
| RRBP1 | GC20M017613 |
| FAIM2 | GC12M049866 |
| FADS2 | GC11P061792 |
| GUCY2F | GC0XM109372 |
| NUDT21 | GC16M056429 |
| LARP4 | GC12P050392 |
| PNPT1 | GC02M055634 |
| MYCT1 | GC06P152697 |
| BRAP | GC12M111642 |
| THOC1 | GC18M000204 |
| OGN | GC09M092383 |
| PIK3AP1 | GC10M096593 |
| PLEK2 | GC14M067386 |
| MIIP | GC01P012019 |
| CTNNBL1 | GC20P037693 |
| CNGA1 | GC04M047935 |
| GTPBP4 | GC10P000988 |
| WLS | GC01M068098 |
| MIR299 | GC14P109064 |
| FKBP10 | GC17P041812 |
| TLE2 | GC19M002997 |
| SFMBT1 | GC03M052930 |
| SLC25A3 | GC12P098593 |
| EMP3 | GC19P048321 |
| PRPF40A | GC02M152651 |
| EDIL3 | GC05M083940 |
| MIR452 | GC0XM151959 |
| GDF10 | GC10P047300 |
| COLGALT1 | GC19P017555 |
| NREP | GC05M111662 |
| WASF1 | GC06M110099 |
| HPX | GC11M006435 |
| IL17B | GC05M149371 |
| CEBPA-DT | GC19P051304 |
| MLLT11 | GC01P151118 |
| MIR885 | GC03M010413 |
| COL9A1 | GC06M070215 |
| EEF1G | GC11M084542 |
| H2BC14 | GC06P076682 |
| PPIL2 | GC22P034309 |
| DCDC2 | GC06M024171 |
| LINC01600 | GC06M002621 |
| GNG7 | GC19M002511 |
| MIR433 | GC14P108750 |
| RIF1 | GC02P151409 |
| LMX1A | GC01M165171 |
| SMG6 | GC17M002059 |
| MUC3B | GC07U903146 |
| FDFT1 | GC08P011795 |
| HOXC6 | GC12P053990 |
| NLRP12 | GC19M053793 |
| EME1 | GC17P050373 |
| UNC93B1 | GC11M067991 |
| PSORS1C2 | GC06M031137 |
| LINC00052 | GC15P087576 |
| ATP6V1E1 | GC22M017592 |
| FGGY | GC01P059296 |
| TMBIM6 | GC12P049707 |
| PTGR1 | GC09M111549 |
| GBP1 | GC01M089052 |
| EHHADH | GC03M185190 |
| BRK1 | GC03P010115 |
| PPA2 | GC04M105369 |
| BCL2L14 | GC12P012049 |
| ING5 | GC02P241702 |
| MSRA | GC08P010054 |
| UBR1 | GC15M042942 |
| G3BP2 | GC04M075642 |
| MIR19B1 | GC13P091529 |
| ATF5 | GC19P052362 |
| WDR48 | GC03P039052 |
| SYCP2 | GC20M059863 |
| PRSS3 | GC09P033750 |
| KRT71 | GC12M052543 |
| IQGAP2 | GC05P076403 |
| DEPTOR | GC08P119873 |
| GPN1 | GC02P027628 |
| AK1 | GC09M127866 |
| MXRA8 | GC01M001352 |
| MIR454 | GC17M059137 |
| RAB14 | GC09M121178 |
| SULT1A3 | GC16P030199 |
| ASTN2 | GC09M116425 |
| APOBEC3A | GC22P038952 |
| PLCL1 | GC02P197804 |
| OTX1 | GC02P063050 |
| MFN1 | GC03P179347 |
| GPS1 | GC17P082050 |
| STK38 | GC06M036493 |
| FKBPL | GC06M061328 |
| FGD4 | GC12P032407 |
| MIR519D | GC19P053713 |
| DUT | GC15P048331 |
| TRAPPC10 | GC21P044012 |
| DERL1 | GC08M123013 |
| PRSS16 | GC06P027247 |
| PRRX1 | GC01P170662 |
| CTDSPL | GC03P037861 |
| C1QTNF4 | GC11M084335 |
| ALOXE3 | GC17M010115 |
| TSPAN1 | GC01P046175 |
| MIR432 | GC14P108754 |
| KCTD12 | GC13M076880 |
| HNRNPF | GC10M043385 |
| ECI2 | GC06M004115 |
| VAMP7 | GC0XP155881 |
| MMRN1 | GC04P089879 |
| MYL3 | GC03M046836 |
| SNRPD2 | GC19M062042 |
| EMX2 | GC10P117542 |
| RGS12 | GC04P003292 |
| SOX8 | GC16P000981 |
| RPL18A | GC19P050939 |
| CTH | GC01P070411 |
| PDIA6 | GC02M010784 |
| OIP5 | GC15M041309 |
| THEM4 | GC01M151870 |
| SLC29A2 | GC11M084743 |
| SLC23A2 | GC20M004852 |
| RPS27L | GC15M079173 |
| HEG1 | GC03M124965 |
| PAK1IP1 | GC06P010712 |
| NHS | GC0XP017393 |
| H4C14 | GC01P149832 |
| RASSF8 | GC12P025959 |
| FIS1 | GC07M101239 |
| PTBP2 | GC01P096721 |
| NKX2-2 | GC20M021511 |
| PREP | GC06M105277 |
| YBX3 | GC12M019797 |
| OSR1 | GC02M019351 |
| LRIG3 | GC12M058872 |
| ALOX12B | GC17M010117 |
| BRD3OS | GC09P134096 |
| ANXA8 | GC10M047460 |
| CPSF4 | GC07P099438 |
| CLEC1B | GC12M019784 |
| RBM17 | GC10P006089 |
| GOLM2 | GC15P044289 |
| SIK2 | GC11P111692 |
| HAT1 | GC02P171922 |
| ERP29 | GC12P112013 |
| NDUFA12 | GC12M094898 |
| APOM | GC06P076872 |
| ZNF607 | GC19M037696 |
| MIR30C2 | GC06M071382 |
| PHIP | GC06M078934 |
| GABPA | GC21P025734 |
| SCGB1D1 | GC11P062208 |
| DIO3 | GC14P108873 |
| ALG3 | GC03M184244 |
| RPL36AL | GC14M049619 |
| PRECSIT | GC13M110864 |
| DARS1 | GC02M135905 |
| DXO | GC06M031969 |
| PRKCSH | GC19P011435 |
| DLX6-AS1 | GC07M096966 |
| FAM83H | GC08M143723 |
| LYPD3 | GC19M043460 |
| CCT6A | GC07P056051 |
| ATP13A3 | GC03M194402 |
| MIR103A2 | GC20P003917 |
| DMP1 | GC04P087650 |
| HIVEP3 | GC01M041506 |
| AS3MT | GC10P102869 |
| ATL1 | GC14P050532 |
| RPN1 | GC03M128619 |
| H4C9 | GC06P077480 |
| ASAP2 | GC02P009206 |
| EP400 | GC12P131949 |
| GZMK | GC05P055024 |
| HSPA1L | GC06M031809 |
| AMPD3 | GC11P010309 |
| PRAG1 | GC08M008318 |
| RO60 | GC01P193059 |
| PKD2L1 | GC10M100288 |
| OPA3 | GC19M045527 |
| HMGB3 | GC0XP150980 |
| DLX4 | GC17P049968 |
| RGS17 | GC06M153004 |
| PGA5 | GC11P061241 |
| SHPRH | GC06M145863 |
| ACER2 | GC09P019408 |
| YARS1 | GC01M032776 |
| MTBP | GC08P120426 |
| PCBD1 | GC10M070882 |
| BTBD9 | GC06M038168 |
| SLX1A | GC16P039399 |
| UBE2S | GC19M055399 |
| MIR3936HG | GC05M132312 |
| SMC5 | GC09P070258 |
| CAND1 | GC12P067270 |
| NFYC | GC01P040691 |
| AIF1L | GC09P131169 |
| NDUFA10 | GC02M239893 |
| TNFAIP2 | GC14P108809 |
| KLF13 | GC15P031326 |
| RPL21 | GC13P027251 |
| MTHFD2 | GC02P074186 |
| TLK1 | GC02M170990 |
| CBR3 | GC21P036134 |
| ANAPC10 | GC04M144831 |
| GCN1 | GC12M120128 |
| VPS28 | GC08M145249 |
| WWP1 | GC08P086342 |
| TMC1 | GC09P072521 |
| HTR2B | GC02M231108 |
| ZNF322 | GC06M060953 |
| UMOD | GC16M020344 |
| RBMX | GC0XM136848 |
| ZNF608 | GC05M124636 |
| OIP5-AS1 | GC15P041300 |
| F2RL2 | GC05M076615 |
| RPL34 | GC04P108620 |
| CPSF7 | GC11M061402 |
| MCFD2 | GC02M046901 |
| BCAT1 | GC12M024732 |
| LBX1 | GC10M101226 |
| DACT2 | GC06M168292 |
| EIF4G3 | GC01M020806 |
| MIB2 | GC01P003391 |
| UCN | GC02M027308 |
| LRIG2 | GC01P113073 |
| CTNNAL1 | GC09M108942 |
| HINT1 | GC05M131159 |
| ELOVL5 | GC06M053267 |
| SETD1B | GC12P125520 |
| MED24 | GC17M040019 |
| SEMA7A | GC15M074409 |
| TECTA | GC11P121101 |
| MYL6 | GC12P056158 |
| DDX21 | GC10P068956 |
| TTC28 | GC22M027978 |
| USP24 | GC01M055066 |
| KDM8 | GC16P027650 |
| ABO | GC09M133250 |
| LOC101059986 | GC05P139482 |
| TFDP3 | GC0XM133216 |
| MAGEA11 | GC0XP149688 |
| CARD16 | GC11M105041 |
| GINS2 | GC16M085676 |
| MAFK | GC07P001897 |
| BNIPL | GC01P151036 |
| MTCL1 | GC18P008705 |
| VARS1 | GC06M061318 |
| STX8 | GC17M009250 |
| PGC | GC06M041736 |
| BMP2K | GC04P078776 |
| DDX52 | GC17M037609 |
| SMC2 | GC09P104094 |
| DCST1-AS1 | GC01M155144 |
| ARHGAP21 | GC10M024583 |
| RPL17 | GC18M049488 |
| PLAAT3 | GC11M084589 |
| SOST | GC17M043753 |
| DUSP29 | GC10M075028 |
| CHAF1B | GC21P036385 |
| UHRF1BP1 | GC06P076981 |
| ADRM1 | GC20P062302 |
| ADRA2A | GC10P111077 |
| EXOC4 | GC07P133253 |
| ZNF618 | GC09P113876 |
| BATF3 | GC01M212686 |
| SACS | GC13M023288 |
| PGPEP1 | GC19P050951 |
| RIN3 | GC14P092513 |
| HOXB8 | GC17M048611 |
| RPL36 | GC19P005674 |
| FREM1 | GC09M014734 |
| ERLIN1 | GC10M100150 |
| MINCR | GC08M143281 |
| OXNAD1 | GC03P016784 |
| CYRIB | GC08M131072 |
| PPM1B | GC02P044167 |
| NPAS3 | GC14P032934 |
| FKBP8 | GC19M018503 |
| SEC13 | GC03M010293 |
| MXD1 | GC02P069897 |
| SCGN | GC06P025652 |
| ACO2 | GC22P041485 |
| FAAP24 | GC19P051292 |
| C19orf48 | GC19M050797 |
| PHF20L1 | GC08P132775 |
| CNTN6 | GC03P000978 |
| GALE | GC01M023795 |
| SLC22A16 | GC06M110424 |
| ADAMTS8 | GC11M130404 |
| ZSCAN9 | GC06P028224 |
| SGCD | GC05P155686 |
| HDLBP | GC02M241227 |
| TBRG4 | GC07M045100 |
| NFYA | GC06P077043 |
| C4BPA | GC01P207105 |
| DKKL1 | GC19P049360 |
| CITED1 | GC0XM072301 |
| LINC00319 | GC21P043446 |
| ASPRV1 | GC02M069932 |
| FILIP1 | GC06M075291 |
| MRGPRX2 | GC11M019077 |
| RRAGC | GC01M038856 |
| VTRNA2-1 | GC05M136081 |
| GSTM2 | GC01P109668 |
| H3C3 | GC06P076615 |
| CEP126 | GC11P101916 |
| H3C6 | GC06P077476 |
| RRAGA | GC09P019049 |
| TRDMT1 | GC10M017138 |
| CPB2 | GC13M046053 |
| CCNY | GC10P035254 |
| YBX2 | GC17M007288 |
| INTS11 | GC01M005140 |
| ARHGEF11 | GC01M156904 |
| PCBP4 | GC03M051957 |
| SF3A3 | GC01M037956 |
| DDIT4L | GC04M100185 |
| TAPBPL | GC12P006451 |
| STRN | GC02M036815 |
| LSM14A | GC19P034172 |
| MMUT | GC06M049430 |
| POLR3H | GC22M041525 |
| PHETA1 | GC12M111361 |
| KCNJ3 | GC02P154698 |
| SYCP1 | GC01P114854 |
| B3GALT4 | GC06P033277 |
| TRIM63 | GC01M026062 |
| RNF5 | GC06P076892 |
| ZBTB48 | GC01P006579 |
| CEP170 | GC01M243124 |
| MPI | GC15P074890 |
| GIPR | GC19P045668 |
| MAGEB2 | GC0XP030215 |
| SKA1 | GC18P050374 |
| HEXIM1 | GC17P050912 |
| CASP14 | GC19P015049 |
| RITA1 | GC12P113185 |
| HLA-DRB6 | GC06M061348 |
| CRISP3 | GC06M049727 |
| PADI2 | GC01M017066 |
| DSTN | GC20P017550 |
| TFCP2 | GC12M051093 |
| KRT75 | GC12M052425 |
| SEC23A | GC14M039031 |
| FBXO31 | GC16M087326 |
| GABRA1 | GC05P161847 |
| GABRG2 | GC05P162000 |
| CDK16 | GC0XP047217 |
| SIPA1L3 | GC19P037906 |
| LAPTM5 | GC01M030732 |
| SH3GL2 | GC09P017569 |
| FBH1 | GC10P005889 |
| SMOX | GC20P004120 |
| ADAM29 | GC04P174831 |
| AKNA | GC09M114334 |
| HP1BP3 | GC01M020742 |
| ARL6IP5 | GC03P069084 |
| ZNF142 | GC02M218637 |
| ZNF281 | GC01M200404 |
| CBX8 | GC17M079794 |
| CHN1 | GC02M174799 |
| OPRK1 | GC08M053227 |
| KPNA4 | GC03M160494 |
| TCN1 | GC11M084450 |
| RASSF3 | GC12P064507 |
| CLEC2A | GC12M019781 |
| ARL2 | GC11P065015 |
| RBM25 | GC14P073058 |
| SPECC1 | GC17P049532 |
| MIR492 | GC12P094834 |
| ANP32A | GC15M068778 |
| DEFA1 | GC08M006977 |
| CREB5 | GC07P028305 |
| DCTD | GC04M182890 |
| HES5 | GC01M002528 |
| RAB4A | GC01P229271 |
| BABAM1 | GC19P050921 |
| PITPNM3 | GC17M006451 |
| FOXD1 | GC05M073444 |
| LRRC8C | GC01P089615 |
| ZNF230 | GC19P044002 |
| EIF3F | GC11P007966 |
| SRGAP2 | GC01P206203 |
| RBMS3 | GC03P028575 |
| KCNJ15 | GC21P038157 |
| MIR363 | GC0XM134205 |
| COPS3 | GC17M017246 |
| SRBD1 | GC02M045388 |
| PPP1R13B | GC14M103733 |
| DYNLRB1 | GC20P034598 |
| GRIK1 | GC21M029536 |
| EDAR | GC02M108894 |
| NRXN3 | GC14P077980 |
| CPEB4 | GC05P173888 |
| SCIN | GC07P012570 |
| LRP12 | GC08M104489 |
| HERC5 | GC04P088457 |
| MIR7-2 | GC15P088611 |
| BDP1 | GC05P072416 |
| CCNI | GC04M077047 |
| MDC1-AS1 | GC06P076840 |
| NOXA1 | GC09P137423 |
| BRIX1 | GC05P034916 |
| AMD1 | GC06P110814 |
| MNAT1 | GC14P060734 |
| MIR605 | GC10P051299 |
| PLIN3 | GC19M004839 |
| WDR46 | GC06M061397 |
| RABIF | GC01M202878 |
| USP11 | GC0XP047232 |
| AGO3 | GC01P035991 |
| PPP2R5E | GC14M063371 |
| GRIA3 | GC0XP123184 |
| APOBEC3H | GC22P039097 |
| FIBP | GC11M084711 |
| RAMP3 | GC07P045163 |
| SESN3 | GC11M095782 |
| BOK-AS1 | GC02M241544 |
| UBE2A | GC0XP119574 |
| SCYL2 | GC12P100267 |
| PPAT | GC04M056393 |
| BPHL | GC06P003118 |
| XRN2 | GC20P021303 |
| SMIM31 | GC04P164755 |
| ZC3HAV1 | GC07M139044 |
| TERLR1 | GC05M001168 |
| ADAM1A | GC12P111899 |
| SHCBP1 | GC16M046578 |
| ZNF326 | GC01P089995 |
| GALR1 | GC18P077250 |
| MIR1247 | GC14M101560 |
| ADAMTS5 | GC21M026918 |
| RGS22 | GC08M099960 |
| MAGEA10 | GC0XM152133 |
| GPSM3 | GC06M061332 |
| ZNF582 | GC19M056375 |
| RMST | GC12P097431 |
| IL31 | GC12M122173 |
| CALU | GC07P128739 |
| EPB41L5 | GC02P120013 |
| PRAC2 | GC17P048720 |
| APH1A | GC01M150265 |
| KIDINS220 | GC02M008724 |
| MIR101-2 | GC09P004863 |
| TAF11 | GC06M061431 |
| FABP12 | GC08M081524 |
| NOX3 | GC06M155395 |
| SRRM1 | GC01P024631 |
| MIR1204 | GC08P127795 |
| RASSF10 | GC11P012990 |
| TUBG2 | GC17P042659 |
| UCHL3 | GC13P075550 |
| KLK9 | GC19M051002 |
| RTN1 | GC14M059595 |
| SPAG5 | GC17M028577 |
| ULBP3 | GC06M150062 |
| HSD17B12 | GC11P043717 |
| RHOF | GC12M121777 |
| SNAPIN | GC01P153660 |
| NEURL4 | GC17M007315 |
| SKA2 | GC17M059109 |
| SUPT16H | GC14M021351 |
| INSC | GC11P015127 |
| PLEKHM1 | GC17M045435 |
| CAPNS1 | GC19P051440 |
| UBE2E2 | GC03P023221 |
| RNPEP | GC01P201982 |
| LRRC8D | GC01P089821 |
| HS2ST1 | GC01P086914 |
| ENSA | GC01M151440 |
| SULT1A2 | GC16M028591 |
| SPANXC | GC0XM141241 |
| CNOT7 | GC08M017224 |
| UTP14A | GC0XP129906 |
| ERO1A | GC14M052640 |
| MAGED4B | GC0XM052061 |
| ABCF2 | GC07M151211 |
| COPG2 | GC07M130506 |
| MIR506 | GC0XM147230 |
| RAPGEF5 | GC07M022128 |
| GAS2 | GC11P022626 |
| FRMD6 | GC14P051489 |
| ZNF143 | GC11P009483 |
| KLK1 | GC19M050819 |
| VIRMA | GC08M094488 |
| ORM1 | GC09P114323 |
| PKNOX1 | GC21P042974 |
| LINC01426 | GC21P034745 |
| COPS6 | GC07P100088 |
| JPT2 | GC16P009870 |
| PSME1 | GC14P024136 |
| CHRNB2 | GC01P154568 |
| AGBL1 | GC15P112303 |
| PNOC | GC08P028316 |
| RABEP1 | GC17P005282 |
| RAB11FIP2 | GC10M118004 |
| PCNX3 | GC11P068999 |
| TENT4A | GC05P006713 |
| SLC27A4 | GC09P128340 |
| PGAM1 | GC10P097426 |
| YEATS2 | GC03P183698 |
| PEBP4 | GC08M022713 |
| PCDH17 | GC13P057630 |
| JDP2 | GC14P075427 |
| XXYLT1 | GC03M195068 |
| ULK4 | GC03M041247 |
| DHODH | GC16P072008 |
| H3C14 | GC01M151373 |
| TENM3 | GC04P181448 |
| KRT78 | GC12M052837 |
| CMTM6 | GC03M032499 |
| POLR2C | GC16P057462 |
| LARS1 | GC05M146114 |
| CA1 | GC08M085327 |
| NRM | GC06M061227 |
| EIF4A2 | GC03P186783 |
| GABRB3 | GC15M026543 |
| TMIGD2 | GC19M004292 |
| DNMT3L | GC21M044246 |
| XPR1 | GC01P180632 |
| NOX5 | GC15P092249 |
| PDZK1IP1 | GC01M047183 |
| DCAF4 | GC14P072926 |
| AP3M1 | GC10M074120 |
| GPR4 | GC19M045589 |
| FA2H | GC16M074712 |
| BRF2 | GC08M037820 |
| PPP2R2C | GC04M006322 |
| C18orf54 | GC18P054357 |
| IPO9 | GC01P201829 |
| MED28 | GC04P017617 |
| BOP1 | GC08M144262 |
| PDK3 | GC0XP024465 |
| VDAC2 | GC10P075210 |
| SMG1 | GC16M019128 |
| ZNF223 | GC19P044051 |
| CSRP1 | GC01M201484 |
| TWF1 | GC12M043793 |
| OVGP1 | GC01M111414 |
| FGD5-AS1 | GC03M019717 |
| H2AC6 | GC06P077462 |
| HAP1 | GC17M041717 |
| HCG18 | GC06M061211 |
| CDH19 | GC18M066501 |
| UTP6 | GC17M031860 |
| TRIM44 | GC11P035684 |
| CAPN6 | GC0XM111245 |
| MICAL1 | GC06M109444 |
| TIPARP | GC03P156673 |
| H2BC12L | GC21P043569 |
| DR1 | GC01P093345 |
| IPPK | GC09M092613 |
| MMP23B | GC01P001631 |
| STK11IP | GC02P219597 |
| KIF13A | GC06M017759 |
| S100A3 | GC01M153547 |
| CIAPIN1 | GC16M057428 |
| ARHGAP1 | GC11M084313 |
| PSPC1-AS2 | GC13P019675 |
| MORF4L1 | GC15P078810 |
| SF3B3 | GC16P070523 |
| MPZL2 | GC11M118253 |
| FHOD1 | GC16M067230 |
| NCAPH | GC02P096365 |
| PLAC1 | GC0XM134565 |
| MIR188 | GC0XP050003 |
| HACD3 | GC15P065530 |
| NUCKS1 | GC01M205712 |
| SLC18A1 | GC08M020144 |
| RPL27A | GC11P008682 |
| NR2C1 | GC12M095022 |
| SQLE | GC08P124998 |
| PPA1 | GC10M070202 |
| TECR | GC19P014504 |
| AP1G1 | GC16M071729 |
| YME1L1 | GC10M027110 |
| HYKK | GC15P078507 |
| H2BC13 | GC06M061060 |
| MYCBP | GC01M038862 |
| CEP70 | GC03M138494 |
| TNNC1 | GC03M052452 |
| TMEM116 | GC12M111894 |
| ZNF154 | GC19M062604 |
| ZC3H13 | GC13M045954 |
| GSTA2 | GC06M052750 |
| ATG12 | GC05M115828 |
| NBPF1 | GC01M016562 |
| PPP4C | GC16P039386 |
| SSR4 | GC0XP153793 |
| NCOA5 | GC20M046060 |
| ATP8A2 | GC13P025373 |
| EOGT | GC03M068975 |
| PGA4 | GC11P061222 |
| LINC00520 | GC14M055781 |
| GDI2 | GC10M005765 |
| MASP1 | GC03M187216 |
| BIK | GC22P043110 |
| UGT1A10 | GC02P233636 |
| MOS | GC08M056112 |
| HOXC9 | GC12P054901 |
| AJAP1 | GC01P004654 |
| HADH | GC04P107989 |
| FOXR1 | GC11P118971 |
| PYGB | GC20P025248 |
| GRHL1 | GC02P009951 |
| PHC2 | GC01M033418 |
| MIA3 | GC01P222618 |
| ADAMTS7 | GC15M078759 |
| CRY2 | GC11P046397 |
| DENND2D | GC01M111185 |
| RHCG | GC15M089471 |
| KXD1 | GC19P018557 |
| PDHB | GC03M058428 |
| IPO8 | GC12M030628 |
| CLEC4E | GC12M008535 |
| SYNCRIP | GC06M085607 |
| RGS9 | GC17P065332 |
| C19orf33 | GC19P038304 |
| SSH1 | GC12M108784 |
| NINL | GC20M025452 |
| RGS10 | GC10M119499 |
| MIR936 | GC10M104048 |
| IL36RN | GC02P120920 |
| DSCAM-AS1 | GC21P040383 |
| LINC01138 | GC01M148520 |
| DHX15 | GC04M024519 |
| CXXC4 | GC04M104468 |
| DLG2 | GC11M083455 |
| S1PR5 | GC19M010512 |
| GPAA1 | GC08P144082 |
| SAGE1 | GC0XP135889 |
| MYLK4 | GC06M002663 |
| USP18 | GC22P018149 |
| HSF2 | GC06P122399 |
| GPX8 | GC05P055160 |
| AZU1 | GC19P000825 |
| TRPV2 | GC17P016415 |
| PDSS2 | GC06M107152 |
| HCG22 | GC06P031053 |
| ATP6V1B1 | GC02P070935 |
| PTCD3 | GC02P086106 |
| TAF1L | GC09M032619 |
| KIF18A | GC11M028020 |
| DDX56 | GC07M044565 |
| MRGBP | GC20P063256 |
| CACNA2D3 | GC03P054156 |
| PIF1 | GC15M064815 |
| RPRD2 | GC01P150363 |
| POU3F1 | GC01M038066 |
| VSNL1 | GC02P017539 |
| ERH | GC14M069380 |
| PRR11 | GC17P059155 |
| HIPK1 | GC01P113929 |
| DDX59 | GC01M200594 |
| GMPS | GC03P155870 |
| MIR383 | GC08M014853 |
| FBXL2 | GC03P033277 |
| SHMT2 | GC12P057229 |
| LBX2-AS1 | GC02P074498 |
| HCG4 | GC06M061196 |
| MAGEC1 | GC0XP141905 |
| SSR1 | GC06M007268 |
| FAM189B | GC01M155248 |
| EGFL8 | GC06P076891 |
| PALM2AKAP2 | GC09P109499 |
| MAP7D1 | GC01P036155 |
| GAR1 | GC04P109815 |
| ZNF280B | GC22M022484 |
| HLA-K | GC06P076819 |
| NCCRP1 | GC19P039196 |
| H19-ICR | GC11P002009 |
| CSMD3 | GC08M112223 |
| FGL2 | GC07M077193 |
| MIR1205 | GC08P127960 |
| MIR323A | GC14P109066 |
| CCDC85B | GC11P065890 |
| DDX46 | GC05P134758 |
| MIR515-1 | GC19P053679 |
| SNX18 | GC05P054517 |
| HNRNPH3 | GC10P068331 |
| KLF8 | GC0XP055909 |
| TMEM80 | GC11P000695 |
| CDR1 | GC0XM140782 |
| ACACB | GC12P109116 |
| IDH3B | GC20M002658 |
| BLOC1S2 | GC10M100273 |
| ZKSCAN8 | GC06P028141 |
| SUZ12P1 | GC17P030709 |
| MCM3AP-AS1 | GC21P046229 |
| LIN54 | GC04M082909 |
| UPP1 | GC07P048088 |
| PSG1 | GC19M042866 |
| H1-10 | GC03M129989 |
| LINC01537 | GC11P072570 |
| UPF3B | GC0XM119805 |
| TNNT1 | GC19M055132 |
| GIP | GC17M048958 |
| LINC02231 | GC12M064903 |
| CDYL | GC06P004706 |
| UGP2 | GC02P063840 |
| SSBP1 | GC07P147868 |
| TRG-AS1 | GC07P038308 |
| ANKRD36B | GC02M097799 |
| MIR187 | GC18M035904 |
| ADCY7 | GC16P050495 |
| LAP3 | GC04P017674 |
| PCK2 | GC14P024094 |
| FMNL3 | GC12M049636 |
| ZNF830 | GC17P034961 |
| ESRP2 | GC16M068733 |
| ASXL2 | GC02M025733 |
| MEOX1 | GC17M043640 |
| FAAP20 | GC01M005183 |
| MIR216B | GC02M056000 |
| UBE2Q1 | GC01M154521 |
| GALR2 | GC17P076070 |
| GREM2 | GC01M240489 |
| RAB6C | GC02P129979 |
| NPTX1 | GC17M080466 |
| HIVEP2 | GC06M142751 |
| PCDH8 | GC13M052842 |
| FAM83A | GC08P123178 |
| PIP5K1B | GC09P068705 |
| UBAP1 | GC09P034179 |
| IL17RB | GC03P053855 |
| BLVRB | GC19M040447 |
| RRAD | GC16M066960 |
| IFIT2 | GC10P090732 |
| TACR3 | GC04M103586 |
| INTS4 | GC11M085124 |
| ZNF599 | GC19M034758 |
| MEOX2 | GC07M015617 |
| SNHG8 | GC04P118278 |
| DNAJC9 | GC10M073183 |
| ZKSCAN4 | GC06M061073 |
| LIPH | GC03M185506 |
| NAE1 | GC16M066803 |
| PYCR2 | GC01M225919 |
| AKR1C4 | GC10P005195 |
| LY6G6C | GC06M061313 |
| MISP | GC19P002144 |
| PCK1 | GC20P057561 |
| IL1RL2 | GC02P102186 |
| PAICS | GC04P056410 |
| MYOZ2 | GC04P119135 |
| PTPRR | GC12M070638 |
| FBXW2 | GC09M120751 |
| STAB1 | GC03P052495 |
| SLURP1 | GC08M142740 |
| HMGN2P46 | GC15P045511 |
| BARHL2 | GC01M090711 |
| DHRS2 | GC14P030871 |
| NUDT5 | GC10M012165 |
| RAB7B | GC01M205976 |
| S100A16 | GC01M153606 |
| ZSCAN31 | GC06M061075 |
| GNB4 | GC03M179397 |
| GTSE1 | GC22P046296 |
| SASS6 | GC01M100083 |
| FLACC1 | GC02M201387 |
| MIR500A | GC0XP050008 |
| CXXC5 | GC05P139647 |
| SNX2 | GC05P122774 |
| BTF3 | GC05P073498 |
| RAP1GDS1 | GC04P098261 |
| DNAJC2 | GC07M103312 |
| METTL16 | GC17M002405 |
| DCLK3 | GC03M036712 |
| BAGE | GC21U900396 |
| GJA10 | GC06P089894 |
| TGS1 | GC08P055773 |
| NLGN3 | GC0XP071144 |
| PSPH | GC07M056010 |
| SLC24A5 | GC15P048120 |
| CENPH | GC05P069189 |
| TCEA3 | GC01M023382 |
| NELFE | GC06M031952 |
| DHX36 | GC03M154272 |
| TPRG1 | GC03P188948 |
| PIGK | GC01M077088 |
| UBE2J2 | GC01M005132 |
| ALG13 | GC0XP111665 |
| GABPB2 | GC01P151070 |
| SERHL | GC22P042500 |
| NWD1 | GC19P016719 |
| PHF2 | GC09P093576 |
| MIR208A | GC14M023388 |
| MMP20 | GC11M102576 |
| EN1 | GC02M118842 |
| SEC14L1 | GC17P077086 |
| SEMA4C | GC02M096859 |
| COASY | GC17P042561 |
| TRIM66 | GC11M008612 |
| AP2A2 | GC11P000924 |
| LINC00355 | GC13M063838 |
| S100A13 | GC01M153618 |
| MIR622 | GC13P090231 |
| MMP28 | GC17M035756 |
| SEL1L | GC14M081471 |
| ABCB10 | GC01M229516 |
| NOC3L | GC10M094333 |
| NDST1 | GC05P150484 |
| MIR493 | GC14P109089 |
| UBE2L6 | GC11M084396 |
| INSIG2 | GC02P118088 |
| IL17D | GC13P020702 |
| MRPS34 | GC16M001771 |
| AOC1 | GC07P150824 |
| MAEL | GC01P166922 |
| ARPC4 | GC03P009792 |
| PNPLA1 | GC06P077006 |
| HORMAD2 | GC22P030080 |
| ADD2 | GC02M070626 |
| ZNF185 | GC0XP152898 |
| MTX1 | GC01P155208 |
| RAB11FIP1 | GC08M037858 |
| TJP3 | GC19P003708 |
| EPN2 | GC17P019215 |
| ZNF532 | GC18P058862 |
| POLR2F | GC22P037952 |
| DOK4 | GC16M057505 |
| GABRB1 | GC04P046949 |
| ATP5F1D | GC19P002208 |
| GNB1L | GC22M019783 |
| ZBTB7B | GC01P155002 |
| SSBP3 | GC01M054225 |
| MMAB | GC12M109553 |
| BTN3A3 | GC06P076627 |
| MAGEA5P | GC0XM152119 |
| LINC02098 | GC11P128210 |
| SKA3 | GC13M021153 |
| RSRC2 | GC12M122503 |
| HSCB | GC22P033666 |
| CLDN12 | GC07P090383 |
| FADS3 | GC11M061873 |
| DBF4 | GC07P087877 |
| GRIK3 | GC01M036795 |
| PPIH | GC01P042657 |
| SLC25A6 | GC0XM001386 |
| ST3GAL6 | GC03P098732 |
| ANKRD65 | GC01M001418 |
| GZMM | GC19P000544 |
| BUD13 | GC11M116749 |
| PTPRG-AS1 | GC03M062248 |
| CRHR2 | GC07M030651 |
| NOC2L | GC01M005110 |
| MIR369 | GC14P109071 |
| CDH12 | GC05M021786 |
| IARS1 | GC09M092211 |
| CDK19 | GC06M110609 |
| BAG2 | GC06P057172 |
| WFDC21P | GC17M060085 |
| SLC28A1 | GC15P084884 |
| DGUOK-AS1 | GC02M073947 |
| IKZF2 | GC02M213001 |
| SCAMP3 | GC01M155255 |
| ABRAXAS2 | GC10P124802 |
| IGF2BP2-AS1 | GC03P185712 |
| DNASE1L1 | GC0XM154401 |
| BHMT | GC05P079111 |
| ENSG00000272221 | GC06M061273 |
| FAT3 | GC11P092224 |
| FYCO1 | GC03M045917 |
| CERS6 | GC02P168455 |
| MFF | GC02P227325 |
| MKRN1 | GC07M140453 |
| ALDH1B1 | GC09P038392 |
| MAP4K2 | GC11M084629 |
| AKAP4 | GC0XM050190 |
| SETD6 | GC16P058514 |
| STIP1 | GC11P064284 |
| HHLA2 | GC03P108296 |
| CSGALNACT1 | GC08M019404 |
| GPANK1 | GC06M061308 |
| DLX3 | GC17M049990 |
| MGRN1 | GC16P009970 |
| NRBP1 | GC02P027427 |
| DCLRE1A | GC10M113834 |
| DLD | GC07P107890 |
| LYNX1 | GC08M142966 |
| ADCYAP1R1 | GC07P031058 |
| MIR508 | GC0XM147236 |
| SIVA1 | GC14P108830 |
| ARHGEF5 | GC07P144355 |
| ERVK-6 | GC07U903184 |
| KRT81 | GC12M052286 |
| HAS2-AS1 | GC08P121639 |
| BAIAP2-DT | GC17M081097 |
| FBXO7 | GC22P032474 |
| UBE2M | GC19M058555 |
| LSM4 | GC19M018306 |
| THORLNC | GC02M118133 |
| SLC24A4 | GC14P092322 |
| MYOT | GC05P137867 |
| DTNB | GC02M025378 |
| PLB1 | GC02P028460 |
| OPRL1 | GC20P064080 |
| MYLIP | GC06P016129 |
| RABGGTA | GC14M024265 |
| DIXDC1 | GC11P111927 |
| SNRPD1 | GC18P021612 |
| RGS16 | GC01M182598 |
| LINC00365 | GC13M030103 |
| PLPP4 | GC10P120457 |
| MAP1S | GC19P050937 |
| ZNF469 | GC16P088382 |
| LINC00173 | GC12P116533 |
| SEMA5B | GC03M122909 |
| HLA-U | GC06P076820 |
| FTH1P3 | GC02M027392 |
| DFFB | GC01P003797 |
| SERPINB8 | GC18P063969 |
| ZNF391 | GC06P027374 |
| GRM2 | GC03P051707 |
| UBE2D2 | GC05P139526 |
| FGF12 | GC03M192139 |
| GTF2H2 | GC05M071032 |
| ADAMTS15 | GC11P130448 |
| GALNT7 | GC04P173168 |
| P4HA1 | GC10M073007 |
| IPO5 | GC13P097953 |
| RHEBL1 | GC12M049064 |
| ACP2 | GC11M084325 |
| CLEC4G | GC19M007728 |
| ZBED9 | GC06M061090 |
| TCF25 | GC16P089873 |
| LARP7 | GC04P112636 |
| ZNF16 | GC08M144930 |
| DPP7 | GC09M137381 |
| HOXD10 | GC02P176108 |
| RNF20 | GC09P101533 |
| ME1 | GC06M083210 |
| IPO7 | GC11P009384 |
| SUOX | GC12P055997 |
| DPP3 | GC11P069101 |
| TSGA10 | GC02M098997 |
| SSX4 | GC0XP048383 |
| MKNK1 | GC01M046557 |
| WBP2 | GC17M075845 |
| MIR4732 | GC17M033778 |
| P2RX3 | GC11P057356 |
| CCDC144NL-AS1 | GC17P050745 |
| CHMP4B | GC20P033984 |
| ZC3H7A | GC16M011750 |
| ZNF655 | GC07P099673 |
| KIN | GC10M007750 |
| ZNF418 | GC19M057921 |
| CTDSP1 | GC02P218398 |
| ADRA2B | GC02M096112 |
| SOX12 | GC20P000325 |
| RAB27B | GC18P054717 |
| POU6F2-AS2 | GC07M038979 |
| VARS2 | GC06P076846 |
| SPATA22 | GC17M003440 |
| KLKB1 | GC04P186208 |
| DNM3OS | GC01M172217 |
| UBE2B | GC05P134371 |
| HCN1 | GC05M045260 |
| CENPP | GC09P092325 |
| PELO | GC05P052787 |
| NDUFAF5 | GC20P013879 |
| IFNL3 | GC19M039243 |
| PSPC1 | GC13M019674 |
| GAGE1 | GC0XP050354 |
| KIFAP3 | GC01M169921 |
| C6orf47 | GC06M061307 |
| CEP19 | GC03M196706 |
| GTPBP1 | GC22P038705 |
| TRIM2 | GC04P153152 |
| LINC00467 | GC01P211382 |
| ENO3 | GC17P004948 |
| AFTPH | GC02P064524 |
| UFL1 | GC06P096521 |
| LY6G5C | GC06M031676 |
| POFUT2 | GC21M045263 |
| STAMBP | GC02P073828 |
| CCIN | GC09P036169 |
| TMEFF1 | GC09P100473 |
| NR2F1-AS1 | GC05M093409 |
| SLC30A9 | GC04P041992 |
| GPR182 | GC12P057136 |
| ADAM3A | GC08M039427 |
| ADH1A | GC04M099276 |
| CFHR2 | GC01P196943 |
| EIF5 | GC14P103333 |
| VDAC3 | GC08P042392 |
| GGCT | GC07M030496 |
| GRAMD4 | GC22P046576 |
| HNRNPAB | GC05P178204 |
| TMEM219 | GC16P039376 |
| GFPT2 | GC05M180300 |
| APCDD1 | GC18P010454 |
| NUSAP1 | GC15P041735 |
| ZBTB12 | GC06M031899 |
| TAB3 | GC0XM030876 |
| MIR874 | GC05M137647 |
| OAZ1 | GC19P002269 |
| H2BC5 | GC06P077466 |
| MRPL44 | GC02P223957 |
| HELB | GC12P066302 |
| PNISR | GC06M099398 |
| SENP5 | GC03P196869 |
| YTHDC1 | GC04M068310 |
| GLRX2 | GC01M193065 |
| HPD | GC12M121839 |
| LBH | GC02P030231 |
| MIR26A2 | GC12M057824 |
| SPANXA1 | GC0XM141583 |
| GABRG3 | GC15P026971 |
| G0S2 | GC01P209675 |
| SLC47A1 | GC17P019495 |
| HECTD3 | GC01M045002 |
| HOXC10 | GC12P054898 |
| TSPYL5 | GC08M097273 |
| INPP5A | GC10P132537 |
| TRIM47 | GC17M075874 |
| MIDEAS | GC14M073817 |
| BEX1 | GC0XM103063 |
| GTF2A1 | GC14M081175 |
| MIR33B | GC17M017813 |
| C1orf109 | GC01M037681 |
| ING2 | GC04P183504 |
| HLA-J | GC06P076823 |
| TIMM50 | GC19P039480 |
| TUSC8 | GC13M044400 |
| CLTCL1 | GC22M019266 |
| NR2C2AP | GC19M019201 |
| GTDC1 | GC02M143938 |
| SLC27A3 | GC01P153850 |
| PAXBP1 | GC21M032734 |
| HTR7 | GC10M090740 |
| RAB21 | GC12P071754 |
| EXOC3 | GC05P000443 |
| APLP2 | GC11P130069 |
| RASSF4 | GC10P044959 |
| MRPS27 | GC05M072219 |
| PGS1 | GC17P078378 |
| OXR1 | GC08P106271 |
| PFDN1 | GC05M140291 |
| STMN3 | GC20M063639 |
| SPRR1A | GC01P152984 |
| C10orf99 | GC10P084173 |
| ARIH1 | GC15P072474 |
| BTN2A1 | GC06P026457 |
| ATP6V1C1 | GC08P103038 |
| TDGF1P3 | GC0XP110520 |
| STXBP4 | GC17P054968 |
| CHAC1 | GC15P040942 |
| TARBP1 | GC01M234391 |
| STK38L | GC12P027243 |
| HOXB6 | GC17M048686 |
| SHC3 | GC09M089005 |
| MT1A | GC16P056638 |
| OTUB1 | GC11P063985 |
| RPL3P2 | GC06P031280 |
| KLHL11 | GC17M041873 |
| PFDN5 | GC12P053295 |
| LINC02156 | GC12P047416 |
| DSCR8 | GC21P038121 |
| PDIA4 | GC07M149003 |
| NGB | GC14M077265 |
| DLX1 | GC02P172084 |
| ABR | GC17M001003 |
| MIR512-1 | GC19P062493 |
| ATP6V1G2 | GC06M061301 |
| SARNP | GC12M055752 |
| RHOBTB3 | GC05P095713 |
| TAS2R38 | GC07M141972 |
| MIR1208 | GC08P128150 |
| PLAAT4 | GC11P063660 |
| CLTA | GC09P036190 |
| MIR449B | GC05M055172 |
| RTCB | GC22M032387 |
| CYP2R1 | GC11M014877 |
| MARCHF8 | GC10M046239 |
| NLRX1 | GC11P119166 |
| TKFC | GC11P061334 |
| SMYD4 | GC17M001779 |
| RBM28 | GC07M128381 |
| UBP1 | GC03M033404 |
| DNAJC18 | GC05M139408 |
| KRT84 | GC12M052377 |
| REXO4 | GC09M133406 |
| LRRC74B | GC22P033350 |
| HMGCS2 | GC01M119747 |
| CLDN20 | GC06P155264 |
| PSORS1C3 | GC06M061253 |
| GNL1 | GC06M030541 |
| XCR1 | GC03M046016 |
| SYF2 | GC01M025222 |
| TMC2 | GC20P002536 |
| PAQR3 | GC04M078887 |
| HLA-W | GC06P076817 |
| LINC00951 | GC06M040344 |
| U2SURP | GC03P142964 |
| FAM124B | GC02M224378 |
| CERS3 | GC15M114806 |
| RAB22A | GC20P058309 |
| CLC | GC19M061844 |
| STAG1 | GC03M136336 |
| PHB1P1 | GC06P150047 |
| SUMO1P3 | GC01P160317 |
| FOXJ2 | GC12P008032 |
| SACM1L | GC03P046263 |
| MFAP4 | GC17M019383 |
| VPS13D | GC01P012231 |
| GFUS | GC08M143657 |
| NNAT | GC20P037521 |
| AGPAT1 | GC06M032168 |
| RNF144B | GC06P018560 |
| RABL6 | GC09P136807 |
| ST7L | GC01M112523 |
| HUS1B | GC06M000655 |
| NELL1 | GC11P020669 |
| SNTA1 | GC20M033407 |
| ATP6V0C | GC16P002513 |
| MIR518B | GC19P062500 |
| WAPL | GC10M086436 |
| TESPA1 | GC12M054949 |
| TMED10 | GC14M075132 |
| CCL28 | GC05M043356 |
| RAET1E | GC06M149883 |
| DMRTA1 | GC09P022436 |
| GTF3C4 | GC09P132671 |
| ACCS | GC11P044045 |
| ACTL8 | GC01P017756 |
| SNRPA1 | GC15M101281 |
| DDX24 | GC14M094048 |
| RPAIN | GC17P005419 |
| UQCRC2 | GC16P022094 |
| ELP3 | GC08P028089 |
| DCBLD1 | GC06P117453 |
| SIM1 | GC06M100386 |
| PCOLCE | GC07P100602 |
| LPAR4 | GC0XP078747 |
| CMSS1 | GC03P099817 |
| NTSR2 | GC02M011649 |
| KAZN | GC01P013893 |
| ASIC1 | GC12P050057 |
| CCDC86 | GC11P060957 |
| ZNF184 | GC06M061011 |
| BCL2L10 | GC15M079646 |
| CEP76 | GC18M023296 |
| USP13 | GC03P179652 |
| KRT6C | GC12M052468 |
| GPR132 | GC14M105049 |
| P4HA3 | GC11M074235 |
| CYP21A1P | GC06P032005 |
| TNN | GC01P175067 |
| DRD5 | GC04P009783 |
| GPR55 | GC02M230907 |
| SEC62 | GC03P169966 |
| CRYBG1 | GC06P106361 |
| RETNLB | GC03M108743 |
| RNF144A | GC02P006917 |
| VTI1B | GC14M067647 |
| CFAP20 | GC16M058113 |
| STK19B | GC06P032013 |
| MEX3A | GC01M156072 |
| STARD7 | GC02M096184 |
| EDARADD | GC01P236348 |
| THSD1 | GC13M052377 |
| CYB5D2 | GC17P004143 |
| BRD8 | GC05M138150 |
| MRPL43 | GC10M100969 |
| PPM1A | GC14P060245 |
| CDH7 | GC18P065750 |
| TEX10 | GC09M100302 |
| DAGLA | GC11P061680 |
| POLR2J | GC07M102473 |
| POLR2H | GC03P184361 |
| CTBP1-AS | GC04P001210 |
| ZNF131 | GC05P044138 |
| CATSPER2 | GC15M043628 |
| FBXO32 | GC08M123500 |
| AURKAIP1 | GC01M001373 |
| SMG9 | GC19M043727 |
| SCARA5 | GC08M027869 |
| MIR1246 | GC02M176600 |
| ERP44 | GC09M099979 |
| CTSV | GC09M097029 |
| HMGN4 | GC06P026538 |
| MTREX | GC05P055308 |
| ACP6 | GC01M147630 |
| DDX51 | GC12M132136 |
| SCEL | GC13P077535 |
| TSPAN15 | GC10P069451 |
| PAPSS1 | GC04M107590 |
| AKR1D1 | GC07P138003 |
| KIFBP | GC10P068990 |
| SLC35E2A | GC01M005170 |
| UGGT1 | GC02P128091 |
| DUSP16 | GC12M012473 |
| ENSG00000285708 | GC03M070959 |
| CAPRIN2 | GC12M030709 |
| GORAB | GC01P170501 |
| DIPK2A | GC03P143973 |
| ARMC9 | GC02P231198 |
| MRPL38 | GC17M075900 |
| FLNC-AS1 | GC07M128851 |
| HHATL | GC03M042698 |
| AHSA1 | GC14P077457 |
| D2HGDH | GC02P241734 |
| ZNF592 | GC15P112259 |
| C9 | GC05M039320 |
| SLITRK1 | GC13M083877 |
| PGRMC2 | GC04M128269 |
| RGS7 | GC01M240767 |
| GAN | GC16P081319 |
| AKAP11 | GC13P042272 |
| SDR9C7 | GC12M056923 |
| TRIM11 | GC01M228393 |
| LRG1 | GC19M004681 |
| IFIT1 | GC10P090733 |
| RIN1 | GC11M084738 |
| IPO4 | GC14M024181 |
| GBAP1 | GC01M155213 |
| N6AMT1 | GC21M028441 |
| ANKRD27 | GC19M032597 |
| HCAR1 | GC12M122726 |
| LRRC8B | GC01P089525 |
| NUAK2 | GC01M205302 |
| DNAJB2 | GC02P219279 |
| ANXA2R | GC05M043385 |
| SUGP1 | GC19M019276 |
| TFEC | GC07M115935 |
| SAPCD1 | GC06P076881 |
| LHX6 | GC09M122202 |
| BMF | GC15M040087 |
| RNMT | GC18P016786 |
| GLIS1 | GC01M053507 |
| HEYL | GC01M039623 |
| CALML3 | GC10P005556 |
| RAB3D | GC19M011322 |
| ATG4A | GC0XP108091 |
| GLYAT | GC11M084412 |
| XCL2 | GC01M168510 |
| DDX19B | GC16P070289 |
| CYP2J2 | GC01M059893 |
| RIC8A | GC11P000207 |
| SERTAD1 | GC19M040421 |
| PPP3R1 | GC02M068143 |
| ITIH3 | GC03P052794 |
| OLFM3 | GC01M101802 |
| CLPTM1 | GC19P044954 |
| ITIH5 | GC10M007559 |
| HPSE2 | GC10M098457 |
| CPVL | GC07M028995 |
| PLRG1 | GC04M154534 |
| SLC9A2 | GC02P102620 |
| SUB1 | GC05P032577 |
| KCNS3 | GC02P017877 |
| LINC02154 | GC0XM013266 |
| DUSP7 | GC03M052048 |
| PRKRA | GC02M178431 |
| FABP6 | GC05P160187 |
| KHK | GC02P027086 |
| GPD1L | GC03P032123 |
| OLFML3 | GC01P113979 |
| UBE2G2 | GC21M044768 |
| SMG7 | GC01P183441 |
| SPNS2 | GC17P004498 |
| CTDSP2 | GC12M057819 |
| TMCC3 | GC12M094567 |
| ZBTB4 | GC17M007459 |
| DTX3L | GC03P122564 |
| LRMDA | GC10P075432 |
| RNPS1 | GC16M002253 |
| P2RX5 | GC17M003672 |
| ITIH1 | GC03P052777 |
| WBP11 | GC12M014784 |
| CLDN15 | GC07M101232 |
| CLNS1A | GC11M085114 |
| KRTCAP3 | GC02P027442 |
| NDRG3 | GC20M036651 |
| IL36B | GC02M113022 |
| ENSG00000237669 | GC06M061796 |
| FNDC1 | GC06P160301 |
| DHX33 | GC17M005440 |
| GPSM1 | GC09P136327 |
| NRDC | GC01M051790 |
| RARS2 | GC06M087514 |
| USP17L2 | GC08M012545 |
| GTF2A1L | GC02P048617 |
| UPK1B | GC03P119173 |
| MARVELD2 | GC05P069415 |
| MIS12 | GC17P005486 |
| SESN1 | GC06M108986 |
| LMAN1 | GC18M059327 |
| P2RY4 | GC0XM070258 |
| NSFL1C | GC20M001442 |
| GSTA4 | GC06M052977 |
| GOLGA4 | GC03P037243 |
| EIF4E2 | GC02P232550 |
| H3C7 | GC06M061833 |
| USHBP1 | GC19M017249 |
| MIR431 | GC14P108751 |
| SNX14 | GC06M085505 |
| UPF3A | GC13P114281 |
| MATN1 | GC01M030711 |
| TP53AIP1 | GC11M128934 |
| RAB32 | GC06P146543 |
| MAGEA2B | GC0XP152714 |
| P4HTM | GC03P049520 |
| HAL | GC12M095972 |
| LAD1 | GC01M201373 |
| ACAP2 | GC03M195274 |
| TNNI3K | GC01P074235 |
| EXOSC4 | GC08P144081 |
| PARG | GC10M049818 |
| CTAGE4 | GC07P144183 |
| RAB37 | GC17P074671 |
| PTTG1IP | GC21M044849 |
| SLTM | GC15M058879 |
| LMO3 | GC12M016548 |
| CLEC2B | GC12M019783 |
| CKM | GC19M045306 |
| ROPN1L | GC05P010441 |
| RNGTT | GC06M088609 |
| COPS7A | GC12P018550 |
| MED17 | GC11P093784 |
| COL19A1 | GC06P069866 |
| ZNF536 | GC19P051259 |
| UBE2Q2 | GC15P075843 |
| BEX4 | GC0XP103215 |
| LRRC26 | GC09M137384 |
| TMEM14B | GC06P010747 |
| HILPDA | GC07P131409 |
| PLIN1 | GC15M089664 |
| LINC02157 | GC15P112808 |
| PWWP3A | GC19P002214 |
| NOL4 | GC18M033851 |
| ATP2C2 | GC16P084368 |
| MED16 | GC19M004323 |
| SAA4 | GC11M018234 |
| HNRNPA0 | GC05M137750 |
| PDE10A | GC06M165327 |
| TPPP3 | GC16M067389 |
| ENSG00000199332 | GC06M061276 |
| CD300C | GC17M074544 |
| KLHL6 | GC03M183487 |
| ST7 | GC07P117071 |
| MIR760 | GC01P093846 |
| HS3ST3A1 | GC17M013494 |
| MYT1 | GC20P064918 |
| MT1X | GC16P056966 |
| ENTPD5 | GC14M073958 |
| USP34 | GC02M061187 |
| HOXC4 | GC12P054016 |
| CIAO2B | GC16M066969 |
| HTR2C | GC0XP114584 |
| ZNF471 | GC19P062627 |
| PGM5-AS1 | GC09M068355 |
| SOX15 | GC17M007656 |
| MIR153-1 | GC02M219294 |
| INSRR | GC01M156840 |
| H3C8 | GC06M061834 |
| KPNA5 | GC06P116681 |
| PTCSC2 | GC09M097699 |
| CDC42BPG | GC11M064823 |
| MIR194-1 | GC01M220118 |
| RIOK3 | GC18P023452 |
| KRT83 | GC12M052314 |
| COL23A1 | GC05M178237 |
| ZNF366 | GC05M072907 |
| SLC41A2 | GC12M104802 |
| IFI6 | GC01M027666 |
| CCDC34 | GC11M027327 |
| PTCSC3 | GC14M036139 |
| VPS26A | GC10P069123 |
| FMO2 | GC01P171185 |
| IRF2BPL | GC14M077024 |
| OVOL1 | GC11P065787 |
| GTF2H3 | GC12P123633 |
| MSANTD3 | GC09P100427 |
| HIPK3 | GC11P033301 |
| MICD | GC06M061206 |
| SMC6 | GC02M017663 |
| MIR421 | GC0XM074218 |
| GEM | GC08M094249 |
| JPX | GC0XP073996 |
| AHCYL2 | GC07P129225 |
| N4BP2L1 | GC13M036002 |
| C1GALT1 | GC07P007156 |
| TMEM123 | GC11M102396 |
| TMEM184B | GC22M056092 |
| COPS4 | GC04P083034 |
| KRT10-AS1 | GC17P050642 |
| MG828730-053 | GC06M062286 |
| HTR6 | GC01P019666 |
| NOMO2 | GC16M019114 |
| TRIM16 | GC17M015627 |
| ALAS1 | GC03P052198 |
| WIZ | GC19M015419 |
| VPS9D1-AS1 | GC16P089711 |
| RMDN3 | GC15M040735 |
| SYT4 | GC18M043267 |
| MAGEL2 | GC15M023643 |
| DUSP2 | GC02M097760 |
| QPCTL | GC19P045692 |
| DDI1 | GC11P104036 |
| KCNJ12 | GC17P049578 |
| GRM8 | GC07M126438 |
| MIR638 | GC19P010719 |
| HOMER2 | GC15M087760 |
| SOCS2-AS1 | GC12M093504 |
| BHLHE23 | GC20M063005 |
| SERPINA5 | GC14P094563 |
| MRPL20 | GC01M001401 |
| PNRC2 | GC01P024397 |
| SPAG4 | GC20P035615 |
| AFF2 | GC0XP148500 |
| ARL6IP4 | GC12P122980 |
| JPH4 | GC14M023568 |
| SLC35E2B | GC01M001659 |
| POLR2G | GC11P062773 |
| VPS37A | GC08P017246 |
| CSTF2 | GC0XP100820 |
| DIAPH2 | GC0XP096684 |
| TRAPPC1 | GC17M007930 |
| CRIP2 | GC14P105472 |
| GSTZ1 | GC14P077320 |
| MIR1179 | GC15P088608 |
| H2AC12 | GC06P077461 |
| B3GNT2 | GC02P062196 |
| MTCH2 | GC11M047604 |
| CIAO1 | GC02P096281 |
| SLC44A3 | GC01P094820 |
| CLDN9 | GC16P003012 |
| IFITM2 | GC11P000300 |
| MFNG | GC22M037469 |
| GABRB2 | GC05M161288 |
| TRIM7 | GC05M181193 |
| KYNU | GC02P142877 |
| TMEM87B | GC02P121604 |
| GOLIM4 | GC03M168008 |
| UQCRC1 | GC03M048598 |
| MIR662 | GC16P009837 |
| NAA40 | GC11P063938 |
| ADAMTS14 | GC10P070672 |
| SEC23IP | GC10P119892 |
| FAM50A | GC0XP154445 |
| MAGEA8 | GC0XP149881 |
| MGST1 | GC12P016347 |
| BHLHE22 | GC08P064580 |
| SPDL1 | GC05P169583 |
| SAP130 | GC02M128201 |
| QPCT | GC02P037344 |
| SLC25A28 | GC10M099610 |
| MAGED4 | GC0XP052184 |
| RNF34 | GC12P121400 |
| ASIC2 | GC17M033013 |
| ENDOV | GC17P080415 |
| GMFG | GC19M039328 |
| ZNF74 | GC22P020394 |
| SERPINB7 | GC18P063752 |
| GMPR | GC06P016238 |
| SLC1A6 | GC19M014921 |
| DIO1 | GC01P053891 |
| GPAT4 | GC08P041577 |
| ENSG00000227775 | GC01P003397 |
| SUV39H2 | GC10P014878 |
| DLX6 | GC07P097005 |
| LEO1 | GC15M079857 |
| CCZ1 | GC07P005898 |
| CELSR3 | GC03M048641 |
| MAS1 | GC06P160324 |
| CMAS | GC12P022046 |
| PHLDA3 | GC01M201467 |
| EEF1E1 | GC06M008073 |
| NRN1 | GC06M005997 |
| FUT5 | GC19M005865 |
| PIGF | GC02M046580 |
| AQP8 | GC16P027632 |
| PCNP | GC03P101574 |
| ADAMTS7P3 | GC15P077976 |
| GPR158 | GC10P025174 |
| PMPCB | GC07P103297 |
| SHQ1 | GC03M072725 |
| MIR411 | GC14P109084 |
| KCNC4 | GC01P110316 |
| IFNG-AS1 | GC12P067989 |
| NFE2L1 | GC17P051074 |
| SURF2 | GC09P133980 |
| NAF1 | GC04M163109 |
| SNX5 | GC20M018110 |
| MIR1284 | GC03M071541 |
| MN298114-181 | GC06M062298 |
| RGMA | GC15M093035 |
| AKR7L | GC01M019265 |
| CYC1 | GC08P144095 |
| POLN | GC04M002075 |
| ZFAND6 | GC15P080059 |
| ZNF7 | GC08P144982 |
| EEF1B2 | GC02P206159 |
| RHBDL2 | GC01M038885 |
| SMKR1 | GC07P129502 |
| RUBCN | GC03M197985 |
| SNTG2 | GC02P000942 |
| MYBPC1 | GC12P101568 |
| RND1 | GC12M048857 |
| UPF2 | GC10M011920 |
| CCDC148 | GC02M158171 |
| ZNF564 | GC19M012528 |
| PCDH20 | GC13M061409 |
| MAGEF1 | GC03M184710 |
| SLX1B | GC16P029454 |
| RCN2 | GC15P076931 |
| AP3S1 | GC05P115841 |
| MIR612 | GC11P068838 |
| ZNF417 | GC19M062614 |
| DDX19A | GC16P070346 |
| UCHL5 | GC01M193012 |
| SNRPB2 | GC20P016730 |
| INHBE | GC12P057452 |
| ACAA2 | GC18M049782 |
| ITPKA | GC15P041493 |
| CNFN | GC19M042387 |
| MIR410 | GC14P109083 |
| TSC22D4 | GC07M100463 |
| MIR1290 | GC01M018897 |
| GNL3L | GC0XP054531 |
| HMGN5 | GC0XM081113 |
| CINP | GC14M102342 |
| SPATA5 | GC04P122923 |
| FAM83A-AS1 | GC08M123201 |
| CHCHD6 | GC03P126704 |
| TUT7 | GC09M091435 |
| MIR876 | GC09M028855 |
| FHL3 | GC01M038065 |
| SPATA9 | GC05M095652 |
| CENPK | GC05M065517 |
| L2HGDH | GC14M050237 |
| CYTL1 | GC04M005016 |
| SRXN1 | GC20M000647 |
| PRDM4 | GC12M107732 |
| TMPRSS11D | GC04M067820 |
| MIR593 | GC07P128081 |
| GCNT4 | GC05M075025 |
| MIR939 | GC08M144394 |
| AP5M1 | GC14P057268 |
| MGAM | GC07P147778 |
| HIKESHI | GC11P086303 |
| POLR2D | GC02M128192 |
| TSC22D2 | GC03P150408 |
| GSTT2 | GC22P023980 |
| SLC35A5 | GC03P112561 |
| COPRS | GC17M031851 |
| C17orf49 | GC17P007014 |
| ENSG00000230521 | GC06M061197 |
| CMTM3 | GC16P066629 |
| FFAR3 | GC19P052502 |
| ZBTB2 | GC06M151364 |
| CYB5R4 | GC06P083859 |
| SGTA | GC19M002754 |
| EXOSC1 | GC10M097435 |
| DEF8 | GC16P091065 |
| ATG101 | GC12P052069 |
| SEC11A | GC15M084669 |
| ST6GALNAC1 | GC17M076624 |
| LCN1 | GC09P135521 |
| CCDC134 | GC22P041800 |
| CALML5 | GC10M005498 |
| GNPDA1 | GC05M141991 |
| FLJ22447 | GC14P061570 |
| SPRR2B | GC01M153070 |
| RASSF6 | GC04M073571 |
| BDH2 | GC04M103077 |
| B4GAT1 | GC11M066345 |
| MXD4 | GC04M002397 |
| HLA-V | GC06P076810 |
| EXOC1 | GC04P055853 |
| RGS20 | GC08P053851 |
| MUC22 | GC06P031005 |
| ETNK1 | GC12P022625 |
| EPGN | GC04P074309 |
| UAP1 | GC01P162561 |
| MIR301B | GC22P033380 |
| RGS11 | GC16M000268 |
| SUPT20H | GC13M037009 |
| H2AC13 | GC06P076681 |
| HLA-S | GC06M031381 |
| KCTD5 | GC16P002682 |
| RNF135 | GC17P049803 |
| CLP1 | GC11P057648 |
| APOBEC1 | GC12M007649 |
| RALGAPB | GC20P038472 |
| VLDLR-AS1 | GC09M002411 |
| SLC39A5 | GC12P057092 |
| ODF1 | GC08P102551 |
| RNF157 | GC17M076142 |
| ADAMTS6 | GC05M065148 |
| ZSCAN12 | GC06M061076 |
| NOM1 | GC07P156949 |
| ADAP1 | GC07M000897 |
| ARHGEF37 | GC05P149551 |
| NKX2-3 | GC10P099532 |
| NOL3 | GC16P067204 |
| ZNF394 | GC07M099475 |
| HSPBP1 | GC19M055262 |
| KRT76 | GC12M052768 |
| ABI2 | GC02P203327 |
| KLF16 | GC19M001852 |
| ABCG4 | GC11P119982 |
| RFFL | GC17M035006 |
| CLIC3 | GC09M136994 |
| MIR548C | GC12P064622 |
| TMEM240 | GC01M005152 |
| AANAT | GC17P076453 |
| TTLL11 | GC09M121816 |
| IKBKB-DT | GC08M042236 |
| ZNF410 | GC14P073886 |
| ZNF382 | GC19P051474 |
| LOC117134604 | GC21P041507 |
| HLA-DQB1-AS1 | GC06P032659 |
| WFIKKN2 | GC17P050834 |
| HCRTR1 | GC01P031587 |
| LINC01564 | GC06P077197 |
| RHBDL1 | GC16P009829 |
| NOCT | GC04P139016 |
| NSMCE4A | GC10M121957 |
| GABRA5 | GC15P026866 |
| MIR944 | GC03P189829 |
| CADM3-AS1 | GC01M159193 |
| TBC1D3D | GC17P038003 |
| GAS2L3 | GC12P100573 |
| LINC01535 | GC19P037251 |
| NR2F6 | GC19M017231 |
| MTNR1A | GC04M186533 |
| FAM169A | GC05M074777 |
| UBE2D3P3 | GC01P150800 |
| PIK3IP1 | GC22M031281 |
| PCF11 | GC11P083156 |
| ARF3 | GC12M049343 |
| GTF2IRD2P1 | GC07M073242 |
| C1QL4 | GC12M049332 |
| PCMT1 | GC06P149749 |
| GBP2 | GC01M089106 |
| MIR205HG | GC01P209661 |
| FTHL17 | GC0XM030999 |
| SEPTIN1 | GC16M030378 |
| MMRN2 | GC10M086935 |
| TDO2 | GC04P155854 |
| ALDOC | GC17M033773 |
| IFITM4P | GC06M061191 |
| EPHA10 | GC01M037713 |
| USP54 | GC10M073497 |
| CAMTA2 | GC17M005945 |
| ACTRT3 | GC03M169766 |
| ELOVL6 | GC04M110045 |
| TMPRSS15 | GC21M018269 |
| CAPN5 | GC11P077066 |
| ZNF512 | GC02P027582 |
| GPR15 | GC03P098531 |
| TTYH2 | GC17P074215 |
| TMEM41B | GC11M009280 |
| ENSG00000240731 | GC01M005133 |
| ZIC4 | GC03M147386 |
| PLCH1 | GC03M155381 |
| ENSG00000285106 | GC07M130793 |
| TOMM34 | GC20M044942 |
| ATP6V1C2 | GC02P010720 |
| CARD6 | GC05P040841 |
| TCEAL1 | GC0XP103628 |
| PAIP1P1 | GC06M030186 |
| SBF1 | GC22M055232 |
| DPH3 | GC03M016257 |
| CHRM4 | GC11M084307 |
| MT1F | GC16P056657 |
| RPP38 | GC10P015097 |
| PAFAH1B3 | GC19M042297 |
| MIR509-1 | GC0XM147260 |
| SETD9 | GC05P056909 |
| SLC17A8 | GC12P100357 |
| HEATR1 | GC01M236549 |
| LOXL3 | GC02M074532 |
| ZFP82 | GC19M061819 |
| TGFBRAP1 | GC02M105250 |
| CDS1 | GC04P084582 |
| ADH4 | GC04M099123 |
| MEAK7 | GC16M084476 |
| ATAD3C | GC01P001449 |
| UGT1A5 | GC02P233712 |
| CSDC2 | GC22P041560 |
| DDX60 | GC04M168216 |
| TMEM242 | GC06M157289 |
| HCG4B | GC06M061203 |
| HCG23 | GC06P076893 |
| MED4 | GC13M048053 |
| IL1F10 | GC02P113067 |
| MK280269-056 | GC06M062293 |
| LONP2 | GC16P048244 |
| PAOX | GC10P133379 |
| RWDD2A | GC06P083193 |
| SGMS2 | GC04P107824 |
| ZNF292 | GC06P087153 |
| MCHR1 | GC22P040679 |
| ZNF22 | GC10P045000 |
| HSBP1 | GC16P083719 |
| HS3ST2 | GC16P022814 |
| KRT73 | GC12M052607 |
| MIR450B | GC0XM134540 |
| BTN1A1 | GC06P026500 |
| POLR1E | GC09P039543 |
| RTTN | GC18M070003 |
| IER5 | GC01P181088 |
| ZNF165 | GC06P028080 |
| CLEC9A | GC12P010030 |
| GLYR1 | GC16M006498 |
| FAM83B | GC06P054846 |
| VPS72 | GC01M151176 |
| MDGA2 | GC14M046839 |
| ZSCAN4 | GC19P057651 |
| TFAP4 | GC16M006462 |
| GCFC2 | GC02M075652 |
| SLITRK6 | GC13M085792 |
| SBSN | GC19M063687 |
| SMG5 | GC01M156293 |
| IMP3 | GC15M075639 |
| PIP4P1 | GC14M021856 |
| SIGLEC15 | GC18P045825 |
| ZDHHC5 | GC11P057670 |
| SPC25 | GC02M168834 |
| BNC2-AS1 | GC09P016727 |
| NOMO3 | GC16P016232 |
| CMTM8 | GC03P032238 |
| MCMBP | GC10M119829 |
| MIR133A2 | GC20P063251 |
| ZFHX4 | GC08P076681 |
| MAGEE1 | GC0XP076427 |
| ADRA2C | GC04P003766 |
| PARP16 | GC15M079220 |
| ADH7 | GC04M099412 |
| ODAM | GC04P070195 |
| ENSG00000269737 | GC01P001671 |
| CFAP58 | GC10P104354 |
| ZNF777 | GC07M149431 |
| TMEM218 | GC11M125094 |
| ALPK1 | GC04P112285 |
| DMAC1 | GC09M007798 |
| TMEM14C | GC06P010723 |
| XIRP1 | GC03M039200 |
| TMEM33 | GC04P041937 |
| STX6 | GC01M180972 |
| PSMA3-AS1 | GC14M058140 |
| PCLO | GC07M082754 |
| CAMSAP1 | GC09M135808 |
| ATP2A1 | GC16P039272 |
| UNC13C | GC15P053838 |
| ZNF420 | GC19P037007 |
| ENSG00000271581 | GC06P076851 |
| BRINP2 | GC01P177170 |
| PKNOX2 | GC11P125164 |
| TNNI2 | GC11P001839 |
| TRIM46 | GC01P155173 |
| MIR541 | GC14P109094 |
| NDUFB7 | GC19M014566 |
| MON1A | GC03M050935 |
| MIR382 | GC14P109081 |
| TRPV5 | GC07M142908 |
| NUCB1 | GC19P048900 |
| MIR1266 | GC15M079993 |
| PEDS1 | GC20M050119 |
| NIPAL1 | GC04P047917 |
| EID1 | GC15P048877 |
| FAM117B | GC02P202999 |
| ARMC8 | GC03P138187 |
| ZNF311 | GC06M061150 |
| HSD17B7 | GC01P162790 |
| POLR2I | GC19M036113 |
| RGS8 | GC01M183190 |
| DIRAS1 | GC19M002714 |
| PRAF2 | GC0XM049143 |
| SLC16A6 | GC17M068267 |
| LINC01614 | GC02P215717 |
| CETN1 | GC18P000580 |
| H2BC15 | GC06P076686 |
| ZC3H15 | GC02P186486 |
| ENTPD6 | GC20P025196 |
| DPYS | GC08M104331 |
| HERC4 | GC10M067921 |
| CORO2A | GC09M098120 |
| MAGEH1 | GC0XP055452 |
| CCDC117 | GC22P028772 |
| SGSM2 | GC17P002345 |
| SERPINA2 | GC14M099729 |
| ARHGEF9 | GC0XM063634 |
| MIR448 | GC0XP114823 |
| LINC00662 | GC19M027663 |
| P3H4 | GC17M041801 |
| PFKFB1 | GC0XM054932 |
| DHRS4 | GC14P023953 |
| TAS2R4 | GC07P147870 |
| SLC9A3-AS1 | GC05P000474 |
| GABRA6 | GC05P161547 |
| DBI | GC02P119366 |
| DNTTIP1 | GC20P045791 |
| SLC35D2 | GC09M096313 |
| RF00017-5028 | GC06M063079 |
| ZBTB1 | GC14P064503 |
| SLC46A2 | GC09M112878 |
| ZWINT | GC10M056357 |
| BTN2A3P | GC06P076626 |
| MIR4513 | GC15M074788 |
| INPP5J | GC22P033752 |
| MIR613 | GC12P018794 |
| GRAP | GC17M024693 |
| TRNAU1AP | GC01P028553 |
| IL1R1-AS1 | GC02M102174 |
| ARMCX1 | GC0XP101550 |
| TNNT3 | GC11P001920 |
| TYSND1 | GC10M070137 |
| PLPP2 | GC19M004269 |
| TSSK1B | GC05M113432 |
| ETF1P1 | GC06P076824 |
| ATG2B | GC14M099759 |
| SCFD1 | GC14P030622 |
| INHBA-AS1 | GC07P041693 |
| PTGR2 | GC14P073870 |
| KRT86 | GC12P052249 |
| LEMD1 | GC01M205381 |
| PALD1 | GC10P070800 |
| CHRNB3 | GC08P042697 |
| ANO9 | GC11M002642 |
| MIR379 | GC14P109077 |
| ATRN | GC20P003471 |
| DHX35 | GC20P038963 |
| GSTM4 | GC01P109657 |
| TMEM45A | GC03P100492 |
| CSRNP1 | GC03M039159 |
| HCG27 | GC06P031197 |
| FBXL20 | GC17M039252 |
| CNPY2 | GC12M056309 |
| ZNF331 | GC19P053546 |
| NACC2 | GC09M136006 |
| PGBD1 | GC06P028281 |
| TKTL2 | GC04M163471 |
| H2AW | GC01M228539 |
| ANXA10 | GC04P168081 |
| RPP30 | GC10P090871 |
| PLD5 | GC01M242082 |
| SYT13 | GC11M045240 |
| GALNT13 | GC02P153871 |
| GRIN3A | GC09M101569 |
| MRPS31 | GC13M040729 |
| RPP40 | GC06M004994 |
| MRFAP1 | GC04P006640 |
| LYSMD1 | GC01M151481 |
| RNU6-850P | GC06M031756 |
| ZNF282 | GC07P149195 |
| IGES | GC05U990033 |
| NOXO1 | GC16M006278 |
| ZNF207 | GC17P049882 |
| SHROOM1 | GC05M132822 |
| NOL10 | GC02M010662 |
| CHID1 | GC11M000867 |
| BNIP2 | GC15M059659 |
| GXYLT1 | GC12M042081 |
| TPH2 | GC12P071938 |
| RILP | GC17M001646 |
| CNTNAP3 | GC09M039064 |
| ECPAS | GC09M111361 |
| ATG9A | GC02M219219 |
| DDX43 | GC06P073394 |
| GSTA3 | GC06M052896 |
| MAP3K10 | GC19P040191 |
| MIR543 | GC14P108796 |
| NALF1 | GC13M107164 |
| ZNF484 | GC09M093131 |
| LIPN | GC10P090720 |
| ARL1 | GC12M101393 |
| GPX6 | GC06M028503 |
| TEX19 | GC17P082359 |
| CHRNA6 | GC08M042752 |
| ITLN2 | GC01M160945 |
| CYP2W1 | GC07P000983 |
| ATP13A1 | GC19M019645 |
| NBPF14 | GC01M148531 |
| MAGEB1 | GC0XP030244 |
| ZG16B | GC16P009921 |
| MRPS12 | GC19P038930 |
| RNF128 | GC0XP106693 |
| USP17L3 | GC08M007976 |
| CDKL1 | GC14M050330 |
| FAM3B | GC21P041304 |
| MIR1301 | GC02M025328 |
| ZNF362 | GC01P033256 |
| IZUMO1R | GC11P094305 |
| UBA3 | GC03M069054 |
| ZKSCAN8P1 | GC06P079851 |
| SREK1 | GC05P066139 |
| ZNF132 | GC19M058432 |
| EIF4E3 | GC03M071675 |
| RFNG | GC17M082047 |
| CEBPG | GC19P033373 |
| lnc-HNF1B-3 | GC17M037293 |
| HOXD11 | GC02P176104 |
| H2AC14 | GC06M061062 |
| CMTM5 | GC14P030867 |
| OR9Q1 | GC11P058023 |
| CORO2B | GC15P092248 |
| PRINS | GC10U900902 |
| ATP5F1C | GC10P007789 |
| RF00017-1167 | GC12M111251 |
| TOB2 | GC22M041433 |
| AFM | GC04P073481 |
| YKT6 | GC07P044200 |
| GPR39 | GC02P136091 |
| SLC35E1 | GC19M016549 |
| CKMT1B | GC15P043593 |
| HCG25 | GC06P076925 |
| ZNF124 | GC01M247121 |
| LINC00189 | GC21P029193 |
| TMC3 | GC15M081331 |
| XLOC_008559 | GC10P090947 |
| MYBPH | GC01M203136 |
| KTN1-AS1 | GC14M055525 |
| MIR194-2 | GC11M084636 |
| LCORL | GC04M017844 |
| DPH6 | GC15M035217 |
| DNAJC10 | GC02P182716 |
| LOC105379013 | GC05M068429 |
| NCK1-DT | GC03M136842 |
| CACNB1 | GC17M039173 |
| TAS1R3 | GC01P001331 |
| DMRT3 | GC09P000976 |
| NYAP2 | GC02P225400 |
| POTEF-AS1 | GC02P130109 |
| PART1 | GC05P060517 |
| LINC02820 | GC12P085320 |
| ZNF254 | GC19P051178 |
| MRPL21 | GC11M084852 |
| LMAN2 | GC05M177856 |
| SYT9 | GC11P007238 |
| SLC22A17 | GC14M023346 |
| MFSD2B | GC02P024010 |
| ENSG00000272540 | GC06M061232 |
| COLGALT2 | GC01M183899 |
| SPANXA2 | GC0XP141590 |
| WARS2-IT1 | GC01P119047 |
| CSMD2 | GC01M033513 |
| BET1L | GC11M002627 |
| HCAR2 | GC12M122701 |
| USP35 | GC11P078188 |
| IPCEF1 | GC06M154154 |
| SHISA3 | GC04P042399 |
| CATSPERB | GC14M091580 |
| CECR2 | GC22P017359 |
| LINC00328 | GC21U900445 |
| FBXO30 | GC06M145795 |
| SYT12 | GC11P069257 |
| PCMTD1 | GC08M051817 |
| MIR520B | GC19P062499 |
| SH3GLB2 | GC09M129007 |
| TRIM3 | GC11M006450 |
| CA3 | GC08P085373 |
| CHPF | GC02M219538 |
| KRT74 | GC12M052565 |
| CMPK1 | GC01P047333 |
| MIR486-2 | GC08P041701 |
| NETO2 | GC16M047077 |
| SOCS7 | GC17P050262 |
| MCCD1 | GC06P031528 |
| C6orf52 | GC06M010671 |
| IDO2 | GC08P039949 |
| H2BC12 | GC06M060993 |
| NIT2 | GC03P100334 |
| SLC25A45 | GC11M065375 |
| LINC00886 | GC03M156747 |
| DGKI | GC07M137381 |
| RAPGEFL1 | GC17P040177 |
| EPS8L2 | GC11P000694 |
| ZNF600 | GC19M052749 |
| REPIN1 | GC07P150368 |
| RHOBTB1 | GC10M060869 |
| OST4 | GC02M027070 |
| RALGPS1 | GC09P126914 |
| GABRA4 | GC04M046836 |
| GOLT1B | GC12P021501 |
| DEFA3 | GC08M007015 |
| MIR153-2 | GC07M157574 |
| CYP4F22 | GC19P015508 |
| TPGS2 | GC18M036777 |
| TNFAIP1 | GC17P028335 |
| NRSN1 | GC06P024126 |
| IGDCC4 | GC15M065381 |
| TMEM40 | GC03M012733 |
| LINC01315 | GC22M055105 |
| ZNF547 | GC19P062663 |
| TMC4 | GC19M054160 |
| MFSD14B | GC09P094454 |
| ACAD10 | GC12P111686 |
| H2BC1 | GC06P076606 |
| KCNJ9 | GC01P160079 |
| SEC14L4 | GC22M030488 |
| GH2 | GC17M063880 |
| TSEN34 | GC19P063074 |
| KIAA0040 | GC01M175126 |
| SEC22C | GC03M042547 |
| FREM3 | GC04M143577 |
| ENSG00000272501 | GC06M061256 |
| ZNF18 | GC17M011957 |
| TMEM207 | GC03M190428 |
| SNX3 | GC06M108211 |
| PXMP4 | GC20M033776 |
| HHLA1 | GC08M132061 |
| MLXIP | GC12P122078 |
| MIR1236 | GC06M061324 |
| PLEKHO2 | GC15P114558 |
| NCOA7 | GC06P125781 |
| HNRNPUL2 | GC11M062712 |
| ALX3 | GC01M110059 |
| LINC00974 | GC17M041549 |
| SSBP2 | GC05M081413 |
| LRRN2 | GC01M204586 |
| HSALNG0070392 | GC09M021932 |
| CPT1B | GC22M055236 |
| ATMIN | GC16P081035 |
| HSF4 | GC16P067164 |
| CFAP45 | GC01M159873 |
| HEBP1 | GC12M012974 |
| lnc-ZFP36L1-9 | GC14M068397 |
| INTS10 | GC08P019817 |
| ZNF70 | GC22M035073 |
| MSH5-SAPCD1 | GC06P076880 |
| MIR147A | GC09M120244 |
| GIMAP7 | GC07P150514 |
| NUTF2 | GC16P067846 |
| SPINDOC | GC11P063814 |
| PACSIN1 | GC06P076974 |
| FAM114A2 | GC05M153990 |
| ASXL3 | GC18P033578 |
| COMMD7 | GC20M032702 |
| RHBDL3 | GC17P049879 |
| EVX1 | GC07P027627 |
| H3-5 | GC12M031865 |
| LINC01355 | GC01M023281 |
| SEPHS2 | GC16M035849 |
| B3GNT3 | GC19P017794 |
| MIR325 | GC0XM077005 |
| H2AC7 | GC06M061826 |
| MTFP1 | GC22P033713 |
| PCDHB11 | GC05P145524 |
| TRUB1 | GC10P114938 |
| OAS2 | GC12P112978 |
| C5orf15 | GC05M133955 |
| PRXL2A | GC10P091407 |
| POLR3K | GC16M000046 |
| ZBTB14 | GC18M005289 |
| TMED8 | GC14M077335 |
| LINC00240 | GC06P026956 |
| EIF4EBP3 | GC05P145499 |
| GIHCG | GC12M058291 |
| ZRANB2 | GC01M071063 |
| MED10 | GC05M006371 |
| PCTP | GC17P055750 |
| FBXL8 | GC16P067160 |
| PCDHGA5 | GC05P145541 |
| MIR758 | GC14P109101 |
| IGIP | GC05P140125 |
| POLR2K | GC08P100150 |
| MIR630 | GC15P072587 |
| LINC01419 | GC08P083403 |
| NDUFAB1 | GC16M023582 |
| RTCA | GC01P100266 |
| ART1 | GC11P003642 |
| H2AC1 | GC06M025947 |
| CLUH | GC17M002689 |
| EML2 | GC19M045606 |
| MIR2355 | GC02M207109 |
| SERPINB10 | GC18P063897 |
| DEFB103A | GC08P007881 |
| TMC5 | GC16P019422 |
| PARP12 | GC07M140023 |
| NAA35 | GC09P085941 |
| CLDN17 | GC21M030165 |
| HOXD9 | GC02P176122 |
| NKX2-1-AS1 | GC14P036519 |
| MSRB2 | GC10P023095 |
| SYNC | GC01M032679 |
| ZNF76 | GC06P076989 |
| DENND5A | GC11M009162 |
| MIR488 | GC01M177029 |
| ABCF3 | GC03P184186 |
| RGL4 | GC22P023688 |
| WDHD1 | GC14M054938 |
| MIR802 | GC21P035720 |
| CHCHD4 | GC03M019708 |
| CMBL | GC05M010275 |
| PLEKHB1 | GC11P073647 |
| PPP4R4 | GC14P094146 |
| C6orf136 | GC06P076839 |
| LINC01525 | GC01P117272 |
| CCZ1B | GC07M006794 |
| MIR3619 | GC22P046091 |
| ODR4 | GC01P186376 |
| NT5C1B | GC02M018562 |
| RABGAP1 | GC09P122932 |
| LINC00393 | GC13M073413 |
| THOC3 | GC05M175917 |
| ZFP3 | GC17P005078 |
| HEXIM2 | GC17P045159 |
| BARX1 | GC09M093951 |
| DDA1 | GC19P050914 |
| LELP1 | GC01P153175 |
| AMBN | GC04P070593 |
| SOX14 | GC03P137764 |
| RNF187 | GC01P228487 |
| SPSB3 | GC16M001776 |
| RGS13 | GC01P192636 |
| DERA | GC12P015911 |
| REXO2 | GC11P114439 |
| ZC3H12A-DT | GC01M037354 |
| ODAPH | GC04P075555 |
| ANXA9 | GC01P150982 |
| IGHA1 | GC14M112098 |
| XLOC_009911 | GC12P125494 |
| RIDA | GC08M098103 |
| ARHGEF10L | GC01P017657 |
| H2AC8 | GC06P077463 |
| TRUB2 | GC09M128430 |
| SH3BP5L | GC01M248810 |
| TRIM58 | GC01P247857 |
| H2BC4 | GC06M061828 |
| SPRR2D | GC01M153039 |
| LOC111365141 | GC17P027800 |
| ENSG00000230092 | GC01M000800 |
| VIM2P | GC06M126602 |
| SLC2A1-DT | GC01P043010 |
| ETFBKMT | GC12P031649 |
| LINC00324 | GC17M010126 |
| ADAM7 | GC08P024440 |
| GSTT2B | GC22M023957 |
| PCDHB14 | GC05P145531 |
| MIR300 | GC14P109065 |
| MIR877 | GC06P030584 |
| IGFBPL1 | GC09M039734 |
| MIR509-3 | GC0XM147259 |
| TAAR1 | GC06M132644 |
| H2AJ | GC12P018856 |
| RALGAPA2 | GC20M020374 |
| SNX15 | GC11P065031 |
| LINC02055 | GC08P135859 |
| XPOT | GC12P064404 |
| MIR503HG | GC0XM134683 |
| PADI6 | GC01P017652 |
| SNORD117 | GC06M061298 |
| TFAP2A-AS2 | GC06P010404 |
| GSTM5 | GC01P109711 |
| GLP2R | GC17P009822 |
| TM2D2 | GC08M038988 |
| LUZP2 | GC11P024518 |
| TSGA10IP | GC11P069028 |
| MAP1A | GC15P043516 |
| HSD17B6 | GC12P056752 |
| GPR150 | GC05P095620 |
| CKMT2 | GC05P081233 |
| GABRR2 | GC06M089257 |
| ZNF473 | GC19P052372 |
| IPO11 | GC05P062403 |
| PRSS22 | GC16M002852 |
| BLOC1S4 | GC04P006718 |
| BCL2L15 | GC01M113876 |
| CYP4F2 | GC19M015878 |
| ENSG00000232807 | GC10M005935 |
| PHF21B | GC22M044881 |
| TAFA4 | GC03M068714 |
| MAGEB5 | GC0XP026297 |
| TMEM54 | GC01M032894 |
| PCDHB16 | GC05P145523 |
| C5orf24 | GC05P134845 |
| ENSG00000283321 | GC07P017299 |
| HCAR3 | GC12M122714 |
| HEPHL1 | GC11P094021 |
| PRPSAP2 | GC17P042711 |
| DUSP14 | GC17P050113 |
| NOL7 | GC06P013615 |
| RNF38 | GC09M036336 |
| TMEM92 | GC17P051913 |
| MAP6 | GC11M075586 |
| DSEL | GC18M067506 |
| OSER1 | GC20M044195 |
| RFESD | GC05P095646 |
| ZNF510 | GC09M096755 |
| OTP | GC05M077628 |
| MAGEA9B | GC0XM149582 |
| XLOC_007697 | GC09P046270 |
| PWWP3B | GC0XP106169 |
| CAVIN4 | GC09P100576 |
| RNF123 | GC03P049689 |
| SOWAHC | GC02P109614 |
| LINC02866 | GC08P040129 |
| CDK5R2 | GC02P218959 |
| CBLN4 | GC20M055997 |
| UBL4A | GC0XM154483 |
| STARD13-AS | GC13P033180 |
| KCTD14 | GC11M085126 |
| OBI1 | GC13M078615 |
| DNHD1 | GC11P006497 |
| PCDHGB7 | GC05P145553 |
| PURG | GC08M030995 |
| IMPA2 | GC18P011981 |
| IFNK | GC09P027514 |
| MIR577 | GC04P114656 |
| SLC6A5 | GC11P020599 |
| MT1M | GC16P056632 |
| ADAMTS16 | GC05P005140 |
| KRT33B | GC17M041363 |
| LINC02569 | GC06P077978 |
| MN298114-186 | GC06P077180 |
| CPN1 | GC10M100042 |
| ADH6 | GC04M099202 |
| ECH1 | GC19M038815 |
| MIR655 | GC14P109097 |
| RNU6-1 | GC15M067839 |
| ZFAND4 | GC10M046253 |
| PCNPP1 | GC12M111666 |
| BAGE2 | GC21P010413 |
| KPRP | GC01P152766 |
| RBP2 | GC03M139452 |
| MIR1275 | GC06M061425 |
| LOC110283621 | GC04P087973 |
| PAQR7 | GC01M025861 |
| SLC37A3 | GC07M140293 |
| LOC101927533 | GC02P065436 |
| DUSP9 | GC0XP153642 |
| MYL10 | GC07M101613 |
| RF00017-6826 | GC08P102447 |
| MIR504 | GC0XM138667 |
| FKBP11 | GC12M049346 |
| PTMS | GC12P006765 |
| MACROD2 | GC20P013925 |
| TNNC2 | GC20M045823 |
| KCNH6 | GC17P063523 |
| TIMM21 | GC18P074148 |
| SNHG32 | GC06P079825 |
| PTCSC1 | GC08P133054 |
| MRPL20-DT | GC01P003539 |
| ENSG00000284299 | GC01M210858 |
| MIR601 | GC09M123402 |
| TNNI1 | GC01M201404 |
| ALS2CL | GC03M046685 |
| MTURN | GC07P030134 |
| MIR384 | GC0XM076919 |
| KDM4D | GC11P094973 |
| MIR769 | GC19P046018 |
| STAP2 | GC19M004324 |
| COMMD6 | GC13M075525 |
| TRIM39-RPP21 | GC06P076833 |
| KRT19P3 | GC04M109879 |
| MIR631 | GC15M075353 |
| N4BP3 | GC05P178113 |
| MIR34AHG | GC01M009149 |
| NR2F2-AS1 | GC15M114783 |
| NMNAT3 | GC03M139560 |
| PADI1 | GC01P017205 |
| ACP7 | GC19P051555 |
| KRT82 | GC12M052393 |
| SYT8 | GC11P001829 |
| PAUPAR | GC11P031820 |
| FBXO25 | GC08P000406 |
| ILDR1 | GC03M121987 |
| HSALNG0094037 | GC12P111432 |
| RF00017-5031 | GC06P079677 |
| ABITRAM | GC09P108935 |
| TSBP1-AS1 | GC06P079847 |
| ZNF84 | GC12P133037 |
| BCO1 | GC16P081238 |
| C11orf1 | GC11P111878 |
| ATPSCKMT | GC05M010225 |
| PAIP2 | GC05P139353 |
| EFHD1 | GC02P232606 |
| LRP11 | GC06M149818 |
| FAM135A | GC06P070412 |
| ECHDC2 | GC01M052895 |
| ENSG00000251409 | GC05M095835 |
| SRPK3 | GC0XP153776 |
| FAM120C | GC0XM054069 |
| SAMMSON | GC03P069999 |
| KRTDAP | GC19M062881 |
| SNORD46 | GC01P045239 |
| ENSG00000224934 | GC10P099443 |
| LINC01128 | GC01P003351 |
| MIR942 | GC01P117094 |
| C1QTNF7 | GC04P015339 |
| DCUN1D4 | GC04P051833 |
| CRIP1 | GC14P105486 |
| DCAF10 | GC09P037800 |
| TMPRSS11E | GC04P068447 |
| RPSAP52 | GC12M065758 |
| ENSG00000271949 | GC01P089633 |
| METTL2B | GC07P131411 |
| SPATA33 | GC16P091050 |
| COA5 | GC02M098599 |
| KRT37 | GC17M041422 |
| DQ485454 | GC09P022029 |
| TEX29 | GC13P111306 |
| PGLYRP2 | GC19M015468 |
| UNC5CL | GC06M061485 |
| RSPO4 | GC20M000958 |
| FSD1L | GC09P105447 |
| ZBTB5 | GC09M037431 |
| BAGE3 | GC21U900493 |
| ZNF667 | GC19M062562 |
| SEC14L3 | GC22M030447 |
| ARMH3 | GC10M101846 |
| LYPD5 | GC19M061982 |
| VSIG10L | GC19M062319 |
| GBP6 | GC01P089363 |
| KRT36 | GC17M041486 |
| ENSG00000284895 | GC01M041585 |
| TMEM263 | GC12P106955 |
| UAP1L1 | GC09P137077 |
| FAM87B | GC01P000817 |
| MB21D2 | GC03M192796 |
| FAM220A | GC07M006353 |
| SHC2 | GC19M004279 |
| PMCH | GC12M102196 |
| MAGEC3 | GC0XP141838 |
| HDGFL1 | GC06P022569 |
| BAGE5 | GC13Pr00076 |
| lnc-FAM109A-1 | GC12M111525 |
| LIF-AS1 | GC22P033710 |
| SLC25A23 | GC19M006436 |
| ZNF454 | GC05P178941 |
| H2BC9 | GC06P077471 |
| TMEM74 | GC08M108606 |
| PRTFDC1 | GC10M024848 |
| SMIM38 | GC11P069490 |
| TFAP2A-AS1 | GC06P010557 |
| ZNF772 | GC19M057466 |
| MIR1297 | GC13M054311 |
| NKAPL | GC06P028259 |
| NPFFR1 | GC10M070247 |
| MIR935 | GC19P062512 |
| H2AC11 | GC06P077458 |
| NRK | GC0XP105822 |
| CTAGE9 | GC06M131708 |
| LY6E-DT | GC08M142982 |
| GPR12 | GC13M026755 |
| NACA2 | GC17M061590 |
| MIR4713HG | GC15P051039 |
| ZNF594 | GC17M005994 |
| CPNE5 | GC06M061448 |
| ACOT9 | GC0XM023701 |
| ARHGDIG | GC16P009817 |
| TMED3 | GC15P079311 |
| APCDD1L-DT | GC20P058516 |
| MIR652 | GC0XP110055 |
| ILVBL | GC19M015116 |
| C14orf39 | GC14M060396 |
| SLC35D3 | GC06P136922 |
| REG1B | GC02M079086 |
| B4GALNT4 | GC11P000369 |
| RF00017-4261 | GC05P056563 |
| COL22A1 | GC08M138588 |
| SHISA5 | GC03M048468 |
| HMX1 | GC04M008847 |
| ZBED2 | GC03M111592 |
| EBAG9P1 | GC10M099697 |
| PPIAP9 | GC06M031518 |
| GSX2 | GC04P054099 |
| TAS2R16 | GC07M122994 |
| MT4 | GC16P056565 |
| TMEM128 | GC04M004264 |
| TMPO-AS1 | GC12M098512 |
| AMY1A | GC01P103651 |
| WSCD2 | GC12P108129 |
| ZBBX | GC03M167240 |
| ZIM3 | GC19M057134 |
| DYRK4 | GC12P018508 |
| SVOPL | GC07M138594 |
| SNAP91 | GC06M083553 |
| MIR1303 | GC05P154685 |
| ZNF239 | GC10M043594 |
| STK32B | GC04P005053 |
| LINC01638 | GC22M035109 |
| PLA2G4B | GC15P041837 |
| ENSG00000258017 | GC12P049110 |
| LSINCT5 | GC05P002712 |
| PITPNA-AS1 | GC17P001516 |
| GABRG1 | GC04M046035 |
| GPR32 | GC19P050770 |
| MIR329-1 | GC14P109068 |
| RNASE7 | GC14P021042 |
| MIR766 | GC0XM119646 |
| FRY-AS1 | GC13M036042 |
| ZNF483 | GC09P111525 |
| piR-48007 | GC12M111529 |
| piR-36455 | GC12M111444 |
| piR-38259 | GC12M111524 |
| piR-50346 | GC12M111530 |
| piR-51327 | GC12M111437 |
| piR-51449 | GC12P111448 |
| HSALNG0094038 | GC12P111446 |
| piR-56480-015 | GC12M111439 |
| C5orf67 | GC05M056511 |
| MMGT1 | GC0XM135962 |
| BAGE4 | GC21U900419 |
| ZNF81 | GC0XP047836 |
| NAT8L | GC04P002061 |
| VCX | GC0XP007842 |
| MIR1224 | GC03P184241 |
| LINC01786 | GC01P003365 |
| RTF2 | GC20P056469 |
| MYOSLID | GC02P207225 |
| HSALNG0026758 | GC03P071461 |
| RGS18 | GC01P192158 |
| RNA5SP203 | GC06M010766 |
| C3orf33 | GC03M155764 |
| FAM201A | GC09P038620 |
| PTPN20 | GC10P046911 |
| HSALNG0049429 | GC06P077654 |
| ZNF182 | GC0XM047974 |
| AUNIP | GC01M025862 |
| ZNF195 | GC11M003336 |
| MIR1258 | GC02M179860 |
| PRR4 | GC12M019803 |
| RPL15P4 | GC06P076857 |
| ZDHHC19 | GC03M196197 |
| BAALC-AS1 | GC08M103156 |
| GXYLT2 | GC03P072888 |
| TCHHL1 | GC01M152085 |
| CRLS1 | GC20P006005 |
| LGSN | GC06M063275 |
| HACD4 | GC09M020999 |
| LRRC40 | GC01M070144 |
| DMRTA2 | GC01M050417 |
| TSBP1 | GC06M032288 |
| RABGGTB | GC01P075786 |
| DGKK | GC0XM050365 |
| LRRC66 | GC04M051993 |
| NME1-NME2 | GC17P051153 |
| TREX2 | GC0XM153444 |
| LOC108281177 | GC03P181707 |
| ENSG00000234389 | GC02P102438 |
| HSD17B14 | GC19M048813 |
| SLC38A7 | GC16M058665 |
| JMJD7 | GC15P041829 |
| IFNA17 | GC09M021227 |
| WTAPP1 | GC11P102746 |
| LGALS12 | GC11P063506 |
| ZNF396 | GC18M035366 |
| UGT2B4 | GC04M069484 |
| INHBC | GC12P057434 |
| UBLCP1 | GC05P159263 |
| ANXA2R-AS1 | GC05P044178 |
| ACAP3 | GC01M001292 |
| MIR18B | GC0XM134218 |
| MAGEB3 | GC0XP030230 |
| SIRPB2 | GC20M001470 |
| MIR579 | GC05M032431 |
| RNF103 | GC02M086603 |
| ASCL4 | GC12P107774 |
| MIR889 | GC14P109103 |
| MAGEE2 | GC0XM075782 |
| ENSG00000254987 | GC11M103656 |
| ZSCAN5A | GC19M062549 |
| OTOR | GC20P016748 |
| MFSD6L | GC17M008797 |
| WASH8P | GC12M000015 |
| CRISP2 | GC06M061626 |
| LEXM | GC01P054807 |
| ZFAND2B | GC02P219195 |
| FUT11 | GC10P073772 |
| PPP1R14B | GC11M064244 |
| ST7-AS1 | GC07M116943 |
| CZIB | GC01M053215 |
| LINC01123 | GC02P109986 |
| DHRS1 | GC14M024290 |
| L3MBTL4 | GC18M005954 |
| CA11 | GC19M062154 |
| MIR596 | GC08P001817 |
| H2AC10P | GC06M061824 |
| MIR147B | GC15P045433 |
| ENSG00000250240 | GC05M095701 |
| HSALNG0129683 | GC20P033999 |
| MIR219A2 | GC09M128440 |
| LINC01556 | GC06P028943 |
| OBP2B | GC09M133205 |
| DQX1 | GC02M074518 |
| ENSG00000248373 | GC04P104653 |
| SERPINB11 | GC18P063647 |
| lnc-GCM1-4 | GC06M053275 |
| TUSC2P1 | GC0YP005887 |
| MIR646 | GC20P060308 |
| SNORA67 | GC17P010612 |
| PRSS27 | GC16M006359 |
| COX7A1 | GC19M062678 |
| TMEM200C | GC18M005883 |
| ARMC7 | GC17P075109 |
| ENSG00000255197 | GC11M085202 |
| MIR765 | GC01M156905 |
| NRIP3 | GC11M009001 |
| LOC110806262 | GC17P030235 |
| TTTY15 | GC0YP012538 |
| NONHSAG045678.2-002 | GC17M037673 |
| B3GAT2 | GC06M070856 |
| RCC1L | GC07M075044 |
| ENSG00000258101 | GC12M049232 |
| GLB1L | GC02M219237 |
| PHACTR2-AS1 | GC06M143555 |
| TOM1L1 | GC17P054901 |
| piR-57176-462 | GC03M024280 |
| MIR664A | GC01M220200 |
| NRBF2P5 | GC10P005775 |
| GASAL1 | GC08P102807 |
| RNF181 | GC02P085593 |
| LOC102724334 | GC21M005972 |
| RNASE4 | GC14P030634 |
| MIR576 | GC04P109488 |
| PPIP5K1 | GC15M043533 |
| MYLPF | GC16P030370 |
| FAM99B | GC11M002716 |
| ZKSCAN7 | GC03P044556 |
| lnc-ATE1-6 | GC10M121564 |
| MIR1238 | GC19P010552 |
| CHRFAM7A | GC15M030360 |
| SMIM13 | GC06P011288 |
| PELATON | GC20P050248 |
| CAPN14 | GC02M031173 |
| PRSS56 | GC02P232520 |
| MIR4443 | GC03P048654 |
| RPS10P7 | GC01P201518 |
| SCML2 | GC0XM018167 |
| PLAAT1 | GC03P193244 |
| ENSG00000242198 | GC05P075375 |
| SREBF2-AS1 | GC22M056090 |
| TSSK2 | GC22P033244 |
| MIR1229 | GC05M179798 |
| SKOR2 | GC18M047206 |
| MIR3120 | GC01P172138 |
| MIR634 | GC17P066787 |
| LINC02577 | GC07P106777 |
| SLC22A13 | GC03P038265 |
| LOC100507053 | GC04P099088 |
| ENSG00000236308 | GC10P100190 |
| ENSG00000272040 | GC05P075608 |
| POGK | GC01P166809 |
| PCSK4 | GC19M001481 |
| ZSCAN16-AS1 | GC06M061071 |
| PAXX | GC09P137046 |
| HSALNG0086841 | GC11P103637 |
| HOXC13-AS | GC12M053935 |
| CCDC168 | GC13M102729 |
| MIR487A | GC14P109087 |
| PLPPR2 | GC19P011445 |
| LOC106728418 | GC07P128238 |
| CARMAL | GC15P089040 |
| ELOF1 | GC19M011551 |
| WWTR1-AS1 | GC03P149657 |
| KRTAP5-6 | GC11P001718 |
| LINC02571 | GC06M061266 |
| ENSG00000249016 | GC05M054956 |
| EMC6 | GC17P003668 |
| RF00017-749 | GC10P121558 |
| ZNF542P | GC19P056368 |
| HES3 | GC01P006244 |
| HMX2 | GC10P123142 |
| SLC22A14 | GC03P038281 |
| OTUD1 | GC10P023439 |
| RNU6-351P | GC04P104974 |
| ZAN | GC07P100733 |
| GEMIN8P4 | GC01M089993 |
| ENSG00000205653 | GC06P000203 |
| THAP9 | GC04P082900 |
| MIR450A1 | GC0XM134633 |
| MORN5 | GC09P122159 |
| SLC16A14 | GC02M230034 |
| TMPRSS11B | GC04M068226 |
| MIR4319 | GC18M044970 |
| LINC01882 | GC18M023468 |
| MIR519A1 | GC19P053752 |
| NRIR | GC02M006828 |
| UGT2B10 | GC04P068816 |
| SMIM10L2A | GC0XP135421 |
| ENSG00000249856 | GC05M074917 |
| MTCO3P1 | GC06M032706 |
| SDHAF3 | GC07P097116 |
| TMEM182 | GC02P102744 |
| MIR1281 | GC22P041092 |
| lnc-NR1D2-4 | GC03P024431 |
| MIR639 | GC19P014529 |
| HSALNG0049245-002 | GC06M061945 |
| HSALNG0102089 | GC14M068557 |
| TXNDC17 | GC17P006640 |
| MIR3662 | GC06M134979 |
| STYXL2 | GC01P167095 |
| LOC107372315 | GC14P030590 |
| ATXN2-AS | GC12P111600 |
| TMEM229A | GC07M124030 |
| MAGEB16 | GC0XP035816 |
| MIR496 | GC14P109092 |
| RNFT2 | GC12P116738 |
| C9orf57 | GC09M072051 |
| RASSF7 | GC11P000560 |
| ENSG00000285991 | GC06M149817 |
| ENSG00000274017 | GC12P049196 |
| MIR597 | GC08P009741 |
| OBI1-AS1 | GC13P077920 |
| KRT28 | GC17M040792 |
| CFAP100 | GC03P126395 |
| ENSG00000240535 | GC05M055022 |
| GPRIN2 | GC10M046543 |
| LINC00290 | GC04M181064 |
| GALNTL6 | GC04P171813 |
| LOC108961161 | GC06P078234 |
| RNU6ATAC | GC09M134164 |
| LNCAROD | GC10M052455 |
| TAS2R41 | GC07P143477 |
| OR2J3 | GC06P076788 |
| WDFY3-AS2 | GC04P084965 |
| KRT33A | GC17M041346 |
| piR-48820-008 | GC01M155215 |
| ENSG00000249379 | GC06P053427 |
| RNU6-1224P | GC08M102533 |
| PLA2G4D | GC15M042067 |
| SNORD12B | GC20P049345 |
| CYP2A7 | GC19M040875 |
| MIR3194 | GC20M051452 |
| FAM238C | GC10M026932 |
| ZNF658 | GC09P066856 |
| LINC01605 | GC08M037407 |
| POM121L2 | GC06M027285 |
| RGPD3 | GC02M106373 |
| ACTL7A | GC09P108862 |
| LINC01997 | GC03P168953 |
| UBE2D4 | GC07P043926 |
| ZNF189 | GC09P101398 |
| MIR466 | GC03M031161 |
| ENSG00000251259 | GC04M105137 |
| MAGEB18 | GC0XP026138 |
| ENSG00000248993 | GC06M032937 |
| RNF180 | GC05P064165 |
| LOC111255645 | GC08P023229 |
| MIR604 | GC10M029545 |
| AMY2A | GC01P103616 |
| ZNF695 | GC01M246945 |
| MIR365B | GC17P049835 |
| MSANTD3-TMEFF1 | GC09P100441 |
| RBBP8NL | GC20M062410 |
| lnc-ZAR1L-2 | GC13M036083 |
| MIR1256 | GC01M020988 |
| MIR4293 | GC10M014425 |
| ENSG00000226647 | GC10M005712 |
| MIR4295 | GC10P112634 |
| LINC00551 | GC13P106575 |
| TMEM221 | GC19M017435 |
| C5orf22 | GC05P031532 |
| ZMAT5 | GC22M029730 |
| ENSG00000266446 | GC09P021995 |
| PANX3 | GC11P124611 |
| HSALNG0049427 | GC06P077653 |
| ZNF583 | GC19P056397 |
| MIR4260 | GC01M209623 |
| SNORD80 | GC01M174483 |
| CYP4X1 | GC01P046961 |
| HSALNG0007483 | GC01M155226 |
| KRT39 | GC17M040958 |
| LOC108254682 | GC09P037034 |
| RNU5A-1 | GC15P065296 |
| RALY-AS1 | GC20M033983 |
| PPIAL4E | GC01M144372 |
| MIR550A3 | GC07M030409 |
| TAS2R45 | GC12Mj00291 |
| IPO8P1 | GC01M210859 |
| piR-34974-004 | GC11M103837 |
| lnc-RD3-9 | GC01M210720 |
| FETUB | GC03P186635 |
| HNRNPA3P1 | GC10M043787 |
| NR4A1AS | GC12M052059 |
| MIR3127 | GC02P096798 |
| piR-30853-017 | GC11M103785 |
| MIR564 | GC03P046247 |
| C6orf141 | GC06P077120 |
| ENSG00000251473 | GC04M105103 |
| ATP13A5 | GC03M193274 |
| MIR552 | GC01M034669 |
| MIR4693 | GC11P103849 |
| ENSG00000228650 | GC05P056771 |
| ABALON | GC20P031721 |
| SNORD74 | GC01M174484 |
| FMR1-AS1 | GC0XM147910 |
| MICB-DT | GC06M061279 |
| HSALNG0067436 | GC08M102499 |
| MIR1285-1 | GC07M092204 |
| THRIL | GC12M125025 |
| HSALNG0049428 | GC06M061968 |
| lnc-HLA-DRB1-8 | GC06M032619 |
| RF00017-5040 | GC06M063086 |
| HPS1-AS1 | GC10P098462 |
| MIR588 | GC06P126484 |
| lnc-MFSD9-11 | GC02M102239 |
| PRSS57 | GC19M004315 |
| SBK1 | GC16P028260 |
| FAM225A | GC09P113112 |
| piR-41406-042 | GC20P034010 |
| HSALNG0041963 | GC05M056729 |
| LOC105370163 | GC13P035856 |
| lnc-FRY-1 | GC13P032297 |
| HSALNG0096212 | GC13P032262 |
| ENSG00000252136 | GC04P105105 |
| RPS16P5 | GC06M053334 |
| HTR3D | GC03P184031 |
| MIR519B | GC19P053695 |
| HSALNG0000137 | GC01P003372 |
| NONHSAG000093.2 | GC01P001354 |
| lnc-DVL1-2 | GC01M005137 |
| piR-35674-001 | GC01M005138 |
| HSALNG0000139 | GC01M005269 |
| HSALNG0000142 | GC01P003489 |
| HSALNG0000138 | GC01P003369 |
| lnc-DVL1-1 | GC01M005136 |
| LOC102467081 | GC05M054979 |
| ENSG00000235725 | GC02M065589 |
| MIR3928 | GC22M031160 |
| MIR4695 | GC01M018883 |
| MIR551B | GC03P168551 |
| ALLC | GC02P003686 |
| ENSG00000288271 | GC09P093021 |
| LINC01232 | GC13M099487 |
| MIR592 | GC07M127058 |
| lnc-TBC1D3K-1 | GC17P050136 |
| LINC02598 | GC12M019999 |
| LINC01305 | GC02P174326 |
| HSALNG0091218 | GC12M052916 |
| DHFRP2 | GC06M061274 |
| GOLGA6L10 | GC15M082339 |
| MIR1288 | GC17P016282 |
| USP17L9P | GC04P009362 |
| FAM135B | GC08M138130 |
| lnc-FOXE1-2 | GC09P097699 |
| DUTP7 | GC04M051865 |
| lnc-LRRC66-2 | GC04M051866 |
| FOXI2 | GC10P127737 |
| MIR516B1 | GC19P053736 |
| piR-52112 | GC04P051844 |
| MIR644A | GC20P034468 |
| piR-57135-004 | GC05M054998 |
| MIR210HG | GC11M000563 |
| lnc-LRRC66-3 | GC04M051841 |
| MIR4766 | GC22M040813 |
| OR2B6 | GC06P076697 |
| MIR761 | GC01M051836 |
| EMX2OS | GC10M117473 |
| NOP56P1 | GC06M028783 |
| MIR670 | GC11P043752 |
| MIR603 | GC10P024275 |
| MS4A10 | GC11P060789 |
| MIR519C | GC19P053686 |
| OR2B2 | GC06M061068 |
| HSALNG0070399 | GC09P022089 |
| OR14J1 | GC06P029301 |
| MIR922 | GC03M197674 |
| OR2W3 | GC01P247895 |
| STATH | GC04P069995 |
| LINC00491 | GC05M102608 |
| RPPH1 | GC14M021842 |
| HSALNG0091217 | GC12P052902 |
| MIR378G | GC01M094745 |
| LINC02243 | GC0XP134953 |
| HSALNG0017407 | GC02M102235 |
| MICC | GC06P076834 |
| HSALNG0028711 | GC03P126938 |
| LINC01544 | GC18P061748 |
| DHRS4L2 | GC14P030879 |
| MIR516B2 | GC19P053725 |
| RPTN | GC01M152153 |
| ARMCX5 | GC0XP102602 |
| piR-59412-008 | GC04P105132 |
| RF00017-7053 | GC09P022061 |
| MIR624 | GC14M031014 |
| MIR2682 | GC01M098045 |
| ENSG00000275586 | GC07P148876 |
| MK279980 | GC01M155212 |
| lnc-ACTR2-9 | GC02P065580 |
| NONHSAG008235.2 | GC11P047354 |
| MIR933 | GC02M175167 |
| VCY | GC0YM013986 |
| HCG26 | GC06P076855 |
| piR-32718 | GC03P126959 |
| HSALNG0124498 | GC19M017280 |
| FMO6P | GC01P171106 |
| UQCRHP1 | GC06M061306 |
| OR6B3 | GC02M240045 |
| OR12D3 | GC06M029373 |
| LOC109433677 | GC14P053953 |
| ICMT-DT | GC01P006240 |
| SCARNA23 | GC0XP024744 |
| RF00017-6324 | GC07M148865 |
| lnc-TFAP2A-3 | GC06M010405 |
| MIR610 | GC11P028056 |
| LINC01324 | GC03M164664 |
| LOC117134593 | GC0XP015600 |
| KHSRPP1 | GC09P021696 |
| ENSG00000226668 | GC09P093031 |
| UBQLN1P1 | GC06M030358 |
| LOC642943 | GC09P092882 |
| OR2J2 | GC06P029170 |
| LINC02487 | GC06M167681 |
| LOC112268466 | GC04M052421 |
| LINC01327 | GC03P167392 |
| lnc-SPRED2-13 | GC02M065578 |
| RPS2P1 | GC20P034122 |
| RPS12P15 | GC08P102504 |
| AB372660-005 | GC06M010404 |
| HSALNG0043609 | GC05M095622 |
| DPYD-AS2 | GC01P097796 |
| LINC02153 | GC08P020974 |
| BA000025-002 | GC06P077412 |
| MIR4497 | GC12P109833 |
| HSALNG0075830 | GC10M004977 |
| CYLC2 | GC09P102995 |
| HSALNG0057698 | GC07M044951 |
| VTRNA2-2P | GC02M065555 |
| ENSG00000224251 | GC10P004997 |
| HSALNG0007878 | GC01M160908 |
| LOC112267933 | GC05M095623 |
| CYHR1 | GC08M145254 |
| MIR512-2 | GC19P062495 |
| MIR3651 | GC09M092292 |
| ASB17 | GC01M075918 |
| FKBP9P1 | GC07M055683 |
| LINC02893 | GC09M091443 |
| HSALNG0017409 | GC02P102242 |
| EZR-AS1 | GC06P158817 |
| MIR518E | GC19P053729 |
| LOC100500719 | GC22M036537 |
| ENSG00000188078 | GC22M036538 |
| LINC02200 | GC05P112629 |
| SMIM45 | GC22P043135 |
| MIR6515 | GC19P012940 |
| LINC00620 | GC03P013625 |
| HSALNG0094046 | GC12M111619 |
| PKD3 | GC02U990077 |
| MIR1285-2 | GC02M070252 |
| MIR4458 | GC05P008460 |
| HSALNG0084051 | GC11M085247 |
| SEMG2 | GC20P045221 |
| SCARNA2 | GC01P109176 |
| ENSG00000287701 | GC05M074898 |
| lnc-CHRNB4-3 | GC15M078618 |
| NONHSAG017570.2 | GC15P078618 |
| SNORA24 | GC04P118279 |
| HSALNG0038561 | GC04M172043 |
| RPL17P25 | GC06M080373 |
| KRT18P33 | GC02P065666 |
| RPL12P36 | GC16P058825 |
| MK280046 | GC11P047358 |
| LOC111162621 | GC03P189786 |
| MIR7109 | GC22M031621 |
| MIR570HG | GC03P196163 |
| RPSAP72 | GC06M080471 |
| piR-50417-023 | GC02P065624 |
| PARTICL | GC02M085540 |
| UBE2NL | GC0XP143884 |
| lnc-ATP6V1G2-DDX39B-3 | GC06M061283 |
| ENSG00000233902 | GC06M031462 |
| HSFY2 | GC0YM018733 |
| AKR1C6P | GC10M004871 |
| MIR4282 | GC06M072967 |
| piR-48348-078 | GC07M138677 |
| RF00017-6266 | GC07P138651 |
| RF00017-4354 | GC05M074925 |
| OR3A2 | GC17M003969 |
| MIR3143 | GC06P076649 |
| lnc-SELENOP-2 | GC05M042229 |
| NONHSAG009311.2 | GC11P089284 |
| LINC01711 | GC20P058635 |
| LINC01149 | GC06P076854 |
| MIR1324 | GC03P075630 |
| MIR4306 | GC13P099643 |
| REXO1L1P | GC08M085656 |
| MIR1323 | GC19P062490 |
| HSALNG0067440 | GC08M102526 |
| ENSG00000276302 | GC06P028267 |
| AKAP2 | GC00U936898 |
| HSALNG0073137 | GC09M097767 |
| piR-43083-011 | GC05P042292 |
| HSALNG0067439 | GC08P102522 |
| MIR1243 | GC04P113106 |
| MIR602 | GC09P137838 |
| RPL27AP5 | GC05M074990 |
| piR-55654-523 | GC09P097796 |
| piR-32214-546 | GC05P042248 |
| RN7SL339P | GC05P171359 |
| MIR6800 | GC19P049832 |
| MIR451B | GC17P028861 |
| MIR3612 | GC12P128294 |
| MIR4261 | GC02M010192 |
| H3P30 | GC09P021640 |
| PCDHB17P | GC05P145723 |
| RPS8P3 | GC18M051844 |
| RNU6-1252P | GC04P052494 |
| SNX18P2 | GC09M092893 |
| TARDBPP1 | GC20M007894 |
| FAM183DP | GC02M102249 |
| MTHFD2P6 | GC05M041967 |
| TMEM97P2 | GC08M020932 |
| CICP28 | GC07P056805 |
| lnc-CCM2-3 | GC07P044949 |
| LHFPL3-AS1 | GC07M104738 |
| MIR450A2 | GC0XM134637 |
| CTXND2 | GC01P151116 |
| ACTG1P25 | GC01P202864 |
| TSTD3 | GC06P099452 |
| LINC01672 | GC01P006920 |
| MIR548L | GC11M094466 |
| LINC01014 | GC03P178419 |
| LOC111162620 | GC03P189630 |
| MIR4469 | GC08M042896 |
| MIR133A1HG | GC18M023351 |
| MIR617 | GC12M080832 |
| lnc-AKR1C3-4 | GC10P004980 |
| piR-54987-007 | GC10P004986 |
| MIR6125 | GC12P062261 |
| PMPCAP1 | GC04M092181 |
| OR51B2 | GC11M006246 |
| MIR4636 | GC05M009055 |
| OR2T5 | GC01P248488 |
| MIR6743 | GC11P000229 |
| MIR4701 | GC12M048774 |
| ZNF676 | GC19M031701 |
| LOC118966792 | GC0XP015580 |
| MIR5587 | GC16P000535 |
