# Supplementary material for: Effect of Cepharanthine on the Stemness of Lung Squamous Cell Carcinoma Based on Network Pharmacology and Bioinformatics
Source: Biomed Res Int. 2022 Nov 28;2022:5956526. doi: 10.1155/2022/5956526 (PMC9723418; doi:10.1155/2022/5956526)
Supplement: Supplementary 3 — Table S3 Topological parameters of network nodes. [file 5956526.f3.docx]

**Table S3 Topology parameters of network nodes.**

| **Node** | **MCC** | **DMNC** | **MNC** | **Degree** | **Closeness** | **Betweenness** |
| --- | --- | --- | --- | --- | --- | --- |
| CCNA2 | 21726 | 0.63062 | 14 | 14 | 23.33333 | 69.01711 |
| CHEK1 | 21678 | 0.6642 | 13 | 13 | 22.66667 | 49.7624 |
| CDK1 | 21650 | 0.62588 | 13 | 13 | 22.41667 | 63.67497 |
| AURKA | 21600 | 0.7466 | 11 | 11 | 21.25 | 21.65441 |
| PLK1 | 20880 | 0.77815 | 10 | 10 | 20.41667 | 15.05591 |
| KIF11 | 20184 | 0.73825 | 10 | 10 | 18.91667 | 6.1 |
| CCNE1 | 11546 | 0.60003 | 12 | 12 | 21.91667 | 46.77084 |
| NEK2 | 10086 | 0.69213 | 9 | 9 | 18.83333 | 11.46405 |
| CCNA1 | 10080 | 0.78725 | 8 | 8 | 17.75 | 0.25 |
| PLK4 | 10080 | 0.78725 | 8 | 8 | 17.75 | 0.25 |
| CASP3 | 1618 | 0.45045 | 14 | 16 | 25.16667 | 389.41518 |
| EGFR | 969 | 0.47297 | 14 | 15 | 24 | 171.11005 |
| MMP9 | 212 | 0.42441 | 12 | 12 | 22.33333 | 83.45229 |
| MAPK1 | 109 | 0.45346 | 9 | 10 | 21.16667 | 159.52526 |
| MMP1 | 108 | 0.5854 | 7 | 7 | 18.58333 | 3.38095 |
| MET | 78 | 0.51223 | 7 | 7 | 19 | 11.49365 |
| MMP3 | 78 | 0.51223 | 7 | 7 | 19 | 15.26032 |
| PLAU | 61 | 0.47564 | 7 | 8 | 19.58333 | 44.12395 |
| DHFR | 51 | 0.47549 | 6 | 7 | 18.58333 | 79.27364 |
| GSR | 18 | 0.26242 | 8 | 8 | 19.5 | 164.51938 |
| NQO1 | 15 | 0.25611 | 7 | 8 | 19.66667 | 149.11635 |
| MMP13 | 12 | 0.47366 | 4 | 4 | 16.5 | 0.42857 |
| G6PD | 9 | 0.37893 | 4 | 5 | 15.61667 | 27.1 |
| FGFR2 | 8 | 0.37893 | 4 | 4 | 16.75 | 1.84641 |
| GSTP1 | 7 | 0.46346 | 3 | 4 | 17.41667 | 30.46817 |
| GLO1 | 6 | 0.46346 | 3 | 3 | 13.73333 | 0 |
| GSTA1 | 6 | 0.46346 | 3 | 3 | 14.28333 | 0 |
| MMP12 | 6 | 0.46346 | 3 | 3 | 15 | 0 |
| SORD | 6 | 0.46346 | 3 | 3 | 13.73333 | 0 |
| AKR1C1 | 4 | 0.30898 | 3 | 3 | 13.9 | 2.33333 |
| HPRT1 | 4 | 0.30779 | 2 | 4 | 17.41667 | 50.78528 |
| DCK | 3 | 0.30779 | 2 | 3 | 15.53333 | 12.36753 |
| AKR1C3 | 2 | 0.30779 | 2 | 2 | 13.06667 | 0 |
| RARG | 2 | 0 | 1 | 2 | 13.83333 | 68 |
| CRABP2 | 1 | 0 | 1 | 1 | 10.1 | 0 |
| HTR7 | 1 | 0 | 1 | 1 | 12.08333 | 0 |
| LGALS7 | 1 | 0 | 1 | 1 | 1 | 0 |
| LGALS7B | 1 | 0 | 1 | 1 | 1 | 0 |
