# Supplementary material for: Effect of Cepharanthine on the Stemness of Lung Squamous Cell Carcinoma Based on Network Pharmacology and Bioinformatics
Source: Biomed Res Int. 2022 Nov 28;2022:5956526. doi: 10.1155/2022/5956526 (PMC9723418; doi:10.1155/2022/5956526)
Supplement: Supplementary 4 — Table S4 the binding sites of CEP at the target protein. [file 5956526.f4.docx]

**Table S3** The Binding Sites of Cepharanthine at the Target Protein.

| **Target Protein** | **Type of Interactions** | **Receptor Residue** | **Distance** |
| --- | --- | --- | --- |
| 1mq4  (AURKA) | Hydrophabic Interactions | LYS-143 | 3.44 |
|  |  | PHE-144 | 3.84 |
|  |  | PHE-144 | 3.69 |
|  |  | GLU-260 | 3.77 |
|  | Hydrogen Bonds | LYS-143 | 3.3 |
|  |  | LYS-162 | 3.63 |
|  |  | LYS-258 | 3.92 |
|  |  | TRP-277 | 3.45 |
|  | Salt Bridges | GLU-260 | 5.49 |
| 1fin  (CCNA2) | Hydrophabic Interactions | ILE-182 | 3.58 |
|  |  | ILE-182 | 3.73 |
|  |  | GLN-313 | 3.73 |
|  |  | THR-316 | 3.61 |
|  | Hydrogen Bonds | ASN-173 | 3.31 |
|  | Salt Bridges | GLU-268 | 4.83 |
| 1w98  (CCNE1) | Hydrophabic Interactions | GLN-240 | 3.75 |
|  | Hydrogen Bonds | ASN-236 | 3.07 |
| 4y72  (CDK1) | Hydrophabic Interactions | VAL-227 | 3.35 |
|  |  | VAL-227 | 3.83 |
|  |  | ILE-269 | 3.47 |
|  |  | ILE-269 | 3.33 |
|  |  | TYR-270 | 3.8 |
|  |  | LYS-274 | 3.98 |
|  | Hydrogen Bonds | TYR-270 | 3.49 |
| 1ia8  (CHEK1) | Hydrophabic Interactions | GLU-33 | 3.74 |
|  |  | ALA-34 | 3.63 |
|  | Hydrogen Bonds | TYR-71 | 3.04 |
|  |  | TYR-86 | 4.03 |
|  | Salt Bridges | ASP-139 | 5.38 |
|  |  | GLU-140 | 5.08 |
| 1q4o  (PLK1) | Hydrophabic Interactions | LYS-420 | 3.88 |
|  |  | ASP-438 | 3.56 |
|  |  | LYS-474 | 3.23 |
|  | Hydrogen Bonds | ARG-456 | 2.87 |
|  |  | ARG-456 | 3.84 |
|  | Salt Bridges | ASP-438 | 4.86 |
